# Parent and carer experiences of health care professionals’ communications about a child’s higher weight: a qualitative systematic review

**DOI:** 10.11124/JBIES-24-00056

**Published:** 2025-04-07

**Authors:** Terhi Koivumäki, Maria Kääriäinen, Anna-Maria Tuomikoski, Marja Kaunonen

**Affiliations:** 1 Health Sciences, Faculty of Social Sciences, Tampere University, Tampere, Finland; 2 Finnish Centre for Evidence-Based Health Care: A JBI Centre of Excellence, Nursing Research Foundation, Helsinki, Finland; 3 Research Unit of Nursing Science and Health Management, University of Oulu, Oulu, Finland; 4 Oulu University Hospital, Oulu, Finland; 5 General Administration, Pirkanmaa Hospital District, Tampere, Finland

**Keywords:** childhood obesity, experience, health care professionals, parents/carers, weight communication

## Abstract

**Objective:**

The objective of this systematic review was to identify, critically appraise, and synthesize the best available qualitative evidence on parent and carer experiences of communications from health care professionals concerning their child’s higher weight.

**Introduction:**

Public discourse on obesity has shifted in recent years and created pressure to change the way that weight is discussed in health care. A child’s higher weight can be a sensitive issue to discuss in health care, but successful communication with parents can increase parental compliance with treatment and improve overall family welfare. It is, therefore, important to explore how parents and carers experience the communication about children’s higher weight to ensure effective, up-to-date, and ethical counseling on childhood obesity.

**Inclusion criteria:**

This qualitative review included studies that focused on the experiences of parents or carers of children (birth to 12 years) with a higher weight who received verbal or written communication from health care professionals about their child’s weight.

**Methods:**

The following databases were systematically searched from 2010 onward: MEDLINE (EBSCOhost), CINAHL (EBSCOhost), PsycINFO (Ovid), Scopus, LILACS, and the Finnish health sciences database MEDIC. ProQuest Dissertations and Theses (ProQuest) was searched for unpublished articles. The search was conducted in July 2022 and updated in October 2023. No country or language limits were applied. A manual search was used to supplement the database searches. Study selection including title and abstract screening, full-text screening, critical appraisal, and data extraction were performed by 2 reviewers. The research findings were categorized and aggregated into synthesized findings. The synthesized findings were assigned confidence scores, and categories and finalized synthesized findings were agreed upon by all reviewers.

**Results:**

The 33 included studies varied in qualitative study design and methodological quality. There were over 900 eligible participants (parents and carers) and 147 unequivocal and credible research findings. The research findings yielded 8 categories and 3 synthesized findings with low confidence scores. The synthesized findings were as follows: i) Parents receiving communication on a child’s higher weight experience strong feelings that can affect their parenting; ii) A health care professional’s active and individual communication, with the sensitive use of words, creates a good communication experience for parents; and iii) Parents want to receive information about the child’s higher weight that is useful to them and is based on an acceptable weight estimation.

**Conclusion:**

Although confidence in the synthesized findings is low, this review indicates that communication from a health care professional on a child’s higher weight should meet the parents’ expectations and the family’s situation and needs. Having the skills to deal with heightened emotions, using expertise and empathy as a professional, and providing appropriate information create a good communication experience for parents. In addition, parents’ desire to protect their child and the strengthening of the parenting experience should be acknowledged to conduct safe communication.

**Supplemental digital content:**

A Finnish-language version of the abstract of this review is available at: http://links.lww.com/SRX/A92.

**Table:** ConQual Summary of Findings

Parent and carer experiences of health care professionals’ communications about a child’s higher weight					
Bibliography: Koivumäki T, Kääriäinen M, Tuomikoski A-M, Kaunonen M. Parent and carer experiences of health care professionals’ communications about a child’s higher weight: a qualitative systematic review. JBI Evid Synth. 2025;23(4):576-637.					
**Synthesized finding**	**Type of research**	**Dependability**	**Credibility**	**ConQual score**	**Comments**
Parents receiving communication on a child’s higher weight experience strong feelings that can affect their parenting.	Qualitative	Moderate (Downgrade 1 level)	Moderate (Downgrade 1 level)	Low	Dependability: majority of the studies (16/20) had 3 “yes” responses for the questions related to appropriateness of the conduct of the research. Credibility: downgraded 1 level due to mix of unequivocal (U) and credible (C) findings. U = 28, C = 16.
A health care professional’s active and individual communication, with the sensitive use of words, creates a good communication experience for parents.	Qualitative	Moderate (Downgrade 1 level)	Moderate (Downgrade 1 level)	Low	Dependability: majority of the studies (19/24) had 3 “yes” responses for the questions related to appropriateness of the conduct of the research. Credibility: downgraded 1 level due to mix of unequivocal (U) and credible (C) findings. U = 47, C = 13.
Parents want to receive information about the child’s higher weight that is useful to them and is based on an acceptable weight estimation.	Qualitative	Moderate (Downgrade 1 level)	Moderate (Downgrade 1 level)	Low	Dependability: majority of the studies (12/18) had 3 “yes” responses for the questions related to appropriateness of the conduct of the research. Credibility: downgraded 1 level due to mix of unequivocal (U) and credible (C) findings. U = 36, C = 7.

## Introduction

In health care, communication plays an important role in successful treatment. Good communication is positively correlated with patient adherence^1^ and patient-centered care, and includes sufficient information-sharing, opportunities to participate in decision-making,^2^ and motivational interviewing.^3^ A child’s higher weight is a sensitive issue for parents, and as both body weight and parenthood are emotionally charged themes, there is a clear need to understand parental experiences of communication related to a child’s higher weight.

There is no consensus on the best words to use when referring to a child’s higher weight. There is a joint statement from several European obesity organizations encouraging people-first language when discussing a child’s weight (eg, children with obesity).^4^ However, some disciplines and organizations resist this, as people-first language is recommended for use with illness or disability, and there is no common opinion as to whether higher weight can be labeled as either of those. A systematic review by Puhl *et al.*^5^ found that neutral terminology (eg, weight or unhealthy weight) is preferred, and hence the term *higher weight* was chosen for this review.

A child’s higher weight and weight stigma can both have consequences on a child’s present or future health. According to the World Health Organization, the prevalence of higher weight among children and adolescents aged 5–19 years has risen from 8% in 1990 to 20% in 2022.^6^ Having a higher weight can affect children’s and adolescents’ immediate health. It is also associated with a greater risk and earlier onset of various non-communicable diseases (eg, type 2 diabetes, cardiovascular disease), and the tendency for higher weight often persists into adulthood.^6^ A meta-analysis by Ma *et al.^7^* found positive concurrent and bidirectional relationships between weight stigma and weight status among children aged 6–18 years. At worst, weight communication, especially if it increases weight stigma experiences, can lead to adverse physiological and psychological outcomes with individuals^8^ or feelings of incapable parenting.^9^ Almost one-fourth of parents would avoid future medical appointments if they felt that they or their children were stigmatized by doctors because of the child’s weight.^10^ Therefore, providing effective and ethical communication related to a child’s weight enables parents to create a healthy living environment for their child without increasing the stigma experienced by either the child or the parent.

In the assessment of body weight within health care, the body mass index (BMI) is the most commonly utilized method due to its simplicity and cost-effectiveness.^11^ In children, BMI measurements are standardized according to age and sex to account for growth patterns. For instance, the American Academy of Pediatrics recommends the use of BMI in clinical practice to identify children with higher weight.^12^ However, the use of BMI as a measure for evaluating weight, and particularly health, has long been subject to criticism. Although BMI has proven useful in population-based studies, it is not ideally suited as an indicator for individual weight assessment.^13^ The primary reasons for these critiques are the BMI’s inability to differentiate between lean and fat mass, its lack of indication regarding body fat distribution,^11^ and its failure to account for gender or ethnic differences.^13^ This discrepancy may, in part, challenge communication between professionals and parents concerning the child’s weight.

Health care professionals (HCPs) and parents acknowledge that a child’s higher weight is a sensitive topic to raise,^14^ and could even be considered a health communication dilemma.^15^ There is a range of factors that could impact the parents’ emotional responses when discussing a child’s weight, such as demographic, parental (underestimation of children’s weight or perceptions of weight management), and HCP factors (attitudes and practice), as well as mass media.^15^ There is strong evidence that parents do not always recognize their child’s higher weight correctly,^16^-^19^ and this can create tensions in communication between HCPs and parents. In addition, raising the issue of the child’s higher weight can cause negative feelings for parents, regardless of whether it is done face to face or as written feedback.^20^ While interventions have been implemented to test communication via telephone calls^21^ and text messages,^22^ further research is needed to explore parents’ experiences with different forms of communication regarding their child’s weight.

Parents’ hesitation to communicate regarding the child’s weight may be due to a desire to protect their child from feeling stigmatized.^23^ Parents’ experiences of their own body weight^24^ or parental self-assessment of their own skills and strengths^25^ could affect how messages regarding the child’s weight are received. One study showed that parents preferred positive and non-judgmental conversations.^26^ HCPs have their own hesitations about weight communication. There are several barriers and facilitators for HCPs when discussing the child’s weight with parents, such as intra/interpersonal-level factors and factors at the organizational and societal levels.^27^ In addition, HCPs feel that they lack knowledge on how to communicate regarding a child’s weight.^28^ Furthermore, policy documents developed by health authorities may be based on outdated and stigmatizing perspectives on obesity,^29^ potentially undermining the HCP’s ability to engage in appropriate discussions about weight issues with parents.

Previous syntheses have examined communication between parents and HCPs regarding a child’s weight. However, studies focusing solely on parents’ experiences are scarce, particularly among parents of children with higher weight. Ames *et al.*^20^ conducted a mixed methods systematic review that explored the most effective ways to notify parents and children about the child’s weight, as well as parents’ preferences and experiences around weight notification. In this review, parents had clear preferences for the format, timing, content, and amount of information they wanted, and how they wanted HCPs to interact and communicate with them. A scoping review by McPherson *et al.*^30^ emphasized that including all stakeholders in discussions is important, as are early and regular communications, strength-based language that emphasizes health rather than weight, collaborative goal-setting, and the use of appropriate tools and resources. In addition, when interviewing children and caregivers with or without disabilities, McPherson *et al.*^31^ found that discussions emphasizing growth and health were preferred to weight and size. Strengths-based, solution-focused approaches for weight conversations were endorsed, although they had not been widely experienced.^31^ Even though previous research has examined communication between HCPs and parents regarding a child’s higher weight, there is a lack of studies focusing exclusively on qualitative research and parents of children with higher weight. Both qualitative data and parents’ personal experiences of a child’s higher weight are important when researching a sensitive issue such as a child’s weight.

As childhood obesity rates continue to increase,^6^ how parents are confronted with the issue of a child’s weight in health care matters. There are recommendations and research-based information on how to communicate with parents regarding a child’s weight. In 2007, an expert committee provided recommendations regarding the prevention, assessment, and treatment of child and adolescent overweight and obesity. These recommendations emphasize the use of patient-centered communication and motivational interviewing. Additionally, there are examples of language on how to conduct an effective conversation during an office visit that focuses on obesity prevention.^32^ Parents show preference for language and words that are familiar to them; for example, both parents and HCPs responded positively to the terms *healthier weight* and *above a healthy weight*, but the term *BMI* is more commonly used and accepted among HCPs.^33^ Emphasizing autonomy and open-ended questions and making reflections are more likely to elicit change talk, for example, among minority families.^34^ In collaborations between clinicians and parents, a positive relationship, the negotiation of the health care delivery, and regular monitoring and evaluation are important to optimize pediatric weight management.^35^

Public discourse on obesity has been changing in recent years. Despite the persistence of weight stigma in mass media and public health practices, there is a growing trend toward more sensitive and neutral representations of obesity. This shift includes a greater focus on the obesogenic environment rather than individual characteristics.^36^ In the context of childhood obesity, parents, particularly mothers, have historically been depicted as the primary culprits in family magazines^37^ and on social media.^38^,^39^ Communication about childhood obesity on social media has concentrated more on individual behaviors than on environmental or policy factors.^40^ Although the discourse on obesity has gradually evolved and the impact of weight stigma is increasingly acknowledged in public discussions, it is important to explore parents’ experiences of communication about their child’s weight in the health care context. This is particularly relevant as parents continue to face weight stigma by association, which can influence their perceptions of communication from HCPs.

The quality and effectiveness of communication about the child’s weight between parents and HCPs are important factors in every phase of pediatric weight prevention and management. However, the best practices related to evidence-based, weight-related communication are still lacking.^30^ While we recognize that the impact of weight feedback on behavior change is limited, we need more research to help to identify ways to communicate more effectively with parents and to determine what type of information and support helps parents to make and maintain lifestyle changes in their family.^17^

This review aimed to enhance understanding of how to better support parents of children with higher weight, with a particular focus on the parents’ experiences. By concentrating on these experiences, the review sought to provide valuable insights into effective support strategies. Prior to conducting this systematic review, a preliminary search of PROSPERO and *JBI Evidence Synthesis* was conducted and no current or in-progress systematic reviews on the topic were identified. The objective of this review was to identify, critically appraise, and synthesize the best available qualitative evidence about the experiences of parents and carers concerning HCPs’ communication about a child’s higher weight.

## Review question

What are parents’ and carers’ experiences of HCPs’ communication regarding their child’s higher weight?

## Inclusion criteria

### Participants

This qualitative review considered studies that included mothers, fathers, or other carers (such as grandparents or other close caregivers) of a child aged from birth to 12 years with a higher weight. To reduce repetition, *parents* refers also to carers, as defined previously. *Higher weight* refers to childhood overweight or obesity that has been diagnosed/recognized in health care using indicators within that country (eg, BMI-for-age, growth curves). The age limit was established to focus this review on childhood obesity prior to puberty, a period during which parental influence on a child’s daily life is significant. Despite cultural variations, the onset of puberty typically occurs in children around 12 years of age.^41^,^42^ Puberty alters body composition; thus, this review primarily focused on children’s body weight prior to the onset of puberty. Furthermore, parents exert greater influence on their child’s life and lifestyle habits before adolescence. Numerous studies investigating parental influence on children’s lifestyle habits predominantly focus on children under the age of 12.^43^,^44^ As this review focuses on the parental perspective, the age limit was established to encompass the period during which parents are actively involved in their child’s life, including discussions about weight. Studies were excluded when the participants were the parents of both normal- and higher-weight children and the parents’ experiences could not be separated, meaning that there was no way to extract the results from parents with a child with higher weight only. In addition, a study was included if the mean age of the children was under 12 years, even though some children may have been over 12 years. The mean age of children was calculated and mentioned when possible.

### Phenomenon of interest

This review considered studies that described parents’ or carers’ experiences of communication with HCPs concerning their child’s higher weight. Experiences related to emotions, reactions, perceived benefits and harms, and the language used were the focus of this review. The communication could be initiated by both parents and HCPs, provided that the child’s weight is taken into consideration. Communication included verbal or written communication about a child’s weight from the HCP that was received by the parent or carer of a child with a higher weight. If the study included communication between parents and HCPs but did not concern the child’s weight, it was excluded. *HCP* refers to all professionals working in health care (eg, nurses, doctors, dietitians) or people promoting family health and working with HCPs (eg, promotoras).

### Context

The review considered studies that researched parents’ experiences in a health care setting (primary or secondary care) where the main reason for the meeting was the prevention of or care for childhood obesity. The main reason was determined based on the purpose of the meeting, which had to be related to the child’s weight. If the context was a school, non–health care facility, community program, or public health initiative, the communication had to be conducted by an HCP in order to be considered for inclusion.

### Types of studies

This review considered studies that focused on qualitative data, including, but not limited to, designs such as phenomenology, grounded theory, ethnography, action research, and mixed methods qualitative data. Descriptive qualitative studies that describe the experiences of parents and carers were also included. Quantitative studies, editorials, commentaries, letters, and conference abstracts were excluded. Studies published from 2010 were included, as this review was particularly focused on relatively recent experiences since the advent of new obesity discourse. In addition, McPherson *et al.*^30^ discovered that the majority of articles about obesity communication were published in or after 2010, so this date was set as the lower date limit.

## Methods

This systematic review was conducted in accordance with the JBI methodology for systematic reviews of qualitative evidence^45^ and followed a published a priori protocol.^46^ It is registered with PROSPERO (CRD42022297709).

### Search strategy

The search strategy (Appendix I) aimed to locate both published and unpublished studies from 2010 onward. The databases searched included MEDLINE (EBSCOhost), CINAHL (EBSCOhost), PsycINFO (Ovid), Scopus, LILACS, and the Finnish health sciences database MEDIC. The search for unpublished studies and gray literature was conducted in ProQuest Dissertations and Theses (ProQuest). When conducting the searches, assistance from an information specialist was utilized. Keywords used in searches were based on the reviewers’ knowledge and the suggestions of the information specialist. The search strategy, including all identified keywords and index terms, was adapted for each database/information source. After the initial search and selection in 2022, the reference lists of all included studies were screened for potential additional studies. Database searches were conducted in July 2022 and updated in October 2023. In the updated search, only 1 study was added to the review,^47^ as it was published after the first search. There were 2 potential studies (an article and a dissertation) that could have met the inclusion criteria, but the full texts could not be retrieved, even with the assistance of the university library. Both studies were published in 2023 and were not yet open access.

This review considered studies from all geographic settings and there were no language limits. After the database searches in 2022 and 2023, the search revealed several studies where the main language was one other than English. In these cases, there was a title and abstract in English, which enabled the research team to make a decision as to whether to include or exclude it. In a few cases, the abstract was in Spanish, but with the help of one author’s (TK) knowledge of Spanish and free language translators such as Google Translate, the decision of whether to include or exclude the study could be made. Only 1 of the eligible studies was in a language other than English (Norwegian),^48^ but it had an English abstract that aided in the decision to select it for full-text review. The article was translated into Finnish (the authors’ native language) with the help of Google Translate and the authors’ knowledge of Swedish (Swedish and Norwegian are similar languages). Texts from this Norwegian article that were chosen for this review were then translated into English, and the translations were verified by a native Norwegian speaker.

### Study selection

Following the search, all identified citations were collated and uploaded into Covidence (Veritas Health Innovation, Melbourne, Australia) and duplicates were removed. Titles and abstracts were screened by 2 independent reviewers (TK and AT, or MKa, or MKä) for assessment against the inclusion criteria for the review. Potentially relevant studies, both from the database search and citation searching, were retrieved in full and their citation details imported into the JBI System for the Unified Management, Assessment and Review of Information (JBI SUMARI, Adelaide, Australia).^49^ Differences between the reviewers’ screening assessments were discussed and resolved through consensus. The full texts of the selected citations were assessed in detail against the inclusion criteria by 2 independent reviewers (TK and AT, or MKa, or MKä). Reasons for the exclusion of papers at full-text screening that did not meet the inclusion criteria were recorded and reported in the systematic review (Appendix II). Any disagreements that arose between the reviewers at each stage of the selection process were resolved through discussion among all reviewers. Before title/abstract screening and full-text review, a pilot test with a small number of articles (n = 5) was conducted with all the reviewers (TK, AT, MKa, and MKä) to ensure the consistency of the study selection process.

### Assessment of methodological quality

Eligible studies were critically appraised for methodological quality by 2 independent reviewers (TK and AT, or MKa, or MKä) using the JBI critical appraisal checklist for qualitative research.^45^ Any disagreements were resolved through discussion between all the reviewers. The JBI critical appraisal checklist includes 10 questions that are applied to each study and represent criteria concerning study methodology, methods, and findings; research ethics; and researcher influence on the research (Table 1). Possible responses to the questions were “yes,” “no,” or “unclear,” with “yes” meaning clear evidence of criterion support, “no” meaning no evidence of criterion support, and “unclear” meaning some evidence of criterion support, but detail or explanation was missing. A decision was made in the protocol phase of this review that all studies, regardless of their methodological quality, would undergo data extraction and synthesis (where possible).

**Table 1:** Critical appraisal of eligible studies

Study	Q1	Q2	Q3	Q4	Q5	Q6	Q7	Q8	Q9	Q10	Score
1. Akselbo, 2015^48^	U	Y	Y	Y	Y	N	N	Y	Y	Y	7/10
2. Anderson *et al*., 2021^51^	U	Y	Y	Y	Y	N	N	Y	Y	Y	7/10
3. Åsberg *et al*., 2023^47^	Y	Y	Y	Y	Y	U	Y	Y	Y	Y	9/10
4. Ayash, 2011^52^	N	Y	Y	Y	Y	N	N	Y	Y	Y	7/10
5. Banks *et al*., 2014^53^	N	Y	Y	Y	Y	N	N	Y	Y	Y	7/10
6. Davidson *et al*. 2017^54^	N	Y	Y	Y	Y	N	N	Y	Y	Y	7/10
7. Ek *et al*., 2020^25^	Y	Y	Y	Y	Y	Y	Y	Y	Y	Y	10/10
8. Eli *et al*., 2022^55^	Y	Y	Y	Y	Y	Y	Y	Y	Y	Y	10/10
9. Falbe *et al*., 2017^56^	N	Y	Y	Y	Y	N	N	Y	Y	Y	7/10
10. Farnesi *et al*., 2012^35^	N	Y	Y	Y	Y	N	N	Y	Y	Y	7/10
11. Gainsbury *et al*., 2018^57^	Y	Y	Y	Y	Y	N	Y	Y	Y	Y	9/10
12. Gillison *et al*., 2014^58^	U	Y	Y	Y	Y	N	N	Y	Y	Y	7/10
13. Gorlick *et al*., 2021^59^	N	Y	Y	Y	Y	N	N	Y	Y	Y	7/10
14. Hardy *et al*., 2019^60^	N	Y	Y	Y	Y	Y	Y	Y	Y	Y	9/10
15. Jones *et al*., 2014^61^	N	Y	Y	Y	Y	N	N	Y	Y	Y	7/10
16. Jorda, 2017^62^	Y	Y	Y	Y	Y	Y	Y	Y	Y	Y	10/10
17. Laurent *et al*., 2014^63^	U	Y	Y	Y	Y	N	N	Y	Y	N	6/10
18. Lupi *et al*., 2014^64^	N	Y	Y	Y	Y	N	N	Y	Y	Y	7/10
19. Morenz-Harbinger, 2013^65^	Y	Y	Y	Y	Y	Y	Y	N	Y	Y	9/10
20. Moyer *et al*., 2014^66^	N	Y	Y	Y	Y	N	N	Y	Y	Y	7/10
21. Nnyanzi *et al*., 2016^67^	N	Y	Y	Y	Y	N	N	Y	Y	Y	7/10
22. Pena *et al*., 2021^68^	Y	Y	Y	Y	Y	Y	N	Y	Y	Y	9/10
23. Povey *et al*., 2020^69^	Y	Y	Y	Y	Y	N	N	Y	Y	Y	8/10
24. Schalkwijk *et al*., 2015^70^	N	Y	Y	Y	Y	N	U	Y	Y	Y	7/10
25. Schwartz, 2015^71^	Y	Y	Y	Y	Y	N	N	N	Y	Y	7/10
26. Syrad *et al*., 2015^72^	Y	Y	Y	Y	Y	Y	Y	Y	Y	Y	10/10
27. Toftemo *et al*., 2013^73^	N	Y	Y	Y	Y	U	N	Y	Y	Y	7/10
28. Turer *et al*., 2016^74^	N	Y	Y	Y	Y	N	U	Y	Y	Y	7/10
29. Turer *et al*., 2016^75^	N	Y	Y	Y	Y	N	N	Y	Y	Y	7/10
30. Turner *et al*., 2012^76^	N	Y	Y	Y	Y	N	N	Y	Y	Y	7/10
31. Visram *et al*., 2013^77^	N	Y	Y	Y	Y	U	N	Y	Y	Y	7/10
32. Wagner *et al*., 2022^78^	N	U	U	U	U	Y	U	U	Y	U	2/10
33. Wild *et al*., 2020^79^	U	Y	Y	Y	Y	U	U	Y	Y	Y	7/10
Total % Yes scores	30	97	97	97	97	24	24	91	100	94	

Y, yes; N, no; U, unclear

JBI critical appraisal checklist for qualitative research^45^

Q1 = Is there congruity between the stated philosophical perspective and the research methodology?

Q2 = Is there congruity between the research methodology and the research question or objectives?

Q3 = Is there congruity between the research methodology and the methods used to collect data?

Q4 = Is there congruity between the research methodology and the representation and analysis of data?

Q5 = Is there congruity between the research methodology and the interpretation of results?

Q6 = Is there a statement locating the researcher culturally or theoretically?

Q7 = Is the influence of the researcher on the research, and vice-versa, addressed?

Q8 = Are participants, and their voices, adequately represented?

Q9 = Is the research ethical according to current criteria or, for recent studies, is there evidence of ethical approval by an appropriate body?

Q10 = Do the conclusions drawn in the research report flow from the analysis, or interpretation, of the data?

As there are no specific guidelines in the JBI methodology about individual criteria or acceptance criteria,^50^ the authors of this review made the decision to include all sources of evidence without cutoff scores or particular criteria. It was suspected in the preliminary search that studies concentrating only on weight communication would be scarce and that communication on the child’s weight was often not the main aim of the research; hence, data on the inclusion criteria for this review were going to be searched for among very different subject areas. In order not to exclude any study that had findings relevant to our review question, the decision was made not to exclude any studies based on critical appraisal. The aim was to achieve a comprehensive meta-synthesis that accentuated parents’ and carers’ experiences, and by including all the studies at this phase, it minimized the risk of not representing every participant’s voice. Furthermore, as weight communication is a sensitive issue, every parent’s experience was valued.

### Data extraction

Two types of data were extracted from the studies and included in this review: descriptive characteristics of the studies and the authors’ qualitative findings pertaining to the phenomenon of interest. The study characteristics were extracted independently by 2 reviewers (TK and AT, or MKa, or MKä) using the standardized JBI data extraction tool available in JBI SUMARI.^49^ Descriptive characteristics for each study involved details about methodology and methods (data collection and analysis); geographic location; setting/context; phenomenon of interest; participants and sample size (including participants’ ethnicity and gender if mentioned, children’s ages and weight status if mentioned); and research findings according to the author relevant to the review question. If there were differences between the reviewers regarding characteristics extracted, TK reexamined the studies and made the final determination.

The qualitative findings were also extracted independently by 2 reviewers (TK and AT, or MKa, or MKä). Qualitative study findings were defined as verbatim extractions of the authors’ analytic interpretations of their study data, with each finding accompanied by an illustration, if possible, such as a direct participant quotation or field work observation. Weight communication was not always the phenomenon of interest in the studies, hence it was not possible to choose findings only from themes, categories, or other classes created by the original author. The main criteria to choose findings from the studies were that they fit the inclusion criteria and answered the review question. This is why findings vary from themes, subthemes, and categories and so on to titles or subtitles and to parts of the text in the results. The findings were determined through repeated reading of the results alongside examination of other sections of the study for explanatory details, as needed. Qualitative findings were extracted only if they were clearly identifiable as relevant to the participants and phenomenon of interest for this review, that is, if the findings were based on experiences of parents and carers when communicating with HCPs about their child’s higher weight.

Each extracted qualitative finding was assigned 1 of 3 levels of credibility by 2 independent reviewers (TK and AT, or MKa, or MKä). The level of credibility, which refers to the degree of support an illustration offers to the specific finding, may be unequivocal (finding is “accompanied by an illustration that is beyond reasonable doubt and therefore not open to challenge”^(p.94)^), credible (finding is “accompanied by an illustration lacking clear association with it and therefore open to challenge”^(p.94)^), or not supported (“findings are not supported by the data”^(p.94)^).^45^ Differences between reviewers as to whether the extracted material represented a relevant finding and the level of credibility were discussed and resolved through consensus. Some findings had more than 1 illustration (ie, participant quote); in this case, the illustration with the highest level of credibility was chosen.

### Data synthesis

The included qualitative research findings were synthesized using the 3-step JBI meta-aggregation approach.^45^ The first step involved the extraction and assembly of the research findings (with accompanying illustrations and levels of credibility). The second step involved developing categories on the basis of similarity in meaning and assigning fitting category labels. The third step involved aggregating categories on the basis of similarity in meaning to generate a comprehensive set of synthesized findings in the form of statements that represented the aggregated categories and could be used to make recommendations for practice and policy. Although all findings, regardless of credibility level, were extracted from studies, only unequivocal and credible findings were used in the synthesis.

Preliminary categories were developed by TK and the 3 other reviewers (MKä, MKa, and AT) independently. In the end, categories were refined through group discussion and consensus. There was an agreement among all reviewers on the finalized set of synthesized statements and associated recommendations.

### Assessing confidence in the findings

The final synthesized findings were assessed using the JBI ConQual approach^80^ to establish the level of confidence that knowledge users may have in the synthesized findings for informing practice and policy. The ConQual grades are presented in the Summary of Findings. The level of confidence for each synthesized finding was scored as high, moderate, low, or very low.

## Results

### Study inclusion

**Figure 1:**
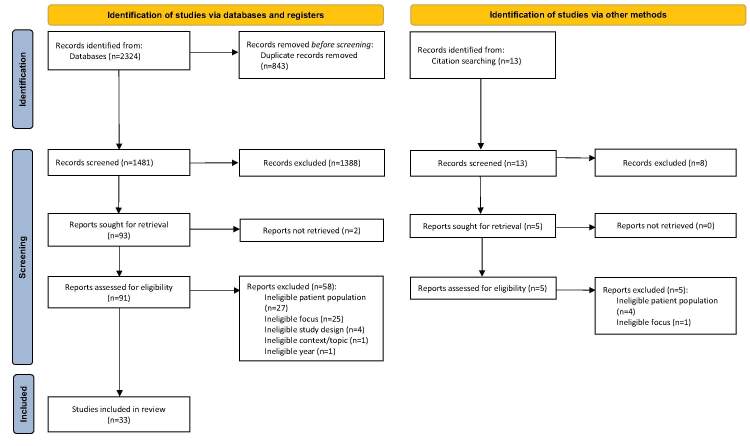
Search results and study selection and inclusion process^81^

The search strategy produced a total of 2337 records from academic database searches (n = 2324) and citation searches (n = 13). The Scopus citation search was included in the gray literature database searches. After the removal of duplicates, a total of 1494 records were retained for title and abstract screening. Through the screening, a total of 1396 were designated as not meeting the inclusion criteria and, in total, 98 studies (including database search n = 93 and citation search = 5) were retained for full-text review. Of those retained, the full-text was not available for 2 studies; 63 were excluded from the review and 33 were assessed as meeting the inclusion criteria. The 2 main reasons for exclusion were i) ineligible patient population (n = 31), meaning that participants were not the parents of children with a higher weight or the parents with normal-weight children and higher-weight children could not be separated in the findings; and ii) ineligible focus (n = 26), meaning that the results did not address communication or weight communication. Other studies were excluded due to ineligible study design (n = 4) or the context of the study being non-health care (n = 1), and 1 study was dated to 2009, even though it was set to 2010 in the search results (n = 1). A list of studies excluded at full-text screening, with reasons for exclusion, is presented in Appendix II. The results of the search and the study inclusion process are presented in a Preferred Reporting Items for Systematic Reviews and Meta-Analyses (PRISMA) flow diagram (Figure 1).^81^

The 33 included records consisted of 30 journal articles and 3 doctoral theses or dissertations. One included study was published in a language other than English and was translated into English and Finnish.

### Methodological quality

The critical appraisal assessment revealed variability in methodological quality across studies (see Table 1). The overall methodological quality was considered moderate, while only 1 (3%) of the studies had low methodological quality, based on achieving “yes” scores on 4 or fewer appraisal criteria.^78^ Ten of the 33 studies (30%) were of high methodological quality achieving “yes” scores in 8–10 of the appraisal criteria. Of those, 4/33 (12%) studies had perfect (10/10) “yes” scores. Twenty-two (67%) were considered to have moderate methodological quality, based on achieving 5–7 “yes” scores.

The most “yes” responses were for Q9 (Is the research ethical according to current criteria or, for recent studies, is there evidence of ethical approval by an appropriate body?), with every study meeting the criteria. In Q2 to Q5, 97% of the studies met the criteria. The most frequently missing criteria were Q6 (Is there a statement locating the researcher culturally or theoretically? “yes” [n = 8; 24%], “unclear” [n = 4; 12%]) and Q7 (Is the influence of the researcher on the research, and vice-versa, addressed? “yes” [n = 8; 24%], “unclear” [n = 4; 12%]). In addition, Q1 (Is there congruity between the stated philosophical perspective and the research methodology?) was not clearly addressed in several studies (“yes” [n = 10; 30%], “unclear” [n = 5; 15%], “no” [n = 18; 55%]).

### Characteristics of included studies

An overview of the characteristics of the included studies is provided in Appendix III. The 33 studies took place in 8 countries: United States (n = 12), United Kingdom (n = 9), Australia (n = 3), Sweden (n = 3), New Zealand (n = 2), Norway (n = 2), Canada (n = 1), and the Netherlands (n = 1).

All of the studies used a qualitative methodology, but they were not always specific; rather, a qualitative method, approach, or evaluation was mentioned. The main methodologies mentioned were phenomenology, grounded theory, and action research. Qualitative methods were used for data collection and analysis, including phenomenological (eg, interpretative phenomenological analysis) or grounded theory methods for data analysis in several of the studies. Many of the 33 studies were described using only generic terminology, such as qualitative study, qualitative approach, qualitative method, qualitative action research, or qualitative evaluation. In 12 studies, the methodologies were described as grounded theory (n = 5) or phenomenology (n = 3), action research (n = 2), or mixed methods designs (n = 2). The most commonly used analysis method was a thematic analysis/approach/framework (n = 17). Content analysis (n = 3), grounded theory (n = 3), and systematic text condensation (n = 2) were used more than once. Other methods used in 1 study were thematic content analysis, interpretative phenomenological analysis, and constant comparative method. The remaining studies used the theoretical constructs of Theory of Planned Behavior, thematic coding, margin coding combined with grounded theory, and action research using themes, units of meaning and categories. One study mentioned only that it used qualitative analysis.

The studies were published from 2011 to 2023 and divided evenly across these years. The most common publishing years were 2014 (n = 6) and 2015 (n = 5), and only 1 year within our search parameters (2010 to the present) had no article published (2010).

The most common method of qualitative data collection was through interviews, including in-person (ie, face-to-face; n = 17), telephone (n = 5), or both (n = 1). Focus groups (n = 5) and focus groups combined with personal interviews (n = 2) were also used. Open answers as qualitative data were used in 2 studies, and 1 study combined phone interviews and open-ended questions. The interviews in person or in focus groups were mostly semi-structured (n = 18), in-depth (n = 4), both semi-structured and in-depth (n = 2), and structured (n = 1). In 1 study, this was not mentioned.

The proposed phenomena of interest varied widely across the included studies. Only a few included weight communication as a phenomenon of interest; rather, communication was a side result. The combining issue was parental/carer experiences and childhood overweight/obesity. The phenomena of interest included weight communication in 5 studies (pediatrician’s communication, language and communication preferences for discussing the child’s weight, child’s weight conversations, discussing child overweight issues, and experiences of health dialogue). The other studies, where communication was a side result, included phenomena of interest such as BMI referral (n = 6), a certain program, the behavior of a certain HCP, treatment, expectation of care, engagement in care, toddlers’ obesity, and weight stigma.

The study samples varied in size from 6 to 219 participants. These participants were the parents or carers of a child with a higher weight. The relevant characteristics of participants according to the inclusion criteria are listed in Appendix III. If the research included parents of normal-weight children and parents with higher-weight children, only parents with higher-weight children were counted. If it was not possible to separate participants according to the child’s weight, this research was not included in the review (see Appendix II for the full-text exclusion list). If the gender of the parent or carer was mentioned, it was listed in Appendix III along with the ethnicity of the participants. While the review considered weight communication experienced by parents or carers, the authors of this review considered children’s ages and weight status to be important characteristics and these were also collected, if available. The mean age of children was listed if it was mentioned or possible to calculate. Most of the children in the studies were aged between 5 and 12 years, with only 3 studies reporting mean ages below 5 years. The weight status of children was expressed as it was mentioned in the original research. If the weight status was not mentioned in the article, there is an explanation in Appendix III as to how the children were determined to have a higher weight.

### Review findings

**Figure 2:**
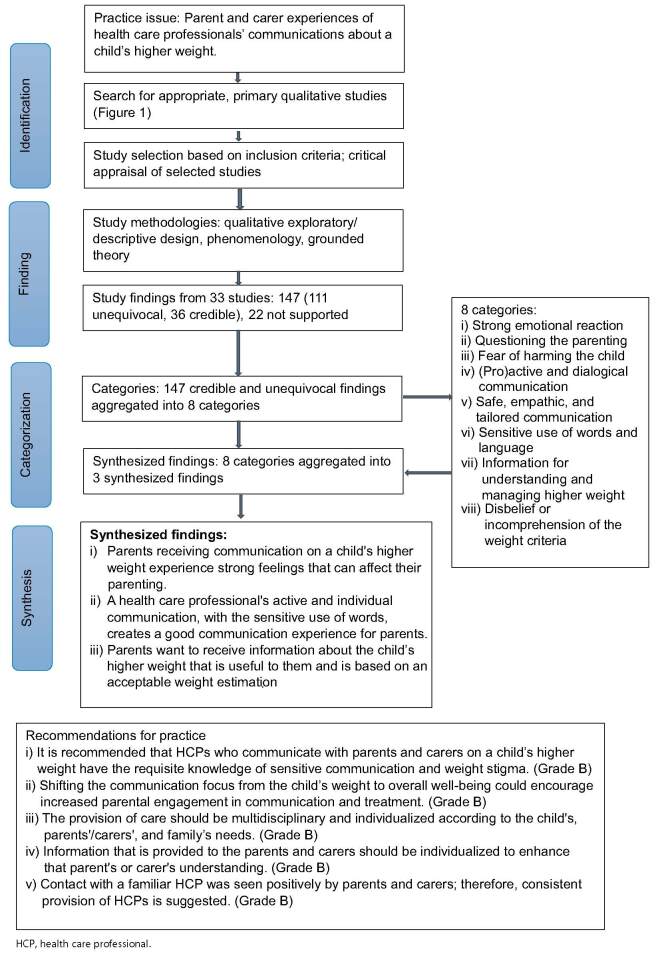
Meta-aggregative process and synthesized findings

The 33 included studies produced a total of 147 qualitative (111 unequivocal, 36 credible) research findings to be included in the synthesized findings (Appendix IV). In addition, 22 were marked as not supported, as the findings were not authenticated with an illustration, and hence they were not included in the synthesis. For example, the doctoral thesis by Morenz-Harbinger^65^ did not include any illustrations, hence none of the findings from that thesis were included in the synthesis. The 147 unequivocal and credible findings were combined into 8 categories and then aggregated into 3 synthesized findings (Figure 2). The synthesized findings were as follows: i) Parents receiving communication on a child’s higher weight experience strong feelings that can affect their parenting; ii) A health care professional’s active and individual communication, with the sensitive use of words, creates a good communication experience for parents; and iii) Parents want to receive information about the child’s higher weight that is useful to them and is based on an acceptable weight estimation.

HCP, health care professional.

There were several findings and illustrations that could have been placed into 2 or more categories. In these situations, to avoid the overlapping of the themes, the final decision on where to place the findings and illustrations was primarily based on respecting the original author’s interpretation. If the original author had set a class, theme, or category, this was used as the determining factor for assignment. To ensure the most appropriate classification, both the original finding and the illustration were considered, especially when the original finding was too general or the illustration provided additional information.

#### Synthesized finding 1: Parents receiving communication on a child’s higher weight experience strong feelings that can affect their parenting

There were several reasons why communication regarding a child’s weight aroused negative feelings and reactions in parents, such as the HCP’s behavior, surprise at the child’s weight diagnosis, the parent’s own vulnerability, and the parent’s desire to protect the child. These strong emotions diverted parents from addressing their child’s weight, causing them to focus on their own emotions or defend their parenting practices. This synthesized finding is composed of 3 categories derived from 44 study findings (28 unequivocal, 16 credible; Table 2).

**Table 2:** Synthesized finding 1

Findings	Categories	Synthesized finding
En annen mor mente helsevesenet betraktet henne på en arrogant måte. [Another mother thought the healthcare system regarded her in an arrogant way.] (U)	Strong emotional reaction.	Parents receiving communication on a child’s higher weight experience strong feelings that can affect their parenting.
They found it difficult to understand why their child’s BMI was greater than normal when the nurse had confirmed that they were doing everything right. (C)		
Sensitive delivery of message. (U)		
Loneliness and vulnerability. (U)		
There was a sense among some that the programme had overstepped its role. (U)		
The majority of mothers who saw a different MCH nurse for either their 2‐year or 3‐and‐a‐half‐year visit, the experience was very different, and many perceived the consultation as damaging. (C)		
Frustrations experienced by families. (C)		
This underscores the importance of following up the weight feedback to try and provide the opportunity for parents/guardians to vent their anger. (U)		
Negative emotional reactions. (U)		
Different feelings about receiving the letter. (U)		
Parents being vulnerable. (U)		
Do not use scare tactics. (U)		
Shock that their child had been identified as overweight or obese. (U)		
Weight stigma and discrimination. (C)		
Mothers with no awareness of the child being overweight reported that they were either in shock, which disabled their listening skills so they could no longer absorb any information the MCH nurse gave them, or they reported feeling inadequate as a mother and that they had let their child down. (C)	Questioning the parenting.	
Frustration, guilt and lack of power. (U)		
In contrast, other parents said they experienced the weight conversation repeatedly, which led them to feel accused and attacked every time they visited the CHC. (U)		
Another parent described the weight conversation as making her feel both helplessness and defensive. (U)		
Several parents left the CHC visits feeling failure and regret, burdened by the sense they had made mistakes and feeling powerless to change the situation. (U)		
With some parents reporting a sense of being judged. (U)		
Belief that the judgement is unwarranted/Parental responsibility. (U)		
These mothers expressed feelings of blame, embarrassment, and shame when interacting with their children’s physicians. (C)		
Another mother said her child’s physician was ‘‘very, you know, nice about it’’ when talking about her child’s weight, but that the conversations made her… (C)		
Feeling unheard and questioned by physicians, particularly with respect to children’s diets, was a common experience by mothers. (U)		
Some parents described a hostile approach that left them feeling blamed, shamed, and isolated. (U)		
Communication. (C)		
Parents expressed mixed feelings about involving the child in the conversation on a healthy lifestyle, especially if weight was discussed. (U)	Fear of harming the child.	
Parents worried that focusing on weight might generate feelings of guilt or even increase the risk of the child developing eating disorders. (U)		
The same mother also described how the nurse spoke sensitively, in order to prevent the child from fully understanding what had been discussed. (U)		
Across the interviews, parents preferred for weight-related conversations to take place when the child was not present. (U)		
Belief that the judgement is unwarranted/Risk of harm. (U)		
Belief that the judgement is unwarranted/Lack of belief that weight is a risk to health. (C)		
One mother in particular felt that the MCH nurse was discriminatory towards her son. (C)		
Mothers suggested there are certain words the MCH nurse should avoid. (C)		
Raising the topic (of obesity). (C)		
Impact on child’s self-esteem. (C)		
Some parents/guardians (n = 3) chose to throw away the letter, determined not to let their children see it, as they feared that this could impact on their children’s self-esteem and mental wellbeing. (U)		
But some parents/guardians (n = 2) could not just let the feedback go without some response, so they took it upon themselves to write back to the authorities describing disgust about the letter they had received. (U)		
‘It’s a big worry to a 4-year-old’. (C)		
Relationship with the child. (U)		
Use discretion in weight discussions. (U)		
They were concerned the consultation would have a negative impact on their child’s mental well-being. (C)		
GP had caused offence in the way they had handled the consultation. (U)		
If participants had had negative experiences in the health system in relation to their weight or ethnicity, then they were less willing to engage. (C)		

U, unequivocal; C, credible.

BMI, body mass index; CHC, child health care; GP, general practitioner; MCH, maternal and child health.

##### Category 1.1: Strong emotional reaction

Parents described several different negative feelings and reactions when they received information on their child’s higher weight. The expressed feelings ranged from anger and bewilderment to surprise and defensiveness. These reactions caused the parent to become debilitated in terms of how to proceed with the child’s weight issue as a parent. Instead of looking for solutions, they were focused on the emotions triggered by the communication.

*Of course, it’s difficult. Nobody wants to hear there’s something wrong with their child. There is almost nothing that can make parents more terribly unhappy, hurt and angry like that. And you would like to defend… Still it’s so important how it’s presented to you. If I was to say to someone that “Now your kid is too fat – there is too much candy, too much fatty food and too much sitting in front of the TV”, then I would for sure not accomplish anything, right?^73^^(p.721)^*

*I was disgusted so I got in touch with the school nurse and expressed my anger.^69^^(p.149)^*

Parents who received a letter or referral informing them about their child’s higher weight experienced especially strong negative feelings. The referral sent home made the parents feel angry.

*How dare somebody tell me that my child is overweight…to be sent home with healthy eating leaflets, blah blah, you just think “actually?” I just felt it was a little bit too much.^57^^(p.2887)^*

Sometimes communication on the child’s weight made the parents act defensively or passively. Parents stated that the information they received aroused negative feelings and they wanted to avoid the information.

I didn’t want the comments to make me upset so I closed myself off to the health nurse and didn’t take anything on board.^60^^(p.3614)^

Overall, communication on the weight itself made parents feel uncomfortable. Parents perceived the child’s weight as a sensitive issue that was difficult to discuss neutrally.

*It’s kind of like a double-edged sword. You know, it was probably appropriate to send that out to us, but I didn’t want to receive it.^71^^(p.716)^*

In addition, parents felt vulnerable when communicating about the child’s weight. Parents often inferred the HCP’s attitude towards them based on their communication, as it evoked uncomfortable feelings.

*It was a very big difference. There [in the outpatient setting] I felt very lonely and almost as if I were attacked, even if that perhaps was not the case.^25^^(p.3)^*

The parents themselves also acknowledged this effect on their feelings and appreciated it if they had the opportunity to vent their feelings with the HCP.

*I am glad that I have had the opportunity to speak to you about it, because at the time I did feel very strongly about it, but maybe there were some other parents that felt as strongly but just didn’t do anything about it afterwards and just sort of went, ‘Oh well’.*^67^^(p.9)^

##### Category 1.2: Questioning the parenting

Several parents felt blamed or held responsible for the child’s weight due to the communication. Parents felt that their ability as parents was questioned, and they were seen as incapable parents.

*When the letter arrives it seems like a bolt out of the blue and that you are being heavily criticised as an irresponsible parent.*^58^^(p.990)^

*I felt people were telling me that I wasn’t bringing up Alana in the correct manner.*^69^^(p.149)^

Parents frequently perceived that HCPs were making assumptions about the nutritional quality of the food they provided for their children.

*It made me feel a little bit like I just feed my kids chips all the time, not a healthy balanced diet and I did feel a bit like I had had my fingers slapped.*^57^^(p.2887)^

Parents were uncertain about how to resolve the situation and determine the most appropriate and safest way to proceed. Some parents also expressed genuine uncertainty about the reasons behind their child’s weight gain and felt helpless as a result.

*Yes, you felt somehow powerless. My thoughts were: “what are we doing differently from others? or why does he keep gaining weight when other [children] do not?” What are we doing that the others are not doing, so you compare what you are doing. I do not give him more food or more sugar etc.*^25^^(p.3)^

These feelings of being accused and blamed made some parents feel that they are not good enough parents for their child.

*It just makes me feel like I’m less than a mom…Like I didn’t make the right choices for them and that’s why they’re like that.*^59^^(p.72)^

##### Category 1.3: Fear of harming the child

The parents’ willingness to protect their child and find the safest way to proceed in the situation was evident in their experiences. In addition to general uncertainty about how discussing weight would affect their child, parents had specific fears regarding their child’s well-being and sought ways to prevent these fears from being realized.

Parents hoped to arrange (or else arranged) the situation so that the child would not be exposed to weight feedback from the HCP. Either they did not show the weight letter to the child or tried to arrange the meeting in a way that the child could not hear the discussion. This was done to protect the child and his or her self-esteem, or because some parents had had unpleasant experiences related to weight in their past.

*… all along I thought my son was absolutely fine and then it had on the letter that he was very overweight and the certain illnesses that he could get when he is older which I was quite horrified about, and then I put it in the bin so he couldn’t see it cos I didn’t wanna worry him. Obviously he is old enough to read. You know what I mean?* 67 ^(p.478)^

*I didn’t want to take [daughter] to the doctor’s was because I was overweight as a child… I didn’t want her to get that, you know, like embarrassed and the way that I used to feel.* 76 ^(p.478)^

*Then I talked to her (GP) in the corner of the room, and told her that I thought talking about weight was difficult. We agreed on setting up a new appointment so that I could talk with her without him (the child) being present.* 73 ^(p.721)^

Often parents were afraid that communication on the child’s weight would harm the child’s self-esteem or body image. The parents did not want their children to think of their body as not acceptable or “right,” and some parents had fears that the communication could cause an eating disorder.

*…however, these questions at the end about BMI, there you might not talk to the child. At that age they shouldn’t think about themselves in that way…in the end it’s judging their body.*^47^^(p.5)^

*By telling children they are overweight you are causing eating disorders with Year 6 children where hormones are running riot and they are already very conscious of their body shapes.*^58^^(p.990)^

Avoiding the weight stigma was present in the parents’ experiences; they felt that concentrating only on weight would make their children feel embarrassed or inferior, and the parents did not want that.

*It’s a stigma, and stigma shouldn’t be placed on children.*^60^^(p.3613)^

#### Synthesized finding 2: A health care professional’s active and individual communication, with the sensitive use of words, creates a good communication experience for parents

The HCP’s competence and personality played a role in how parents experienced the communication regarding the child’s weight. Communication in studies was received from general practitioners, nurses, and other professionals, such as promotoras (community health workers, health promoters, or lay health workers) or dietitians. Parents valued sensitive and understanding encounters, and they were very conscious of what kind of vocabulary the HCP was using on weight. The conversation was easier when the family was already familiar with the HCP. This synthesized finding is composed of 3 categories derived from 60 study findings (47 unequivocal, 13 credible; Table 3).

**Table 3:** Synthesized finding 2

Findings	Categories	Synthesized finding
De aldri hadde fått noen kommentar fra helsesøster eller fastlege på barnets vekt. [They had never received any comment from the public health nurse or GP about the child’s weight.] (U)	(Pro)active and dialogical communication.	A health care professional’s active and individual communication, with the sensitive use of words, creates a good communication experience for parents.
Opplevde ikke å bli tatt med på råd eller bli spurt om egen kompetanse av helsepersonellet. [Did not feel included in decision-making or asked about my own expertise by the healthcare personnel.] (U)		
Provider-initiated discussion. (U)		
Lack of provider support. (U)		
Poor of continuity of care and lack of consistency of message. (U)		
Direct mail communication/timing. (U)		
Direct mail communication/What to expect during the visit. (U)		
Direct mail communication/List questions and concerns. (U)		
Health professionals rarely raise the child’s weight issue with the parents. (U)		
These parents said they felt listened to and that the conversation was a dialogue, with parents given the opportunity and time to ask questions. (U)		
When nurses kept the conversation at the child’s level and engaged with the child, this could be a positive experience. (U)		
Parents said that conversations where the nurse invited them to reflect on their child’s and family’s situation and needs were particularly constructive, with the nurse offering an empathetic ‘outsider’ voice. (U)		
Some parents felt their CHC nurse lacked enough knowledge to facilitate in-depth discussions about the child’s weight development. (U)		
A lack of commitment on the part of the nurse and felt that addressing the child’s weight was a process that they themselves started and pushed. (U)		
Bridging communication. (U)		
The lack of collaboration was challenged by parents who described experiences with clinicians who determined weight management goals and expectations for them in the absence of discussion. (U)		
Ongoing negotiation that included encouraging parents to voice their thoughts with clinicians serving as facilitators was identified by participants as favorable and critical to collaboration between partners. (U)		
Most mothers felt the MCH nurse did not ask them questions to determine the full details of the daily diet and exercise routines of their toddlers. (C)		
They also suggest offering support and discussing strategies for working together to identify solutions, rather than passing judgement. (C)		
These mothers explained they felt as though the relieving nurse was there to “tick‐the‐boxes” and was very direct with questioning, rather than trying to establish any rapport. (U)		
Letter is helpful, acceptable. (U)		
Could be helpful, motivating. (U)		
Inadequate response to weight concerns. (U)		
The general practitioner should play an active role not only in signalling the weight problem in time, but also in offering ongoing support. (U)		
Dialogue with health professionals. (U)		
Continually monitor child’s progress (not just weight). (U)		
Risk factors/consequences. (U)		
Communicate with parent. (U)		
Communicate with child. (U)		
Parents felt that involving families in decision-making and maintaining regular communication could help to sustain motivation. (U)		
A nonjudgemental, respectful, welcoming environment. (U)	Safe, empathic, and tailored communication.	
Cultural competency. (U)		
Local programs and resources. (C)		
Direct mail communication/Tone. (U)		
The emphasis was on developing activities that would be possible and realisable for a particular family. (U)		
Personal qualities. (U)		
Effective task performance. (C)		
The selection of language was tied to the family–professional relationship because parents described a willingness and commitment to engage in weight management when they perceived clinicians as being respectful and concerned with helping their family. (U)		
Accessibility and availability of clinical appointments were challenging for some parents who preferred less formal check-ins with clinicians, which could help to gauge their experiences to date and assess whether additional support was needed. (C)		
In nearly every interview, mothers commented that the MCH nurse should take a more holistic approach and gather more information on the family before providing BMI results and making recommendations. (C)		
The majority of mothers felt it was to offer evidence‐based information and support when discussing children’s weight, while not being judgemental. (C)		
The participants raised the importance of continuity of care and in particular, the interpersonal relationship aspect. (U)		
Sustaining improvements. (U)		
Fear of judgement and reassurance. (C)		
They felt the PCPs knew their child’s growth patterns, activity levels, and general eating habits, and these types of reactions were reassuring to them. (C)		
Provide encouragement. (U)		
Direct and empathic communication styles are most effective. (C)		
Compassionate and respectful care. (U)		
Direct mail communication/Language. (U)	Sensitive use of words and language.	
Parents identified the CHC nurses’ sensitive and validating language as an important part of conveying a non-judgmental attitude. (U)		
Likewise, in cases where the child was present when the nurse initiated the weight-related conversation, the parents felt it was important that the nurse normalized the situation for the child, using neutral and non-alarming language. (U)		
Most parents described being shocked when CHC nurses used words like overweight or obesity to describe their child’s weight status. (C)		
The language chosen by clinicians to discuss weight had the potential to be viewed as offensive by many parents. (U)		
Conversely, while there were some exceptions, receipt of overweight feedback was generally reported in overwhelmingly negative terms. (U)		
Communicating with sensitivity. (U)		
Other parents described an indiscreet approach by clinicians. (C)		
Appropriate words used. (U)		
Inappropriate words used. (U)		
These parents/guardians reported that it had never occurred to them that anyone would regard their children as overweight/obese and felt that it added to the ‘insult’ to see words like ‘overweight’ and ‘obese’ in bold letters. (U)		
Discussing weight in nonlabeling language was preferred. (C)		

U, unequivocal; C, credible.

BMI, body mass index; CHC, child health care; GP, general practitioner; MCH, maternal and child health; PCP, primary care provider.

##### Category 2.1: (Pro)active and dialogical communication

Parents felt that HCPs were sometimes passive or dismissive when discussing or bringing up the child’s weight issues, or they communicated in an overly general manner. Some parents felt that it was the HCP’s duty to discuss the child’s weight and give concrete and helpful information.

*His doctor has mentioned it, but I am here because I don’t think they [providers] do enough…There’s so many positive ways that they could help and suggestions that they could give, and they just kind of tell you, ‘Your child is overweight’ and leave it at that.*^52^^(p.85)^

*I have been getting her weight checked at the GP clinic with a nurse…she [nurse] was plotting it all on a graph…didn’t say anything that she [daughter] is overweight but I could see that she is off the graph…I asked her advice [nurse’s], she didn’t have any advice…I asked GP and he said that the main thing is that she [daughter] needs to be active*^54^^(p.5)^

There were parents who were happy when informed of their child’s weight. Some parents even hoped that the HCP would communicate directly to the child about the weight and its consequences.

This is very positive, and I think everybody should get it and get screened… I’m just glad the school noticed and they’re out there to help the kids with their weight.^66^^(p.214)^

*As a responsibility as my child’s pediatrician, you should say to me and the kid, “Listen, you are not eating right. This is what is going to happen if you don’t eat right. Mom, you need to help her.”*^52^^(p.83)^

It was important for parents that the HCP tried to create an equal dialogue between the HCP and the whole family, not just the parents. A dialogue—rather than a lecture given by the HCP—was seen as a motivating factor.

*[I]t’s like they give us room to, “well, what do you think?” So you bounce back a little bit and you give your ideas on what’s worked.*^35^^(p.14)^

*I think it’s always a little bit frustrating to go into something like that and have somebody start firing solutions at you before they even know what the problems are.*^35^^(p.14)^

In addition, sufficient frequency and regularity of meetings were things that made parents feel the weight issue was treated adequately.

*If overweight had been an issue and you could see that things were going in the wrong direction, I would hope for more focus and closer follow-ups. Perhaps we could make a plan about what to do. And then we would have a shared responsibility to carry it out.*^73^^(p.722)^

One of the parents’ expectations for the communication situation was a more holistic approach, meaning that the whole family would be involved, not just the child. In addition, this should involve the HCP considering other issues in the family’s life, not just the child’s weight.

*Well, the suggestions that I would do would be to involve the whole family. For it to be taken as a holistic approach to the family – not just for the one child. Maybe if, you know, we [the parents] were offered a free swim as well, we would have went swimming a lot more as a group.*^77^^(p.251)^

Parents valued the opportunity to prepare in advance for the meeting, facilitating more dialogical communication.

*I think it’s good [what to expect section of letter]. So knowing that they [provider] already know that this is going to happen when you go, and you got to bring the paper back, and then maybe it also kind of reminds the doctor again, too, that you guys were going to discuss this.*^52^^(p.94)^

##### Category 2.2: Safe, empathic, and tailored communication

When discussing the child’s weight, the most important characteristic for HCPs in the parents’ experience was empathy, which helped to create a safe atmosphere. Parents stated that an empathic HCP is sensitive, reassuring, supportive, and understands the family’s situation. In addition, the HCP offers encouragement and gives positive feedback.

*Check in. It would be nice just so she could say, “Well if I do this, the doctor will be proud of me.” You need that coaching, “You’re doing a good job.” You need to hear that as parents.*^74^^(p.333)^

*There was no judgement or anything like that which you felt quite often with other medical people. They tended to be you know “what do you do”, “oh well do you think you should be doing this”, whereas there was none of that, it was more like you spoke to other people and you heard other people’s things and thought, yeah I could try that.*^51^^(p.5)^

If the HCP was familiar before, and knew the family, it helped to achieve a positive communication experience for the parents.

*Would want to see the same health nurse for all children, as it’s more about being comfortable with continuity.*^60^^(p.3615)^

Overall, the parents were satisfied with the encounter and the communication when the HCP showed interest in the child and the family, made an effort to understand the family’s situation and background, and showed that s/he cared for the family.

*The truth is I felt good. It means that she’s looking out for you. It feels good that she’s engaged with what’s happening to you, what’s going on in your life.*^56^^(p.732)^

*… he really cares about our child, so it’s a good thing for everyone to adopt.* 61 ^(p.146)^

Parents valued individualized interactions in which their family’s unique background was acknowledged. This appreciation was further enhanced if the HCP was already familiar with the family.

*He [exercise practitioner] was so inspirational. I mean they couldn’t wait to get home and start doing exercises. He always did it so he incorporated as I said into their everyday life so it made it you weren’t going out of your way to do anything it was all sort of general things that was achievable…like walking more from home because she walked to school quite a lot but not walked home so things like that… he gave her like little charts as well so she could tick it off and stickers and I mean little girls love all things like that.*^53^^(p.106U)^

One parent also raised the importance of cultural competence, based on a language barrier.

*When my child has an appointment, most of the time my husband goes with him [my son], because my son’s pediatrician… does not speak Spanish and I don’t speak enough English [but my husband does speak English].*^52^^(p.84)^

##### Category 2.3: Sensitiveuse of words and language

The parents were not indifferent to the words the HCP was using, nor the way words were being used. Parents experienced strong negative feelings when the HCP used words related to weight that offended the parents. There were some differences in the parents’ experiences of the appropriate words to use, but most often the word *obese* raised negative reactions.

*The word that I don’t like – it was used for me, but also for my kid – obese. I don’t like that word, I really don’t like that word.*^66^*^(p.214)^*

*I like that you don’t use the word “obese”. You used “very overweight” because nobody wants to see that [their child is ‘obese’].*^52^^(p.93)^

In addition to certain words, some parents felt that the tone of the communication was indiscreet, hostile, or discriminatory. The parents were offended especially if this happened in front of the child.

*If you talk weight then I do not think, never, that the children should hear it … it is so emotionally charged for the adults and then it becomes emotionally charged for the children and it automatically becomes something negative.*^55^^(p.7)^

When the HCP was able to communicate neutrally, the parents were not as easily upset with the language. There were also expectations from parents that both parents and children should be confronted honestly and directly with weight issues.

*They just said my daughter was gaining X amount of weight… They didn’t say anything negative. They just said that they took her weight from previous years and then added it up to the next physical year.*^66^^(p.214)^

#### Synthesized finding 3: Parents want to receive information about the child’s higher weight that is useful to them and is based on an acceptable weight estimation

Parents wanted to receive information from HCPs to aid the family in making healthier everyday choices and to provide advice on managing their child’s weight safely. Parents did not value the information received if it was perceived as superficial or it was not possible to apply the advice to the family’s everyday life. The parents did not find the encounter useful when they felt the information on the child’s weight or the diagnosis of weight was not credible. This synthesized finding is composed of 2 categories derived from 43 study findings (36 unequivocal, 7 credible; Table 4).

**Table 4:** Synthesized finding 3

Findings	Categories	Synthesized finding
De ikke hadde så dårlig kosthold fra fø, deror krevde deltakselsen i prosjektet ikke noen stor endring. [They did not have that bad diet from before, therefore participation in the project did not require any major changes.] (C)	Information for understanding and managing higher weight.	Parents want to receive information about the child’s higher weight that is useful to them and is based on an acceptable weight estimation.
Step-by-step instructions (concrete suggestions). (U)		
Direct mail communication/Explanation of risks. (U)		
The advice was not practical enough. (U)		
The advice was either impractical or overambitious in their particular circumstances. (U)		
Conversations about the child’s weight issue with health professionals are often considered to be ineffective. (U)		
The use of the child’s weight chart in the conversation helped to dedramatize but at the same time capture the seriousness of the conversation. (U)		
Provided information they already knew, feeling, consequently, that they were not being listened to and that the nurse did not understand the family’s needs and requests for support. (U)		
They did not get enough information on how to tackle the problem at home. (C)		
No clearly written weight management strategies or care plans were provided. This lack of detail and direction caused frustration among mothers, as they did not know how to manage the situation. (C)		
Parents were well informed about the health implications of excess weight in children. (U)		
Parents were most interested in dietary management, meal planning, and nutritional guidance. (U)		
Parents engaged in weight management with their pediatrician were happy with the suggestions and working to incorporate them into their behavior. (U)		
Provides no new information. (U)		
Helpful feedback and support. (U)		
Addressed medical causes, consequences. (U)		
Inconsistent advice. (U)		
Practical and individualized advice. (U)		
Programme not needed. (U)		
Discuss weight-related health risks. (U)		
Weight-status improvement plan. (U)		
Advice/weight. (C)		
In contrast, monitoring of a child’s height and weight was described as giving the child a ‘complex’ since it was done in the absence of any advice, and it was apparent that lifestyle advice could be dismissed as unnecessary. (C)		
The relevance of measuring BMI in young children was questioned, as was the usefulness of labelling young children as having overweight. (U)	Disbelief or incomprehension of the weight criteria.	
At the same time, parents expressed a general mistrust of the concept of BMI and recalled being surprised and upset when the CHS measured a child’s BMI, either during their own visit or when this was reported by other parents. (U)		
Direct mail communication/BMI description & graph. (U)		
Lack of belief in judgement/Lifestyle not included in assessment. (U)		
Lack of belief in judgement/Child is naturally large. (U)		
Lack of belief in judgement/Puberty. (U)		
Lack of belief in judgement/BMI is not a valid measure. (U)		
Lack of belief in judgement/Isolated measurement. (U)		
Lack of belief in judgement/Normal relative to peers. (C)		
Belief that the judgement is unwarranted/Physical activity and lifestyle mitigate risks. (U)		
Belief that the judgement is unwarranted/Child will naturally grow out of being overweight. (U)		
Ensuring accuracy. (U)		
Parents had mixed definitions of overweight and obesity and were confused about BMI. (U)		
Questionable validity of BMI. (U)		
Interpreting the child’s weight status. (U)		
‘I didn’t think my son was overweight’. (U)		
Approach too generic. (C)		
Broad definitions of healthy. (U)		
Their child might not fit into a set of assessment criteria, this did not necessarily equate to their child being unhealthy. (U)		
The age of the child involved in the service affected the degree to which families chose to engage, due to a perception that children were too young to have weight problems. (U)		

U, unequivocal; C, credible.

BMI, body mass index; CHS, child health service.

##### Category 3.1: Information for understanding and managing higher weight

Parents received information on the child’s weight status, the consequences for the child’s health, and lifestyle advice both through written and face-to-face communication. Parents appreciated the information given or sent by the HCP when they felt that the information added to their knowledge and opportunity to further act in the right way regarding their child’s weight.

*I don’t think I ever had any negative, any kind of feedback is good feedback… just telling what kinds of foods to eat, the portion size, how often to eat, what time not to eat at.*^66^^(p.214)^

Some parents felt that the information given in the referral notes and letters on the child’s weight status, as well as the explanations of what the information meant, were useful. In these experiences, parents emphasized that it is better to know the risks related to a higher weight, because then one knows that something must be done.

*The second paragraph really grabbed me, and it was relevant to my family because every one of those health issues, someone in my family has experienced that. So, that would make me very concerned that my child was susceptible.*^52^^(p.94)^

When the parents felt that the information received was something that could be transferred to the family’s own everyday life, the communication experience was accepted and acknowledged.

*Well, they always give good nutritious advice, like to take them to the park for more long walks, to get her to dance more.*^64^^(p.103)^

The parents’ ability to utilize the received information improved when they felt that the professionals were polite, positive, and provided information that increased their knowledge.

*I’ve had a good experience… they’ve been so polite with her… giving hope to her that she can lose weight and feel the way that she wants… for my granddaughter, that wasn’t only what we were eating, was the new medication that she was taking increased her appetite and she gained weight, a lot of weight.*^66^^(p.214)^

On the other hand, when the parents felt that the information received was not helpful, they were not satisfied with the encounter. Especially if the received information was superficial or already familiar, the communication experience was perceived as negative. Parents were frustrated if they received automatic, general advice without an effort to acknowledge the family’s existing habits or resources.

*He [exercise practitioner] does come up with some unrealistic things which I did tell him about. I said, “You can’t”, because I hadn’t long split up with my husband and that, and at the time, money was quite short, do you know what I mean, so he was suggesting things that were going to cost, like, six, seven quid a time. And I’m like, “I can’t do that.”*^53^^(p.107)^

Sometimes information lacked concreteness that could be transferred to the everyday life of the families. The parents needed clear steps or concrete instructions for exercise or cooking.

*…give us some sort of guideline to follow, that’s more than just a discussion, like a menu for a child his age.*^64^^(p.103)^

*Mother: Some menu plan suggestions I think would have been very helpful. Particularly enabling you to get into it much quicker and to think of some… so you can get a few ideas of how to plan things.*^53^^(p.107)^

Sometimes the parents highlighted that they did not receive anything new (ie, they already knew the things the HCP was telling them). These experiences were consistent whether the communication was given via letters or during a meeting.

*The only thing that conversation was really about was that he was not allowed to eat too much sugar and he had to be more active outdoors. Yeah, no shit! That was too basic. That, we have already managed to figure out.*^55^^(p.6)^

*The Change4Life leafiets didn’t really help us because all the things that it says not to do, we weren’t doing anyways… so that kind of was irrelevant to us*.^68^^(p.4)^

##### Category 3.2: Disbelief or incomprehension of the weight criteria

Parents experienced disbelief in the overweight or obesity criteria or the estimation of their child’s weight. Using BMI as an indicator for the child’s weight level was not always credible for parents. They felt that BMI did not tell the whole truth, especially if the child was very active. In addition, parents felt that the assessment of weight with BMI did not sufficiently consider the child’s individual characters or growth.

*BMI may not tell the whole story about your child’s weight… An athletic child with a lot of muscle may have a high BMI but not be overweight.*^66^^(p.212)^

In addition to BMI disbelief, parents did not always agree with the diagnosis of overweight or obesity. They felt that their child was like others and, especially if they felt that their child was active, the weight status received from the HCP could not be correct. Sometimes parents used other children that were bigger than their own child as a proof that their child was not overweight or obese.

*And I think ‘she’s not obese, she’s a normal 5-year-old and she keeps up with the other kids.’*^69^^(p.150)^

*I thought it was incorrect because if you look at my son he is tall, slender and very active.*^62^^(p.127)^

*Have you seen her swing off trees and all that? If she was that obese she wouldn’t be able to that.*^69^^(p.150)^

*There are much fatter children out there and my son isn’t that bad!*^58^^(p.990)^

The parents’ experiences also highlighted the fact that a single measurement does not tell the whole truth and that the child’s age should be better taken into account when making weight assessments. According to parents, greater consideration should be given to adolescence and growth in height when evaluating weight.

*If weighed every year and there was a pattern of being overweight then action should be taken. But I strongly feel that one weight during Year 6 should not lead to a child being labelled overweight.*^58^^(p.990)^

*My daughter is 11 years old and going through puberty when girls develop ‘‘puppy fat’’. She is healthy (eating wise) and active.*^58^^(p.990)^

Some parents acknowledged the higher weight assessment but did not perceive it as problematic or risky, believing that future height growth would rectify the situation.

*He is very short for his age and I feel he will even out as he grows.*^58^^(p.660)^

Some parents desired clearer explanations of the weight results or weight graphs.

*I have to read it a couple times before I actually knew how to understand the graph… Be more forward to it: Your son falls right here.*^66^^(p.214)^

## Discussion

The purpose of this qualitative systematic review was to synthesize evidence about parents’ and carers’ experiences of communications from HCPs regarding a child’s higher weight. The 33 included studies yielded 147 unequivocal and credible research findings. There were 3 main features that influenced parents’ experiences. The first synthesized finding comprised parents’ reactions and emotions towards communication, which led them to become defensive or focus on their heightened emotions. The second synthesized finding pertained to HCPs and encompassed aspects such as activity, empathy, sensitivity, competence, language, and resources. The third synthesized finding concerned the information given during the communication and its content. In summary, parents wanted the communication to be helpful, suitable, and credible, with the use of sensitive words. Most importantly, it should be safe for the child, supporting their development and self-esteem, and should not question the parent’s capability.

Communication on the child’s weight from the HCP to the parents can have a significant effect on the adherence to treatment, the child’s self-esteem, and the parents’ perceptions of their own parenting capabilities. Parents are the main contributors to the child’s healthy environment, and when considering the maintenance of weight loss, children and young people themselves feel that family support and dynamics are among the main factors that support their success.^82^ Therefore, to conduct effective, ethical, and suitable counseling related to the child’s weight, it is important to understand the parents’ reactions, feelings, and overall experiences of the HCP’s communication.

In general, the first synthesized finding revealed that parents experience strong feelings and reactions regarding communication about the child’s weight, which is in line with previous research.^20^ If communication elicits a strong negative emotional reaction in parents, it hinders or delays parental actions that could improve weight-related conditions. In addition, feelings are often related to the experience of parenthood: how am I performing as a parent; how do others see me as a parent; and how can I protect my child? This result is in line with previous research, where parents feel or fear that their child’s weight is a proof of parental incapability^25^,^30^,^31^,^59^ or that parents struggle to decide the safest way to proceed with the child’s weight issues.^76^,^83^ When parents sense that the child could be harmed from the communication, they have strong negative feelings and try to protect the child from hearing or seeing the weight-related issues. This fear of harming the child’s self-esteem or body image is in line with previous research.^55^,^66^

Parents who have experienced weight stigma or feel negatively about their own weight are more likely to avoid discussing weight-related issues,^84^ and this theme appeared in this review: parents did not want their children to experience the same negative experiences they themselves had. Based on these results, there should be more consideration of parental feelings in health care when communicating about the child’s higher weight. If communication is limited to medical facts, it does not sufficiently motivate all parents to manage the circumstances influencing their child’s higher weight. Parents require a supportive environment for expressing their emotions and acknowledgment of their parenting efforts.

Synthesized finding 2 emphasized the role of the HCP in the communication. According to the parents, the most desired characteristic in HCPs is their ability to show genuine interest in the family, which includes being encouraging, supportive, and sensitive—demonstrating empathy towards the parent, child, and the family’s situation. The need for an empathic encounter in weight communication is seen also in previous research on the child’s weight,^78^ and empathy is one of the core elements in motivational interviewing.^85^ In addition, parents felt that sometimes the HCP was not active enough in bringing up the weight issue. Parents expressed that they would have wanted more information, which was found also by Ames *et al.*^20^ The passive behavior of the HCP can be related to feeling unsure when discussing child’s weight, as they feel that they do not have enough knowledge or experience to do so.^28^,^86^ On the other hand, if the HCP’s choice of words is inappropriate in the parents’ opinion, the meeting might also fail. Sometimes parents were so upset with the choice of words that it influenced the whole communication situation. In this review, words such as *obesity, overweight*, and *BMI* aroused negative feelings among parents, and this is in line with other research related to language.^20^,^63^,^66^

The debate over the appropriate terminology for discussing childhood obesity is long-standing. Sometimes this discussion arises due to public campaigns, such as the United Kingdom’s 2009–2011 social marketing campaign, Change4Life,^87^ or national programs such as the National Child Measurement Programme (NCMP) in the UK,^88^ or the BMI screening in Massachusetts, USA.^89^ These examples, and this review, confirm that the words used to describe a child’s weight status create a sensitive and personal experience for every parent. With NCMP, parents felt that by using theoretically informed narrative messages, the information on a child’s weight was more acceptable and this indicated they could help reduce negative reactance.^90^ People working in health care should probe the parents’ preferred words. Additionally, as the results of this review revealed, more research and information on the use of words related to higher weight are needed.

There are also structural factors that can increase or decrease parents’ willingness to communicate about the child’s weight. If there is an opportunity to establish consistency in personnel who meet the family, it helps parents to receive this information from a familiar HCP. Hardy *et al.*^60^ also found that a familiar nurse helps the continuity of care. Parents feel that a strong relationship with HCPs built on trust is important, and although digital solutions are acceptable to some extent, they do not replace face-to-face communication.^91^ In addition, if the communication happens only once a year or even more rarely, it does not facilitate a discussion on weight; previous research shows that having more contacts with HCPs increases the amount of information on the child’s weight.^92^

The need to strengthen the competency of HCPs to handle children’s weight issues has been recognized in previous research^20^,^93^ and this review confirms this need. HCPs have limited power to influence the resources used or the content of education; therefore, they would benefit from increased possibilities to work more effectively and ethically on children’s weight issues. Furthermore, although various professionals work with parents, addressing weight-related issues should be considered a multidisciplinary task, rather than being the sole responsibility of medical professionals. Enhancing parenting skills and supporting family dynamics are essential components in assisting parents of children with higher weight.

Synthesized finding 3 demonstrated the challenges associated with communicating about a child’s higher weight with parents. While several parents reacted negatively to hearing about their child’s higher weight, there were also parents who wanted and appreciated the information they received, especially if the information was necessary and specifically requested by the parents

Parents feel frustrated when they receive information that does not meet their expectations, situation, or opportunities. Previous research shows that, overall, parents do not want to be educated about healthy lifestyle behaviors; rather, they want strategies and support to handle the frustrations of food, screen, and sleep parenting.^94^ In addition, in the childhood obesity context, parents feel they do not always lack knowledge, but instead lack practical strategies for incorporating a healthy lifestyle in their own everyday life.^95^ This delivery of information that lacks relevance to the client’s needs or situation has long been recognized in the research; for example, behavior change theories and models such as COM-B (capability [C], opportunity [O], and motivation [M] as 3 key factors capable of changing behavior [B]), the Behavior Change Wheel,^96^ and the Health Action Process Approach^97^ all emphasize the importance of other factors, such as motivation, capability, or self-efficacy. Information alone is often insufficient; efforts to better understand the parent are likely to enhance communication about weight-related issues.

Several parents in this review expressed divergent views on the validity of BMI as a measure for assessing a child’s weight and health. The critique of BMI in children has been recognized before,^47^ and has been discussed and evaluated for over 10 years.^98^ This critique has also been present in situations where the child’s weight-status information has been sent by letter^99^ or measured in a health care meeting.^47^ This observation increases the need to develop health criteria that focus more on well-being instead of weight. In addition, some HCPs feel that BMI is not sufficient to evaluate a child’s health and well-being, and want more tools for the consideration of non-clinical factors shaping health and weight^100^; for example, in 2000, pediatricians used weight and height charts to recognize excess weight, rather than BMI.^101^ In addition to accepting the methods used for weight evaluation, parents need to understand how to interpret and utilize the information received, while also considering the child’s comprehension.^33^

When reviewing these results concerning parents’ adherence to treatment, there are a few aspects to consider. Street *et al.*^102^ note that there are 7 paths of communication between the patient and HCP that can lead to better health: increased access to care, greater patient knowledge and shared understanding, higher quality medical decisions, enhanced therapeutic alliances, increased social support, patient agency and empowerment, and better management of emotions. This review also identified parents’ empowerment and emotional management as important factors, which should be taken into account when communicating about a child’s higher weight.

In this review’s results, parents expect to receive information through communication, but they have clear preferences regarding what kind of information is useful. Parents appreciate it when the communication offers information that fits the family’s needs or situation; however, if the received information is too superficial or something they already know, parents become frustrated. The information provided should enhance parents’ understanding of the implications of higher weight and offer strategies to manage factors influencing their child’s weight. Families and parents also differ in terms of their preferred communication style. While some parents prefer direct confrontation regarding their child’s weight issues, including discussions in the child’s presence, others desire a more sensitive approach, and some do not wish to discuss weight at all. Some parents conduct weight talk with their children when they are concerned for their child’s health, whereas others avoid it for the same reason, as they do not want their children to become weight-obsessed.^84^

The parents’ different approaches to weight talk were also evident in this review’s results. This controversy places pressure on HCPs’ performance, requiring a delicate balance between proactive engagement and maintaining sensitivity in their communication approach. The child’s age must also be considered. Since only 3 studies in this review involved parents of children under 5 years, differences in parental experiences based on the child’s age could not be distinguished.

This review offers an updated understanding and knowledge of parents’ and carers’ experiences of the HCP’s communication about a child’s higher weight. This knowledge is particularly important, as the weight stigma puts pressure on initiating communication concerning weight. In this review, parents brought up their concerns about the communication causing weight anxiety in the child; thus, finding an approved communication style creates a safe environment for weight discussions. However, parents also feared being accused of bad parenting.

Development of communication skills is important for HCPs. There is existing research that has addressed this, for example, by presenting a guide for communication curriculum development in HCPs for educators and curriculum planners.^103^ This review highlights the importance of prioritizing aspects that are meaningful to parents, such as safeguarding the child and improving parenting strategies, when addressing communication about the child’s weight. Parents are more receptive to weight-related facts and information when they perceive the communication environment as non-confrontational. To prevent communication challenges for HCPs, it is essential to provide training opportunities, enhance communication skills, and ensure adequate resources for working with families.

### Strengths and limitations of the review

This review has several strengths:

- The review is based on a large number of eligible studies (n = 33) and, consequently, a large number of unequivocal and credible research findings (n = 147). This allows for a broad view of the experiences of parents and carers.
- The included studies originated in different countries and involved various ethnicities and cultures.
- The review team members have different experiences both in weight communication and conducting a systematic review.
- This review considered findings in the framework of the new obesity discourse and can assist HCPs’ development of the weight discussion.
- Focusing only on the research that included perceptions and experiences of parents with a child with a higher weight is valuable. Having a personal experience with a child’s higher weight affects parents’ perceptions of communication.
- This review included both face-to-face and written communication, which are both practices used in health care when informing parents on their child’s weight.

This review also has some limitations, which should be acknowledged when considering the veracity of the synthesized findings. The main limitation is related to the critical appraisal of the studies. The quality of studies varied and, as there were no cutoff scores for inclusion, low-quality studies were included in the review; however, there was only 1 study appraised as low quality.^80^ On the sensitive issue of weight communication, every parental experience was evaluated as useful, and thus, this limitation of the study was considered acceptable. In addition, the critical appraisal revealed some criteria were absent in several studies, such as Q1, Q6, and Q7 (see Table 1).

## Conclusions

This review confirms previous research that weight communication is a sensitive issue for parents. Parents face strong feelings when exposed to talk concerning their child’s higher weight. It easily arouses fear, feelings of being accused of being a failed parent, and the desire to protect the child from potentially harmful communication. Parents are ready to communicate when they are sure that the HCP is on their side and the child will not be harmed. In addition, information received should be suitable for the family’s situation, needs, and values.

The findings of this review emphasize the importance of addressing children’s higher weight with parents from perspectives beyond just medical considerations. In addition to sharing lifestyle or health information related to the child’s weight, HCPs should enhance their skills in creating a trustworthy and supportive communication environment. If parents perceive the meeting as successful, they are less likely to react defensively, allowing them to collaboratively consider solutions to improve the family’s situation. In terms of the development of weight communication, it is essential to invest in strengthening the expertise of HCPs, sufficient resources, and expanding multiprofessional cooperation to support family well-being. The focus of communication should prioritize the family’s well-being over the child’s weight, progressing in accordance with the family’s terms.

With the results of this review, the understanding increased on how and why parents react differently when communicating with HCPs about their child’s higher weight. This knowledge can, in addition, increase HCP’s activity in weight communication when they receive more knowledge from the parents’ experiences. The weight stigma needs to be considered in every communication about weight, and this review helps to reduce the parental weight stigma (weight stigma by association) by providing information about how to enable the parents to feel safe and capable when discussing their child’s higher weight.

### Recommendations for practice and policy

Based on these review findings, these recommendations have been graded according to the JBI Grades for Recommendations.^104^ Some of the recommendations have been documented in previous research. As the synthesized findings within this review are graded as low, the following recommendations are assigned Grade B.

#### Recommendations for practice

i) It is recommended that HCPs who communicate with parents and carers on a child’s higher weight have the requisite knowledge of sensitive communication and weight stigma. (Grade B)

ii) Shifting the communication focus from the child’s weight to overall well-being could encourage increased parental engagement in communication and treatment. (Grade B)

iii) The provision of care should be multidisciplinary and individualized according to the child’s, parents’/carers’, and family’s needs. (Grade B)

iv) Information that is provided to the parents and carers should be individualized to enhance that parent’s or carer’s understanding. (Grade B)

v) Contact with a familiar HCP was seen positively by parents and carers; therefore, consistent provision of HCPs is suggested. (Grade B)

#### Recommendations for policy

i) To enhance HCPs’ communication with parents and carers, it is essential to provide training opportunities on communication skills and to provide adequate resources for working with families. (Grade B)

### Recommendations for research

i) Fathers were under-represented in the study findings. Even though there is some research on childhood obesity from the father’s perspective,^105^ we need more research concentrating on fathers’ perspectives and experiences.

ii) Although this review included studies with different ethnic groups, ethnic diversity was not addressed sufficiently. We need more research on how different ethnicities relate to the new obesity discourse and how it affects weight communication.

iii) There are contextual differences in the prevalence of childhood obesity, such as a rural or urban environment^106^ and household income.^107^ Additionally, there are cultural differences attached to childhood obesity and its treatment.^108^ Therefore, it would be beneficial to do more research on the experiences of families from different backgrounds.

## Appendix I: Search strategy

### CINAHL (EBSCOhost)

Search conducted: July 22, 2022, and updated October 2, 2023

**Table:**

Search #	Terms	Records retrieved
1	(MH “Pediatric Obesity”)	16,532
2	(MH “Obesity”) AND ((MH “Child”) OR (MH “Infant”) OR (MH “Child, Preschool”))	13,134
3	TI ((obesity OR obese OR overweight) AND (child or children or childhood or pediatric or paediatric)) OR AB ((obesity OR obese OR overweight) AND (child or children or childhood or pediatric or paediatric))	26,155
4	S1 OR S2 OR S3	37,211
5	MH (communication)	90,692
6	TI (communicat* OR conversation* OR discuss* OR letter* OR feedback OR telephone counsel* OR dialog*) OR AB communicat* OR conversation* OR discuss *OR dialog* OR letter* OR feedback OR telephone counsel* OR dialog*)	294,573
7	S5 OR S6	339,911
8	MH parents OR MH caregivers OR MH mothers OR MH fathers	124,969
9	TI (parent* OR caregiver* OR carer* OR mother* OR father*) OR AB (parent* OR caregiver* OR mother* OR father*)	298,010
10	S8 OR S9	332,387
11	S7 AND S10	30,343
12	MH qualitative studies OR MH phenomenological research OR MH Ethnographic research OR Grounded theory or MH interviews OR Semi-Structured Interview OR Thematic analysis OR MH focus groups OR MH narratives OR MH action research	346,832
13	TI (focus group* OR qualitative OR descriptive OR ethnograph* OR fieldwork OR field work OR key informant* OR grounded theory OR phenomenolo* OR metasynthes* OR meta synthes* OR meta-synthes* OR action research OR mixed method* OR thematic analys*) OR AB (focus group* OR qualitative OR descriptive OR ethnograph* OR fieldwork OR field work OR key informant* OR grounded theory OR phenomenolo* OR metasynthes* OR meta synthes* OR meta-synthes* OR action research OR mixed method* OR thematic analys*)	324,483
14	TI (semi structured OR semistructured OR unstructured OR informal OR indepth OR in-depth OR “face to face”) OR AB (semi structured OR semistructured OR unstructured OR informal OR indepth OR in-depth OR “face to face”)	143,814
15	S12 OR S13 OR S14	549,457
16	S4 AND S11 AND S15	209
17	Limited 2010 – 2022	182

### MEDLINE (EBSCOhost)

Search conducted: July 22, 2022, and updated October 2, 2023

**Table:**

Search #	Terms	Records retrieved
1	(MH “Pediatric Obesity”)	12,358
2	(MH “Obesity”) AND ((MH “Child”) OR (MH “Infant”) OR (MH “Child, Preschool”))	28,156
3	TI ((obesity OR obese OR overweight) AND (child or children or childhood or pediatric or paediatric)) OR AB ((obesity OR obese OR overweight) AND (child or children or childhood or pediatric or paediatric))	51,942
4	S1 OR S2 OR S3	64,388
5	MH (communication)	95,087
6	TI (communicat* OR conversation* OR discuss* OR letter* OR feedback OR telephone counsel* OR dialog*) OR AB communicat* OR conversation* OR discuss *OR dialog* OR letter* OR feedback OR telephone counsel* OR dialog*)	1,121,498
7	S5 OR S6	1,161,135
8	MH parents OR MH caregivers OR MH mothers OR MH fathers	173,113
9	TI (parent* OR caregiver* OR carer* OR mother* OR father*) OR AB (parent* OR caregiver* OR mother* OR father*)	740,452
10	S8 OR S9	774,971
11	S7 AND S10	45,426
12	MH qualitative research OR Grounded theory or MH interview OR MH focus groups OR MH narrative OR MH metasynthesis	138,389
13	TI (focus group* OR qualitative OR descriptive OR ethnograph* OR fieldwork OR field work OR key informant* OR grounded theory OR phenomenolo* OR metasynthes* OR meta synthes* OR meta-synthes* OR action research OR mixed method* OR thematic analys*) OR AB (focus group* OR qualitative OR descriptive OR ethnograph* OR fieldwork OR field work OR key informant* OR grounded theory OR phenomenolo* OR metasynthesis OR meta synthesis OR meta-synthesis OR action research OR mixed method* OR thematic analys*)	568,467
14	TI (semi structured OR semistructured OR unstructured OR informal OR indepth OR in-depth OR “face to face”) OR AB (semi structured OR semistructured OR unstructured OR informal OR indepth OR in-depth OR “face to face”)	240,449
15	S12 OR S13 OR S14	761,622
16	S4 AND S11 AND S15	247
17	Limitation 2010 - 2022	232

### LILACS (https://lilacs.bvsalud.org/en/)

Search conducted: July 23, 2022, and updated October 2, 2023

(obes* OR overweight) AND (child* OR infant* OR pediatric OR paediatric) AND (communicat* OR conversation* OR discuss* OR letter* OR feedback* OR telephone counsel* OR dialog*) AND (parent* OR caregiver* OR carer* OR mother* OR father*) AND (db:("LILACS”) AND type_of_study:("qualitative_research”)) AND (year_cluster:[2010 TO 2022])

Records retrieved: 6

### PsycINFO (Ovid)

Search conducted: July 23, 2022, and updated October 2, 2023

**Table:**

Search	Terms	Records retrieved
1	(obesity and (child* or infant*)).hw,sh.	2342
2	((obesity or obese or overweight) and (child or children or childhood or pediatric or paediatric)).ab,ti.	12,121
3	1 or 2	12,377
4	Communication/ or Written communication/	39,892
5	(communicat* or conversation* or discuss* or letter* or feedback or telephone counsel* or dialog*).ti,ab.	1,240,969
6	4 or 5	1,248,547
7	Parents/ or Caregivers/ or Mothers/ or Fathers/	124,415
8	(parent* or caregiver* or carer* or mother* or father*).ti,ab.	436,029
9	7 or 8	444,591
10	3 and 6 and 9	877
11	Qualitative Methods/ or Phenomenology/ or Ethnography/ or Grounded Theory/ or Interviews/ or Semi-Structured Interview/ or Thematic Analysis/ or Focus Group/ or Focus Group Interview/ or Narrative Analysis/ or Action Research/ or Mixed Methods Research/	58,788
12	(focus group* or qualitative or descriptive or ethnograph* or fieldwork or field work or key informant* or grounded theory or phenomenolo* or metasynthes* or meta synthes* or meta-synthes* or action research or mixed method* or thematic analys*).ti,ab.	372,862
13	(semi structured or semistructured or unstructured or informal or indepth or in-depth or “face to face”).ti,ab.	167,107
14	11 or 12 or 13	477,374
15	10 and 14	245
16	limit 15 to yr = "2010 -Current”	210

### Scopus

Search conducted: July 26, 2022, and updated October 2, 2023

(TITLE-ABS-KEY ((obesity OR obese OR overweight OR pediatric OR paediatric) AND (child* OR infant*)) AND TITLE-ABS-KEY (communicat* OR conversation* OR discuss* OR letter* OR feedback OR “telephone counsel*” OR dialog*) AND TITLE-ABS-KEY (parent* OR mother* OR father* OR caregiver* OR carer*) AND TITLE-ABS-KEY (“focus group*” OR qualitative OR descriptive OR ethnograph* OR fieldwork OR “field work” OR “key informant*” OR “grounded theory” OR phenomenolo* OR metasynthes* OR “meta synthes*” OR “meta-synthes*” OR “action research” OR “mixed method*” OR “thematic analys*” OR “semi structured” OR semistructured OR unstructured OR informal OR indepth OR “in-depth” OR “face to face”)) AND PUBYEAR > 2010 AND NOT INDEX (medline)

Records retrieved: 683

### MEDIC

Search conducted: July 23, 2022, and updated October 2, 2023

obesity obese overweight lihav* ylipaino* AND vanhem* isä isät äiti äidit huoltaj* parent* caregiver* mother* father* AND lapsi laste* laps* child* infant* 2010 - 2022

Records retrieved: 50

### ProQuest Dissertations and Theses (ProQuest)

Search conducted: July 26, 2022, and updated October 2, 2023

noft(((obesity OR obese OR overweight) AND (child OR children OR childhood OR pediatric OR paediatric))) AND noft((communicat* OR conversation* OR discuss* OR letter* OR feedback OR telephone counsel* OR dialog*)) AND noft((parent* OR caregiver* OR carer* OR mother* OR father*)) AND noft((focus group* OR qualitative OR descriptive OR ethnograph* OR fieldwork OR field work OR key informant* OR grounded theory OR phenomenolo* OR metasynthes* OR meta synthes* OR meta-synthes* OR action research OR mixed method* OR thematic analys* OR semi structured OR semistructured OR unstructured OR informal OR indepth OR in-depth OR “face to face”))

Records retrieved: 961

## Appendix II: Studies ineligible following full-text review

1. Alexander DS, Alfonso ML, Nazaruk D. Exploring childhood obesity perceptions among caregivers of African American children. J Afr Am Stud. 2018;22(4):345–56.

*Reason for exclusion:* Ineligible patient population

2. Allen H, Callender C, Thompson D. Promoting health equity: identifying parent and child reactions to a culturally-grounded obesity prevention program specifically designed for Black girls using community-engaged research. Children. 2023;10(3):417.

*Reason for exclusion:* Ineligible patient population

3. Ávila‐Ortiz MN, Castro‐Sánchez AE, Zambrano‐Moreno A. Mexican mothers’ perceptions of their child’s body weight. Health Soc Care Community. 2017;25(2):569–77.

*Reason for exclusion:* Ineligible focus

4. Bailey KE. An exploratory study of child obesity concerns among African-American children and parents. Vol. 71. ProQuest Dissertations Publishing; 2009.

*Reason for exclusion:* Ineligible focus

5. Ball GDC, Farnesi BC, Newton AS, Holt NL, Geller J, Sharma AM, *et al.* Join the conversation! The development and preliminary application of conversation cards in pediatric weight management. J Nutr Educ Behav. 2013;45(5):476–8.

*Reason for exclusion:* Ineligible study design

6. Battisti F, Battisti H. A father’s response to having a child who is overweight/obese. FASEB J. 2016;30(10):1096.

*Reason for exclusion:* Ineligible focus

7. Bentley F, Swift JA, Cook R, Redsell SA. “I would rather be told than not know” - a qualitative study exploring parental views on identifying the future risk of childhood overweight and obesity during infancy. BMC Public Health. 2017;17(1):684–684.

*Reason for exclusion:* Ineligible patient population

8. Berge JM, Trofholz A, Danner C, Brandenburg D, Pusalavidyasagar S, Loth K. Weight and health-focused conversations in racially/ethnically diverse households with and without a child with overweight/obesity. Stigma Health. 2023;8(2):139–49.

*Reason for exclusion:* Ineligible context

9. Bergström H, Sundblom E, Elinder LS, Norman Å, Nyberg G. Managing implementation of a parental support programme for obesity prevention in the school context: the importance of creating commitment in an overburdened work situation, a qualitative study. J Prim Prev. 2020;41(3):191–209.

*Reason for exclusion:* Ineligible patient population

10. Blanchette S, Lemoyne J, Trudeau F. Tackling childhood overweight: parental perceptions of stakeholders’ roles in a community-based intervention. Glob Pediatr Health. 2019;6:2333794X19833733-2333794X19833733.

*Reason for exclusion:* Ineligible focus

11. Brito FA, Zoellner JM, Hill J, You W, Alexander R, Hou X, *et al.* From bright bodies to i choose: using a CBPR approach to develop childhood obesity intervention materials for rural Virginia. SAGE Open. 2019;9(1):215824401983731.

*Reason for exclusion:* Ineligible focus

12. Canfell OJ, Littlewood R, Wright ORL, Walker JL. “We’d be really motivated to do something about it”: a qualitative study of parent and clinician attitudes towards predicting childhood obesity in practice. Health Promot J Austr. 2023;34(2):398–409.

*Reason for exclusion:* Ineligible patient population

13. Cheng ER, Moore C, Parks L, Taveras EM, Wiehe SE, Carroll AE. Human-centered designed communication tools for obesity prevention in early life. Prev Med Rep. 2023;35:102333.

*Reason for exclusion:* Ineligible patient population

14. Cox JS, Searle AJ, Hinton EC, Giri D, Shield JPH. Perceptions of non-successful families attending a weight-management clinic. Arch Dis Child. 2021;106(4):377–82.

*Reason for exclusion:* Ineligible patient population

15. Davidson K, Vidgen H, Denney‐Wilson E. Parental opinions about the responsibility for assessing children’s weight status – a survey of Rockhampton parents. Aust N Z J Public Health. 2019;43(5):436–42.

*Reason for exclusion:* Ineligible patient population

16. Edmunds L, Rennie K, King S, Mayhew H. Experiences of those taking part in the BeeZee Bodies family-based weight management intervention: a qualitative evaluation. Int J Child Health Nutr. 2014;3(4):163–9.

*Reason for exclusion:* Ineligible focus

17. Eli K, Howell K, Fisher PA, Nowicka P. “A little on the heavy side”: a qualitative analysis of parents’ and grandparents’ perceptions of preschoolers’ body weights. BMJ Open. 2014;4(12):e006609–e006609.

*Reason for exclusion:* Ineligible focus

18. Farnesi B, Perez A, Holt NL, Morrison KM, Gokiert R, Legault L, *et al.* Continued attendance for paediatric weight management: a multicentre, qualitative study of parents’ reasons and facilitators. Clin Obes. 2019;9(3):e12304.

*Reason for exclusion:* Ineligible patient population

19. Fjone AL. “Child and family responses to weight management programs: a qualitative investigation”. Loma Linda University Electronic Theses, Dissertations and Projects; 2011.

*Reason for exclusion:* Ineligible focus

20. Gillison FB, Grey EB, Baber F, Chater A, Atkinson L, Gahagan A. The systematic development of guidance for parents on talking to children of primary school age about weight. BMC Public Health. 2023;23(1):1–1704.

*Reason for exclusion:* Ineligible patient population

21. Gillison FB, Grey EB, McConnell HE, Sebire SJ. Using narrative messages to improve parents’ experience of learning that a child has overweight. Br J Child Health. 2021;1(5):220–30.

*Reason for exclusion:* Ineligible patient population

22. Grootens-Wiegers P, van den Eynde E, Halberstadt J, Seidell JC, Dedding C. The “Stages towards Completion Model”: what helps and hinders children with overweight or obesity and their parents to be guided towards, adhere to and complete a group lifestyle intervention. Int J Qual Stud Health Well-Being. 2020;15(1):1735093–1735093.

*Reason for exclusion:* Ineligible focus

23. Guerrero AD, Slusser WM, Barreto PM, Rosales NF, Kuo AA. Latina mothers’ perceptions of healthcare professional weight assessments of preschool-aged children. Matern Child Health J. 2011;15(8):1308–15.

*Reason for exclusion:* Ineligible focus

24. Gutzmer K. “So, you’re a lean guy”: care provider, parent, and child communication about weight, diet, and physical activity. ProQuest Dissertations Publishing; 2018.

*Reason for exclusion:* Ineligible patient population

25. Haugstvedt KT, Graff-Iversen S, Bechensteen B, Hallberg U. Parenting an overweight or obese child: a process of ambivalence. J Child Health Care. 2011;15(1):71-80.

*Reason for exclusion:* Ineligible focus

26. Hirschfeld‐Dicker L, Samuel RD, Tiram Vakrat E, Dubnov‐Raz G. Preferred weight‐related terminology by parents of children with obesity. Acta Paediatrica. 2019;108(4):712–7.

*Reason for exclusion:* Ineligible study design

27. Holt NL, Neely KC, Newton AS, Knight CJ, Rasquinha A, Ambler KA, *et al.* Families’ perceptions of and experiences related to a pediatric weight management intervention: a qualitative study. J Nutr Educ Behav. 2015;47(5):427-431.e1.

*Reason for exclusion:* Ineligible focus

28. Hurtado Choque GA, Rodriguez MR, Soltani D, Baltaci A, Nagao-Sato S, Alvarez de Davila S, *et al.* Mixed-methods evaluation of father participation in an adolescent obesity prevention program with multiple delivery methods. Health Promot Pract. 2023;15248399231177300.

*Reason for exclusion:* Ineligible patient population

29. Kennedy BM, Davison G, Fowler LA, Rodriguez-Guzman E, Collins ML, Baker A, *et al.* Perceptions of a pragmatic family-centered approach to childhood obesity treatment. Ochsner J. 2021;21(1):30–40.

*Reason for exclusion:* Ineligible patient population

30. Kim HO, Kim GN, Park E. Perception of childhood obesity in mothers of preschool children. Osong Public Health Rese Perspect. 2015;6(2):121–5.

*Reason for exclusion*: Ineligible patient population

31. Knierim SD, Rahm AK, Haemer M, Raghunath S, Martin C, Yang A, *et al.* Latino parents’ perceptions of weight terminology used in pediatric weight counseling. Acad Pediatr. 2015;15(2):210–7.

*Reason for exclusion:* Ineligible patient population

32. Lee RLT, Brown M, Leung C, Chen H, Louie L, Chen J, *et al.* Family carers’ experiences of participating in a weight management programme for overweight children and adolescents with intellectual disabilities: an exploratory study. J Adv Nurs. 2019;75(2):388–99.

*Reason for exclusion:* Ineligible focus

33. Leite DAA, Macedo LG de, Aliani ML, Silva NA, Romano MCC, Araújo A. [Treatment of obesity in adolescents by a multidisciplinary health team: experiences of family caregivers]. Rev Enferm Digit Cuid E Promoção Saúde. 2021;6. [Portuguese]

*Reason for exclusion:* Ineligible patient population

34. Lowenstein LM. Assessing barriers among primary care providers to counseling families about obesity. ProQuest Dissertations Publishing; 2011.

*Reason for exclusion*: Ineligible patient population

35. Ma X, Li W, Rukavina PB. Challenges encountered by parents from urban, lower social economic class in changing lifestyle behaviors of their children who are overweight or obese. BMC Pediatr. 2023;23(1):1–457.

*Reason for exclusion:* Ineligible patient population

36. Martinez SM, Rhee KE, Blanco E, Boutelle K. Latino mothers’ beliefs about child weight and family health. Public Health Nutr. 2017;20(6):1099–106.

*Reason for exclusion:* Ineligible patient population

37. McKee MD, Maher S, Deen D, Blank AE. Counseling to prevent obesity among preschool children: acceptability of a pilot urban primary care intervention. Ann Fam Med. 2010;8(3):249–55.

*Reason for exclusion:* Ineligible patient population

38. McPherson AC, Knibbe TJ, Oake M, Swift JA, Browne N, Ball GDC, *et al.* “Fat is really a four‐letter word”: exploring weight‐related communication best practices in children with and without disabilities and their caregivers. Child Care Health Dev. 2018;44(4):636–43.

*Reason for exclusion:* Ineligible patient population

39. McPherson AC, Swift JA, Peters M, Lyons J, Joy Knibbe T, Church P, *et al.* Communicating about obesity and weight-related topics with children with a physical disability and their families: spina bifida as an example. Disabil Rehabil. 2017;39(8):791–7.

*Reason for exclusion:* Ineligible patient population

40. Moore KG, Bailey JH. Parental perspectives of a childhood obesity intervention in Mississippi: a phenomenological study. Qual Rep. 2013;18(48):1–22.

*Reason for exclusion:* Ineligible focus

41. Mulgrew KW, Shaikh U, Nettiksimmons J. Comparison of parent satisfaction with care for childhood obesity delivered face-to-face and by telemedicine. Telemed E-Health. 2011;17(5):383–7.

*Reason for exclusion:* Ineligible study design

42. Newson L, Povey R, Casson A, Grogan S. The experiences and understandings of obesity: families’ decisions to attend a childhood obesity intervention. Psychol Health. 2013;28(11):1287–305.

*Reason for exclusion:* Ineligible focus

43. O’Kane C, Wallace A, Wilson L, Annis A, Ma DWL, Haines J. Family-based obesity prevention: perceptions of canadian parents of preschool-age children. Can J Diet Pract Res. 2018;79(1):13–7.

*Reason for exclusion:* Ineligible patient population

44. Perez A, Holt N, Gokiert R, Chanoine JP, Legault L, Morrison K, *et al.* Why don’t families initiate treatment? A qualitative multicentre study investigating parents’ reasons for declining paediatric weight management. Paediatr Child Health. 2015;20(4):179–84.

*Reason for exclusion:* Ineligible patient population

45. Perez AJ, Kebbe M, Holt NL, Gokiert R, Chanoine JP, Legault L, *et al.* Parent recommendations to enhance enrollment in multidisciplinary clinical care for pediatric weight management. J Pediatr. 2018;192:122–9.

*Reason for exclusion:* Ineligible focus

46. Perry RA, Daniels LA, Bell L, Magarey AM. Facilitators and barriers to the achievement of healthy lifestyle goals: qualitative findings from Australian parents enrolled in the PEACH child weight management program. J Nutr Educ Behav. 2017;49(1):43-52.e1.

*Reason for exclusion:* Ineligible focus

47. Pescud M, Pettigrew S, Wood L, Henley N. Insights and recommendations for recruitment and retention of low socio-economic parents with overweight children. Int J Soc Res Methodol. 2015;18(6):617–33.

*Reason for exclusion:* Ineligible focus

48. Pinard CA, Hart MH, Hodgkins Y, Serrano EL, McFerren MM, Estabrooks PA. Smart choices for healthy families: a pilot study for the treatment of childhood obesity in low-income families. Health Educ Behav. 2012;39(4):433–45.

*Reason for exclusion:* Ineligible focus

49. Rachmi CN, Hunter CL, Li M, Baur LA. Perceptions of overweight by primary carers (mothers/grandmothers) of under five and elementary school-aged children in Bandung, Indonesia: a qualitative study. Int J Behav Nutr Phys Act. 2017;14(1):101.

*Reason for exclusion:* Ineligible focus

50. Rombeek M, De Jesus S, Prapavessis H, Dempsey AA, Fraser D, Welisch E, *et al.* Improving remote lifestyle intervention studies in children: participant and caregiver feedback of the smart heart study. Patient Educ Couns. 2020;103(7):1326–34.

*Reason for exclusion:* Ineligible patient population

51. Salemonsen E, Holm AL, Øen KG. Struggling with overweight or obesity in children - fathers’ perceptions and experiences of contributing factors, role and responsibility. Int J Qual Stud Health Well-Being. 2022;17(1):2093912–2093912.

*Reason for exclusion:* Ineligible focus

52. Sallinen Gaffka BJ, Frank M, Hampl S, Santos M, Rhodes ET. Parents and pediatric weight management attrition: experiences and recommendations. Child Obes. 2013;9(5):409–17.

*Reason for exclusion:* Ineligible focus

53. Schmied EA, Chuang E, Madanat H, Moody J, Ibarra L, Ortiz K, *et al.* A qualitative examination of parent engagement in a family-based childhood obesity program. Health Promot Pract. 2018;19(6):905–14.

*Reason for exclusion:* Ineligible patient population

54. Schwartz MM. Parental perceptions of body mass index referrals and overweight school-age children: Planting the seeds of health. University of Nebraska Lincoln; 2010.

*Reason for exclusion:* Ineligible year (December 2009).

55. Sease K, Griffin S, Rolke L, Forrester J. Feedback following a family-focused pediatric weight management intervention: experiences from the new impact program. Pediatrics (Evanston). 2020;146(1_MeetingAbstract):391–2.

*Reason for exclusion:* Ineligible patient population

56. Sharifi M, Dryden EM, Horan CM, Price S, Marshall R, Hacker K, *et al.* Leveraging text messaging and mobile technology to support pediatric obesity-related behavior change: a qualitative study using parent focus groups and interviews. J Med Internet Res. 2013;15(12):e272–e272.

*Reason for exclusion:* Ineligible focus

57. Swinney FV. Caregivers’ perceptions about discussing their children’s weight in the dental setting. ProQuest Dissertations Publishing; 2014.

*Reason for exclusion:* Ineligible patient population

58. Söderlund LL, Malmsten J, Bendtsen P, Nilsen P. Applying motivational interviewing (MI) in counselling obese and overweight children and parents in Swedish child healthcare. Health Educ J. 2010;69(4):390–400.

*Reason for exclusion:* Ineligible study design

59. Thomson J, Percival T, Lilo LS, Smith M. What are the success factors for children with obesity having obesity discussions? A cross-sectional multiple methods study in an emergency setting. Emerg Nurse NZ. 2020;8–15.

*Reason for exclusion:* Ineligible focus

60. Twiddy M, Wilson I, Bryant M, Rudolf M. Lessons learned from a family-focused weight management intervention for obese and overweight children. Public Health Nutr. 2012;15(7):1310–7.

*Reason for exclusion:* Ineligible patient population

61. Uy MJA, Pereira MA, Berge JM, Loth KA. How should we approach and discuss children’s weight with parents? A qualitative analysis of recommendations from parents of preschool-aged children to physicians. Clin Pediatr. 2019;58(2):226–37.

*Reason for exclusion:* Ineligible patient population

62. Wild CEK, Egli V, Rawiri NT, Willing EJ, Hofman PL, Anderson YC. “It’s more personal if you can have that contact with a person”: Qualitative study of health information preferences of parents and caregivers of children with obesity in New Zealand. Health Soc Care Community. 2022;30(5):e3106–15.

*Reason for exclusion:* Ineligible focus

63. Ziser K, Decker S, Stuber F, Herschbach A, Giel KE, Zipfel S, *et al.* Barriers to behavior change in parents with overweight or obese children: a qualitative interview study. Front Psychol. 2021;12:631678–631678.

*Reason for exclusion:* Ineligible focus

## Appendix III: Characteristics of included studies

**Table:**

Study, Year, Country	Methodology, methods for data collection and analysis	Phenomena of interest	Setting/context	Participant characteristics and sample size	Research findings relevant to the review question
Akselbo,^48^ 2015 Norway	Qualitative method; in-depth interviews The results were analyzed using systematic text condensation	Experiences of mothers of overweight girls when meeting health personnel	The primary health service	6 mothers of obese girls (7–12 years)	Mothers felt that HCPs were quite passive in discussing the child’s weight, or the discussion was not a dialogue or offering anything new. Mothers also expressed feelings of guilt after HCP meetings.
			Mothers had participated in 2-year research		
Anderson *et al.,*^51^ 2021 New Zealand	A qualitative, focus-group study	Understand the views and experiences of caregivers in a multidisciplinary program for healthy lifestyle change, Whānau Pakari	Community-based, healthy lifestyle intervention program in a mixed urban–rural region of Aotearoa New Zealand	6 parents/caregivers (focus group); 4 parents, 2 grandparents; 4 New Zealand European, 2 Māori	Parents experienced having support from the Whānau Pakari team focused on the whole family. A nonjudgmental, respectful, and welcoming environment was appreciated by the caregivers. Parents felt that the focus was on healthy lifestyle rather than weight loss.
	Focus-group interviews, analyzed using a thematic analysis approach				
				Children 4.5–12 years, affected by obesity	
Åsberg *et al.,*^47^ 2023 Sweden	A qualitative inductive approach using semi-structured interviews Qualitative content analysis	Parents’ recalled experiences of the health dialogue in children with overweight	Child’s 4-year health visit at the child health centers in southern Sweden	14 parents of a child (4 years) with overweight (mothers n = 11, fathers n = 3)	The health dialogue performed by the nurse was experienced as valuable by the parents. Parents wanted the growth curves to be followed but did not value BMI. Parents had mixed feelings about child participation and found the concept of a healthy lifestyle natural but challenging. Discussing a healthy lifestyle was seen as one of the obligations of the Child Health Service and parents had high expectations of the nurse.
				Parents were born in Sweden (n = 12), Europe, or outside Europe	
Ayash,^52^ 2011 USA	A doctoral thesis	Parental perceptions of pediatricians’ communication about obesity-related topics and the utility of direct-mail communication	Families receiving care in 2 different clinics in Massachusetts	39 parents, 4 focus groups	Parents were relieved if the HCP initiated the conversation about their child’s weight. While parents expressed feeling defensive and guilty, they would like to have a sensitive delivery of the message. However, parents also experienced a lack of support and follow-up by the providers.
				Parents of children with a BMI above 85^th^ percentile	
	Qualitative, semi-structured focus-group interviews				
			Communication + mail	Mothers 95%, children’s mean age 6 (Cambridge) and 8.5 (Harvard)	
				Ethnicity: White, Black, Hispanic, and other	
	Thematic analysis				
Banks *et al.,*^53^ 2014 United Kingdom	A qualitative study	Families’ reasons for engaging or not engaging with child obesity services	Children attended childhood obesity service	32 families; 15 families whose children attended a UK-based childhood obesity service and 17 families whose children withdrew from treatment	Parents feel that children should be involved in the decision to attend an obesity service and practitioners should, as much as possible, tailor advice to the circumstances of each family.
	Semi-structured interviews, in the families’ homes				
	Analyzed thematically				
				Children aged 5–16 years identified by GP as obese (BMI ≥ 98th percentile based on UK growth reference data)	
Davidson and Vidgen,^54^ 2017 Australia	A qualitive study	Parental reasons for the enrollment in a program about child’s weight	Parents who had enrolled in a healthy lifestyle program	21 parents; 1 male, 20 female	Parents expressed that HCPs rarely raised the child’s weight issue and the conversations are often considered to be ineffective.
	Semi-structured, qualitative telephone interviews				
				Age of the children 5–12 years (mean age ~9 years), who were above the healthy weight range for their age	
	Data were themed and analyzed according to the theoretical constructs of the Theory of Planned Behaviour				
Ek *et al.,*^25^ 2020 Sweden	Qualitative	Parents’ challenges on child overweight diagnosis, experiences on group treatment	Parents who participated in the More and Less Study	36 parents; 67% mothers, 33% foreign background	Raising a child with obesity is an emotional burden for parents, including loneliness and vulnerability, frustration and guilt, and uncertainty and worry about hunger and satiety. Parents’ were interested in gaining knowledge and positive parenting skills.
	Semi-structured interview over the phone				
				Children on average 5.1 years (4.1–6.7), BMI standard deviation score 3.0 (1.8-4.7)	
	Thematic analysis with an inductive approach, analysis followed a realist approach				
Eli *et al.,*^55^ 2022 Sweden	Qualitative	Parents’ of preschoolers with overweight or obesity experiences of child’s weight conversations	Participants were recruited through a childhood obesity randomized controlled trial	17 parents; 12 mothers, 5 fathers, and 3 had a foreign origin	When receiving the overweight/obesity diagnosis, conversations both empowered and provoked resistance. The diagnosis also aroused fear of transferring weight anxiety. Parents hoped to have the family involved and help to manage surroundings. The support from HCP should include answers to questions of what and especially how.
	Telephone-based, semi-structured interviews				
	Analyzed using thematic analysis, with a realistic approach				
				Children were between 3 and 7 years old (mean age 4.8 years) with overweight (n = 7) or obesity (n = 10)	
Falbe *et al.,*^56^ 2017 USA	Qualitative (not mentioned directly)	Promotora effectiveness in Active and Healthy Families	Active and Healthy Families is a culturally tailored, family-based program for addressing obesity disparities in a predominantly immigrant Latino population	23 parents; 21 mothers, 2 fathers; 86% Mexican ancestry	Parents expressed that they valued the promotoras’ personable and patient communication style, and their kindness and caring. Promotoras’ ability to perform effective task performance, create a positive environment, motivate, and increase parents self-efficacy was appreciated by the parents.
	In-depth, semi-structured interviews by phone, a grounded theory analysis				
				Children 5–12 years	
Farnesi *et al.,*^35^ 2012 Canada	A qualitative study	Collaboration between clinicians and parents	All parents had either recently completed or were currently completing a 16-session, group-based intervention for parents of overweight 8 to 12-year-olds	16 parents; 12 female, 4 male	Parents (and clinicians) framed collaboration around 3 elements: a positive therapeutic relationship, negotiation of health care delivery, and regular monitoring and evaluation. Words used to invite parents to participate in discussions and follow-ups were expressed as important factors by parents.
				Ethnicity: Caucasian (n = 13), Latino (n = 1), Aboriginal (n = 1), mixed race (n = 1)	
	Semi-structured focus groups or individual interviews				
	Data analysis following a modified technique for thematic analysis described by Boyatzis				
				Children aged 8–12, BMI percentile of 99.0 (range = 97.8–99.7)	
Gainsbury and Dowling,^57^ 2018 United Kingdom	A critical realist perspective, a contextualist method	Parents’ experiences of written feedback from the NCMP	Parents who had recently received written weight feedback	18 parents in total; 15 female; 8 out of 18 parents with children defined as overweight (n = 5) or very overweight (n = 3)	Parents rejected the overweight feedback due to negative terms or feelings of being judged. In addition, some parents felt that the program had overstepped its role.
	Focus groups, thematic data analysis by Braun and Clarke				
				4 focus groups, including both parents and they were possible to identify	
				Children aged 4–5 or 10–11 years	
Gillison *et al.,*^58^ 2014 United Kingdom	A mixed methods design; primary data were qualitative	The factors behind parents’ negative reactions to receiving information that their child is overweight or vey overweight	Parents reacting to a letter through UK NCMP	45 parents receiving a letter informing them their child, aged 4–5 or 10–11 years, was overweight (n = 33) or very overweight (n = 12)	The parents’ primary reasons for their negative reactions to the letter were lack of trust in the measures used, lack of belief that being overweight is important for children’s health, and fear that discussing weight could cause an eating disorder for the child.
	Open, qualitative responses to a survey, a combination of conventional and summative content analysis				
				Parents were White British people living in rural or urban areas	
Gorlick *et al.,*^59^ 2021 USA	A qualitative, exploratory research	Parents’ experiences of weight stigma by association	Mothers who reported feeling concern about their child’s/children’s weight were invited by an advertisement or contacting previous study participants of child feeding	34 mothers; 55.9% non-Hispanic White, 26.5% non-Hispanic Black or African American, 11.8% Hispanic/Latina, 5.9% non-Hispanic mixed race	Mothers expressed negative feelings when interacting with their children’s physicians about their children’s weight. Negative comments and feeling unheard and questioned made the mothers feel blamed for the child’s weight.
	Semi-structured interviews				
	Constant comparative method				
				Children 5–16 years	
Hardy *et al.,*^60^ 2019 Australia	A qualitative study, a phenomenological methodology	Parents’ experiences when discussing child overweight issues with the nurse	Parents attending community child health centers in Victoria, Australia	10 mothers of children identified as overweight or obese	Parents felt that nurses often “brushed over” the weight topic, leaving parents unsure how to manage their child’s weight situation at home. In addition, parents had preferences on which words to use about weight and what kind of role the health care professionals should have in child’s weight issues.
	In-depth, semi-structured, face-to-face interviews; an inductive qualitative thematic approach				
				Children 2–5 years; Caucasian background	
			Snowball sampling		
Jones *et al.,*^61^ 2014 Australia	Qualitative, semi-structured interviews, 8 face to face, 4 telephone	Perceptions and experiences of treating childhood obesity	Families involved in the previous study and families that had concerns about childhood obesity	12 families; 8 females, 5 males, children 8–16 years (mean age ~10 years)	Childhood obesity was considered as a sensitive issue and parents preferred the GP to raise the topic. For parents, the GP’s dedication and support were major factors that they sought.
	Data were thematically analyzed				
Jorda,^62^ 2015 USA	Doctoral thesis	To discover the meaning of school BMI screening and referral to parents	School’s BMI screening and referral	20 parents; 2 fathers, 18 mothers	Suggested changes for school screening and referrals included subthemes: sensitivity, accuracy, privacy, and notification.
				Voluntary semi-structured interviews with parents who received referrals from the school nurse that their child’s BMI was higher than the 95th percentile	
	Mixed methods				
	Phenomenology as delineated by van Manen (1997)				
	Thematic analysis according to van Manen; holistic, selective, and detailed approaches				

				Children: 1st grade (n = 11), 3rd grade (n = 2), 6th grade (n = 6), 1 child grade not mentioned (mean age ~8 years)	
Laurent,^63^ 2014 USA	A qualitative exploration	Parental recognition of overweight/obesity of their pre-adolescent child	Participants were recruited through clinician referrals and study flyers in northeastern United States	17 parents, including 4 males, 1 grandparent	Parents felt that an HCP played an important role in the discovery process of child’s weight. Some parents described a hostile or indiscreet approach by clinicians.
	Interviews				
	Strauss and Corbin’s model of grounded theory			Ethnicity: White	
				Pre-adolescent children aged 9–14 years	
				All but 2 children were clinically obese and these 2 met the criteria for overweight	
Lupi *et al.,*^64^ 2014 USA	Qualitative, structured, in-person interviews, coded qualitatively, analyzed thematically	Parental perceptions of the roles of the families and the pediatrician in childhood weight management	Parents visiting a pediatric clinic	22 parents having a child with BMI above 85th percentile; parents in total n = 69	Even though most of the results were not possible to separate (parents with a child below or above 85^th^ percentile for BMI), some results were marked from the parents with a child above 85^th^ percentile for BMI. These were the importance of pediatrician feedback and concerns about parents’ own behavior.
				White (n = 41), Black (n = 22), Hispanic/Latino (n = 3), Asian (n = 1), other (n = 2)	
				All children aged 3–12 years (median 6 years)	
Morenz-Harbinger,^65^ 2013 USA	Doctoral thesis	Obesity in toddlers	The project was focused on children between the ages of 2 and 6 years who attended a children’s medical center	21 parent/child dyads	Parents wanted additional support from the center to achieve success in the program. In addition, parents wanted to be understood in terms of the situations they faced, to have their concerns listened to, and to be met at their individual level to achieve the success they sought.
	A qualitative, interpretive, a community-based participatory action research			1 focus group with 4 parents and 17 parents interviewed by phone	
	2 focus groups, observations, and semi-structured interviews (in-depth in-person or/and telephone)			Children aged 2–6 years (mean age 5)	
	Themes, units of meaning, categories			Relative weight greater than the 75th percentile for BMI	
				11 African American, 8 Caucasian, 2 other race	
Moyer *et al.,*^66^ 2014 USA	Qualitative research, semi-structured focus-group interviews, qualitative content analysis	Parents’ understanding of and responses to the Massachusetts BMI letter	BMI letter to parents who were enrolled in a community-based, family-centered pediatric weight-management program	29 parents/caregivers; 8 focus groups; 24 female, 5 male	Parents expressed that the letter did not provide new information or that the letter was acceptable and could be helpful. BMI as a measure of children’s excess weight was questioned by parents. As for the communication, parents wanted appropriate words used and helpful feedback and support.
				Hispanic/Latino = 17, Non-Hispanic/Latino = 9, did not respond = 3	
				Children aged 8–14 years, ≥ 95th BMI-for-age percentile, enrolled in program	
Nnyanzi *et al.,*^67^ 2016 United Kingdom	A mixed method study	The impact of the NCMP feedback letters on parents’ understanding and feelings about their child’s weight status	NCMP	8 parents (16 in total; 13 female)	NCMP feedback letter aroused different reactions in parents and was often a surprise for a parent. This is why these feelings should be considered better and parents should be offered an opportunity to vent them. In addition, if parents were advised to visit their GP to resolve their child’s weight issues, it was perceived as inappropriate, and caused controversy and anger.
	1-on-1 semi-structured interviews in homes/neutral locations			8 parents from the group whose child had been indicated to be overweight or obese and 8 whose child had been indicated to be of ideal weight-status class, aged 4–5 and 10–11 years	
	Thematic content analysis method				
Peña and Payne,^68^ 2022 United Kingdom	A qualitative approach, interpretative phenomenological analysis	The parental experiences of adopting healthy lifestyle advice for children with disabilities living with overweight and obesity	Families referred for dietetic weight-management advice within a local community setting	n = 8; 7 mothers, 1 carer (big sister); children aged 5–15 (mean age 9 years)	Parents valued it when the HCP was reassuring and if the advice received was practical and individualized. But not every parent felt that they received advice or support prior to dietetic referral when managing their child’s weight.
	Semi-structured interviews, face to face/telephone			Ethnicity not mentioned	
Povey *et al.,*^69^ 2020 United Kingdom	A qualitative, realist approach	A decision not to attend a family-based childhood obesity management program	Declining to participate in the obesity management program	10 families	Some parents felt that the communication during recruitment was poor, including judgmental and pushy elements. The reasons to decline were negative emotional reactions, program was not felt as needed, the approach was too generic, or the worry about negative psychological impact. The families that stopped attending had individual barriers or felt that it did not offer something that the family needed at the moment.
				Potential service users of a family-based childhood obesity program; no other information	
				Parents were contacted on the basis of NCMP, targeted to children aged 4–5 and 10–11.	
	Semi-structured interviews, inductive thematic analysis by Braun and Clark				
Schalkwijk *et al.,*^70^ 2015 Netherlands	Qualitative study	Parents’ expectations and experiences in lifestyle interventions and the influence of the social context and social factors on these interventions	Interviewees were participants in a lifestyle intervention program	24 parents of overweight or obese children; 17 mothers, 7 fathers, 18 children (mean age 10 years)	Parents expressed the need for the GP to discuss overweight, but in a non-offensive way. In addition the process of weight loss should be acknowledged by HCPs.
	Semi-structured interviews, thematic coding				
				Dutch, Moroccan, Surinamese, Turkish	
Schwartz,^71^ 2015 USA	Qualitative study	The perceptions of parents whose school-aged children received a BMI referral letter stating their child is overweight	Letter informing overweight, based on state-mandated health screening conducted by nursing students	21 parents; 20 female, 1 male	Parents had varied feelings about receiving the letter; positive, neutral, or negative. Although a wide range of reactions, majority of parents valued the information on child’s weight.
	Semi-structured interviews, analysis: Strauss and Corbin’s grounded theory			Children 2nd–5th grade (6–11 years)	
				BMI letter indicating a child was at or above the 95th percentile	
Syrad *et al.*,^72^ 2015 United Kingdom	Qualitative study	Parents’ perceptions of their child’s weight and health risk after receiving weight feedback	Parents were recruited within the NCMP in England in 2010–2011 as part of a larger study aiming to evaluate the impact of NCMP feedback	52 parents of overweight and obese children aged 4–5 and 10–11 years	Parents see health as a broader question than weight, hence they experienced NCMP feedback as less credible.
	Semi-structured interviews, interpretative thematic analysis, a constructionist way, focusing at a latent/interpretative level				
				Telephone (n = 9) or in the respondents’ homes (n = 41)	
				50% White, 50% non-White	
				Female 83%, male 17%	
				Child weight status: overweight 37%, obese 63%	
Toftemo *et al.,*^73^ 2013 Norway	Qualitative study	Parents’ views and experiences on HCPs’ identification of their child’s overweight	HCPs at 7 well-child clinics in the eastern rural part of Norway informed parents about the study and handed out written invitations	11 parents; 10 mothers, 1 father	Being told that their child is overweight aroused feelings and concerns among parents. Parents felt vulnerable and had concerns about the child’s welfare due to the weight discussion. Parents wanted HCPs to express themselves carefully, and to ask for parents’ opinion. Giving warnings should be avoided.
	In-depth interviews				
	Qualitative data were analyzed through systematic text condensation as described by Malterud				
				Ethnic Norwegian	
				Children aged 2.5–5.5 years, ISO-BMI 25–29 (n = 7), ≥ 30 (n = 3)	
Turer *et al.,*^74^ 2016 USA	Qualitative study	Parental perspectives on weight-management strategies for their school-aged children	Parents were recruited from primary-care clinics	19 parents; 17 mothers, 2 fathers	Parents want the HCPs to monitor weight and health risks using discretion, to provide specific guidance for lifestyle changes, and a concrete weight-status improvement plan and consistent follow-up.
	Semi-structured focus groups				
				Latino 44%, African American 33%, White 23%	
	Grounded theory, thematic coding, and the constant-comparison method were used simultaneously				
				Children 6–12 years old (mean age 8 years) with a measured BMI ≥ 85th percentile	
Turer *et al.,*^75^ 2016 USA	Mixed methods analysis	Parents’ perspectives regarding the most important thing that a provider can do to help an overweight child improve weight status	Parents were recruited from an academic primary-care clinic and administered a 32-question survey	219 parents	According to parents, they prefer weight-management strategies that prioritize evaluating weight-related problems, growth-chart review, and regular follow-up.
				Latino 60%, African American 34%, non-Latin White 1%, more than one race 5%	
	Qualitative analysis to open-responses that were analyzed using margin coding and grounded theory				
				Children 2–18 years old, of those 71% were 2–11 years	
				36% overweight, 42% obese, 22% severely obese	
Turner *et al.,*^76^ 2012 United Kingdom	Qualitative study	Parents’ views and experiences of primary care childhood obesity treatment	Parents were contacted via a hospital-based childhood obesity clinic, general practices, and MEND-groups in Bristol, England	15 parents of obese children aged 5–10 years; 14 female, 1 male; 14 White, 1 mixed race	Parents see primary care as a suitable setting to treat childhood obesity, if practitioners will address child’s weight in a non-judgmental sensitive manner and are able to treat childhood obesity effectively.
	In-depth interviews, analyzed thematically, using framework approach				
				12 at homes, 3 by telephone	

Visram *et al.,*^77^ 2013 United Kingdom	Qualitative methods, semi-structured interviews, analyzed using thematic framework analysis	To examine the experiences of the ‘Balance It!’ program	All families listed on the program database	20 families; participants of the program for children with overweight or obesity; feedback from 3 additional families was obtained (n = 23)	Parents expressed that ‘Balance It’ can increase healthy habits, and they appreciated the possibility to tailor the program to the family’s needs. More effort should be made to involve families in decision-making and keeping regular communication to help to sustain motivation.
				Children aged 4–16 years (<8 years n = 5, 9–12 years n = 7, >13 years n = 8)	
Wagner *et al.,*^78^ 2022 USA	Qualitative analysis	Language and communication preferences for discussing their child’s weight	Patients were from the University of Chicago Comer Children’s outpatient clinic	Mothers (n = 8), whose children were diagnosed with obesity (children 8–18 years)	Several important communication strategies: nonlabeling language, beneficial for all, places where best discussed, direct and empathic communication.
	Phone interviews, open-ended questions, qualitative analysis				
				Non-White, with 5 African American and 3 Hispanic	
Wild *et al.,*^79^ 2020 New Zealand	Qualitative study	The barriers to attendance and retention at varying levels of engagement in Whānau Pakari	Eligible participants were parents and/or caregivers of children and adolescents who had been referred to the service	71 parents, carers, grandparents; 5 children/adolescents (64 interviews with 76 total participants); female = 65	Social norms affected how parents experienced the need to seek care for their child’s weight. In addition, previous experiences of health care affects parents’ perceptions and engagement with services.
	In-depth interview-based study				
	Thematically analyzed				
				Māori = 32, New Zealand European = 75, Asian = 7, other European = 5	
				Children 4–16 years, obesity BMI > 98th percentile or overweight BMI ≥ 91st percentile	

BMI, body mass index; GP, general practitioner; HCP, health care professional; ISO-BMI, body mass index adjusted for age and sex; MEND, Mind, Exercise, Nutrition… Do it!; NCMP, National Child Measurement Programme.

## Appendix IV: Study findings and illustrations

**Table:**

Finding	Illustration
**1. Akselbo I, Ingebrigtsen O. [Mothers of overweight children - experiences and challenges]. 2015;5(4):453–63. [Norwegian]^48^**	
De aldri hadde fått noen kommentar fra helsesøster eller fastlege på barnets vekt. [They had never received any comment from the public health nurse or GP about the child’s weight.]	Jeg fikk aldri noen kommentar fra legen om at hun var overvektig [I never received any comment from the doctor that she was overweight.] p. 456 U
Opplevde ikke å bli tatt med på råd eller bli spurt om egen kompetanse av helsepersonellet. [Did not feel included in decision-making or asked about my own expertise by the healthcare personnel.]	De spurte ikke om hvilke følelser vi hadde, det har ikke vært spørsmål om hva vi ville. [They did not ask about our feelings, there have been no questions about what we wanted.] p. 458 U
En annen mor mente helsevesenet betraktet henne på en arrogant måte. [Another mother thought the healthcare system regarded her in an arrogant way.]	Det er veldig vanskelig å ta kontakt med helsestasjonen, for føler deg uglesett. Som om det eneste du gjør er å spise junkfood, og at du er lat. Det er det inntrykket du får. [It is very difficult to contact the health center because you feel judged. As if all you do is eat junk food and that you are lazy. That is the impression you get.] p. 459 U
Ved spørsmål om egne tanker om god oppfølging ønsket alle mødrene ndividuelle samtaler med helsepersonalet med foreldre og barn adskilt. [When asked about their own thoughts on good follow-up, all the mothers wanted individual conversations with the healthcare personnel, with parents and children separated.]	NS
De ikke hadde så dårlig kosthold fra fø, deror krevde deltakselsen i prosjektet ikke noen stor endring. [They did not have that bad diet from before, therefore participation in the project did not require any major changes.]	Vi har liksom ikke fått råd som jeg ikke har visst om fra før. [We haven’t really received any advice that I didn’t already know.] p. 459 C
**2. Anderson YC, Wild CEK, Hofman PL, Cave TL, Taiapa KJ, Domett T, *et al.* Participants’ and caregivers’ experiences of a multidisciplinary programme for healthy lifestyle change in Aotearoa/New Zealand: a qualitative, focus group study. BMJ Open. 2021;11(5):e043516.^51^**	
A nonjudgemental, respectful, welcoming environment	There was no judgement or anything like that which you felt quite often with other medical people. They tended to be you know ‘what do you do’, ‘oh well do you think you should be doing this’, whereas there was none of that, it was more like you spoke to other people and you heard other people’s things and thought, yeah I could try that. p. 5 U
**3. Åsberg M, Derwig M, Castor C. Parents’ recalled experiences of the child centred health dialogue in children with overweight: a qualitative study. BMC Health Serv Res. 2023;23(1):289.^47^**	
Parents expressed mixed feelings about involving the child in the conversation on a healthy lifestyle, especially if weight was discussed.	It might be good if the child is there, if you do it in the right way and have this with weight, the focus on weight I mean, that you do that in the right way so it does not become something dramatic, shameful, I do not think that is good. p. 4, Table 2, U
Parents worried that focusing on weight might generate feelings of guilt or even increase the risk of the child developing eating disorders.	…however, these questions at the end about BMI, there you might not talk to the child. At that age they shouldn’t think about themselves in that way…in the end it’s judging their body. p. 5 U
Parents recalled being suspicious and mistrustful of discussions about weight because they did not think their children had overweight.	NS
The relevance of measuring BMI in young children was questioned, as was the usefulness of labelling young children as having overweight.	To put a BMI growth chart on a child is a little bit too much, a child develops all the time, they shoot up in height, stop growing, and then all of a sudden your child is overweight, obese maybe and then you need to put your child on a diet? No, you should not put a child on a diet! The child should be allowed to follow the growth chart. p. 6 U
Parents did not recall nurses using the word overweight at the 4-year health visit but instead recalled that the nurse had said that there was nothing to be concerned about and that the child was of normal weight. They described that this made them feel that there was nothing to worry about.	NS
Despite their wish to focus on a healthy lifestyle rather than weight, parents said they recalled wanting to know whether their child had overweight and suggested the height and weight curve as a basis for these conversations.	NS
At the same time, parents expressed a general mistrust of the concept of BMI and recalled being surprised and upset when the CHS measured a child’s BMI, either during their own visit or when this was reported by other parents.	Considering what I have read over the last few years; yes, it is literature describing the weights of adults, BMI is not a very good valuation… it is not widely representative of weights because circumstances can be so different. p. 4, Table 2, U
They found it difficult to understand why their child’s BMI was greater than normal when the nurse had confirmed that they were doing everything right.	She and her sister have gained a lot of weight and we’re working on it, but you can’t make drastic changes with kids and you don’t want to put them on a diet. I feel we can hardly have candy on Saturdays or take a cake in the middle of the week and I think you should be able to do that… I grew up with a bad attitude towards my body, but I have managed to deal with it. p. 6 C
**4. Ayash CR. Clinic-based interventions to address childhood obesity: part of the solution to a public health problem? ProQuest Dissertations Publishing; 2011.^52^**	
Provider-initiated discussion	“As a responsibility as my child’s pediatrician, you should say to me and the kid, ‘Listen, you are not eating right. This is what is going to happen if you don’t eat right. Mom, you need to help her.’” p. 83 U
Sensitive delivery of message	“With other physicians or pediatricians who may mention this [concern with child’s weight] or who are overbearing, I think it is the delivery of the information, because I think it is only natural for a parent to be…in defense of their children especially if they have been picked on at school. So, I think that if the [provider] does not deliver the information correctly, then that sets up a barrier right off the bat.” p. 83 U
Cultural competency	“When my child has an appointment, most of the time my husband goes with him [my son], because my son’s pediatrician… does not speak Spanish and I don’t speak enough English [but my husband does speak English].” p. 84 U
Lack of provider support	“His doctor has mentioned it, but I am here because I don’t think they [providers] do enough…There’s so many positive ways that they could help and suggestions that they could give, and they just kind of tell you, ‘Your child is overweight’ and leave it at that.” p. 85 U
Poor of continuity of care and lack of consistency of message	“The doctor never tells me exactly that he is overweight, he only says to me ‘I think that he is healthy, is eating well, is growing well, is developing well, I see that he is active, when I talk to him I see that everything is ok’. And the nutritionist from WIC is the one that always told me that he is overweight, that I have to give him 2% milk and that I have to put water in the juice, and give him less [food].” p. 86 U
Step-by-step instructions (concrete suggestions)	“They always mention it [weight], and they mention if they are improving, if their BMI is reducing. That’s great and everything, but there is no detail about how you would get from A to B. But there really needs to be much more discussion about how to get from a 95% BMI to a 90% or lower.” p. 87 U
Local programs and resources	“I think if they offered alternatives, [but] I would be stressed out if you are telling that this is the thing I need to do, but then I have to go and battle with the insurance company. Give me an outlet, give me an alternative. So, I think that would be helpful.” p. 88 C
Direct mail communication/timing	“I wouldn’t want to wait a whole year to see if she does have these traces of high blood pressure or blood sugar, or whatever. I would want them to retest her again, at least every six months. Don’t wait a whole year. A lot does change within a year.” p. 93 U
Direct mail communication/Tone	“Yes, I like it [tone] because the physician is pretty much saying, ‘Let’s work together to get everything on the right track.’” p. 93 U
Direct mail communication/Explanation of risks	“The second paragraph really grabbed me, and it was relevant to my family because every one of those health issues, someone in my family has experienced that. So, that would make me very concerned that my child was susceptible.” p. 94 U
Direct mail communication/Language	“I like that you don’t use the word ‘obese’. You used ‘very overweight’ because nobody wants to see that [their child is ‘obese’].” p. 93 U
Direct mail communication/BMI description and graph	“That graph they have on the computer [growth chart] and it goes up and down, whatever, yeah, that’s clear to me. [The provider] is like, ‘This is where your child is, and this is where all these other kids are.’” p. 94 U
Direct mail communication/What to expect during the visit	“I think it’s good [what to expect section of letter]. So knowing that they [provider] already know that this is going to happen when you go, and you got to bring the paper back, and then maybe it also kind of reminds the doctor again, too, that you guys were going to discuss this.” p. 94 U
Direct mail communication/List questions and concerns	“Yes [it is good]. We have a lot of questions, but when we go to the pediatrician we have so many questions that we forget.” p. 94 U
**5. Banks J, Cramer H, Sharp DJ, Shield JP, Turner KM. Identifying families’ reasons for engaging or not engaging with childhood obesity services: a qualitative study. J Child Health Care. 2014;18(2):101–10.^53^**	
The emphasis was on developing activities that would be possible and realisable for a particular family.	He [exercise practitioner] was so inspirational. I mean they couldn’t wait to get home and start doing exercises. He always did it so he incorporated as I said into their everyday life so it made it you weren’t going out of your way to do anything it was all sort of general things that was achievable…like walking more from home because she walked to school quite a lot but not walked home so things like that… he gave her like little charts as well so she could tick it off and stickers and I mean little girls love all things like that. p. 106 U
The advice was not practical enough	Mother: Some menu plan suggestions I think would have been very helpful. Particularly enabling you to get into it much quicker and to think of some… so you can get a few ideas of how to plan things. p. 107 U
The advice was either impractical or overambitious in their particular circumstances.	He [exercise practitioner] does come up with some unrealistic things which I did tell him about. I said, ‘You can’t’, because I hadn’t long split up with my husband and that, and at the time, money was quite short, do you know what I mean, so he was suggesting things that were going to cost, like, six, seven quid a time. And I’m like, ‘I can’t do that’. p. 107 U
**6. Davidson K, Vidgen H. Why do parents enrol in a childhood obesity management program? A qualitative study with parents of overweight and obese children. BMC Public Health. 2017;17(1):159.^54^**	
Health professionals rarely raise the child’s weight issue with the parents	‘I have been getting her weight checked at the GP clinic with a nurse…she [nurse] was plotting it all on a graph…didn’t say anything that she [daughter] is overweight but I could see that she is off the graph…I asked her advice [nurse’s], she didn’t have any advice…I asked GP and he said that the main thing is that she [daughter] needs to be active’ p. 5 U
Conversations about the child’s weight issue with health professionals are often considered to be ineffective	‘I went to GP and dietitians for help but found that it was not enough, it was food based only…adult focused advice not child focused. They spoke to me more than to her. I regret taking her to the dietitian, she was nice but gave us all these charts and explained it all…she [daughter] at home later on said that she’s so fat according to these graphs and was upset about it’ p. 5 U
**7. Ek A, Nordin K, Nyström CD, Sandvik P, Eli K, Nowicka P. Responding positively to “children who like to eat”: parents’ experiences of skills-based treatment for childhood obesity. Appetite. 2020;145:104488.^25^**	
Loneliness and vulnerability.	It was a very big difference. There [in the outpatient setting] I felt very lonely and almost as if I were attacked even if that perhaps was not the case. p. 3 U
Frustration, guilt and lack of power	Yes, you felt somehow powerless. My thoughts were: ‘what are we doing differently from others? or why does he keep gaining weight when other [children] do not?’ What are we doing that the others are not doing, so you compare what you are doing. I do not give him more food or more sugar etc. p. 3 U
**8. Eli K, Neovius C, Nordin K, Brissman M, Ek A. Parents’ experiences following conversations about their young child’s weight in the primary health care setting: a study within the STOP project. BMC Public Health. 2022;22(1):1540.^55^**	
These parents said they felt listened to and that the conversation was a dialogue, with parents given the opportunity and time to ask questions.	“It [the conversation] was not judgmental, it was not a lecture, it was not stressful, but I was still taken seriously, and we talked about it [the weight] properly, but there were no pointed comments. I never felt like “oh my goodness, she thinks I’m the world’s worst parent because I might have an overweight child”, it was nothing like that.” p. 5 U
Parents identified the CHC nurses’ sensitive and validating language as an important part of conveying a non-judgmental attitude.	“She gave our daughter something to play with and told her that “we adults are going to talk a little”, I thought she told us in a very smooth manner but also very … in the serious situation, listen[ed] and asked if we were surprised over it [the daughter’s weight] and if there was something regarding her weight that we had thought about.” p. 5 U
Likewise, in cases where the child was present when the nurse initiated the weight-related conversation, the parents felt it was important that the nurse normalized the situation for the child, using neutral and non-alarming language.	“She gave our daughter something to play with and told her that “we adults are going to talk a little”, I thought she told us in a very smooth manner but also very … in the serious situation, listen[ed] and asked if we were surprised over it [the daughter’s weight] and if there was something regarding her weight that we had thought about.” P. 5 U
The same mother also described how the nurse spoke sensitively, in order to prevent the child from fully understanding what had been discussed.	“… she [the nurse] was aware not to talk about this in such a way that our daughter understood exactly what it was about, and she sort of suggested that we should take [the information home], to land [settle/take this information in], to talk a little at home and then have a meeting, a phone call together, … we could quickly agree on that.” p. 5–6 U
When nurses kept the conversation at the child’s level and engaged with the child, this could be a positive experience.	“She [the child] can give feedback on what we have done and what we have talked about, it has been very good actually, and I’ve never experienced that she [the child] felt … bad about it, but it has been at her level, and it has been just right and not pushed too much either.” p. 6 U
Parents said that conversations where the nurse invited them to reflect on their child’s and family’s situation and needs were particularly constructive, with the nurse offering an empathetic ‘outsider’ voice.	“I also remember that one of the first [conversations], like, when she had stated that ‘I see that a lot has happened in the last year’ and asked me ‘what are your reflections, … have you reflected on this [weight change] during this year?’, she kind of invited me to think and land [settle/take this information in].” p. 6 U
The use of the child’s weight chart in the conversation helped to dedramatize but at the same time capture the seriousness of the conversation.	“… [it became] very visual with this graph and that (the nurse said) “then you see it [the child weight development] very clearly here, but we will follow up …”. So, I think it got us to notice [the child’s increased weight status] in a very good professional way.” p. 6 U
Some parents felt their CHC nurse lacked enough knowledge to facilitate in-depth discussions about the child’s weight development.	One mother said the CHC nurse said her daughter was “two above the curve” on the weight chart but provided no further explanation. The mother had to ask, “What does it mean? Is it dangerous? Should I do something?” and said that “[i]f I had not asked such questions, she might not have said more than that she is two lines above the curve.” p. 6 U
Provided information they already knew, feeling, consequently, that they were not being listened to and that the nurse did not understand the family’s needs and requests for support.	“The only thing that conversation was really about was that he was not allowed to eat too much sugar and he had to be more active outdoors. Yeah, no shit! That was too basic. That, we have already managed to figure out” p. 6 U
A lack of commitment on the part of the nurse and felt that addressing the child’s weight was a process that they themselves started and pushed.	“… if we raise the question when we are at the CHC then she [the nurse] says. ‘Yes but try to think about eating a lot of vegetables’ … you see, there has been no more [guidance] than that.” p. 6 U
In contrast, other parents said they experienced the weight conversation repeatedly, which led them to feel accused and attacked every time they visited the CHC.	“If I can be blunt, it feels stupefying [the conversations at CHC]. You go in there and someone points with their finger “no, no” [the way you would say to a child], … that’s what it feels like … that you are a fool and a complete idiot that has let your child become fat …” p. 6 U
Another parent described the weight conversation as making her feel both helplessness and defensive.	“And then there was a nurse there … who simply said, ‘he is super overweight and obese, and this is diabetes and there is no hope’, like… . So, of course that’s not nice to hear and you get offended and then you become defensive because it’s your child and such, but above all, there were no broader hints [discussions] about how big he really was. Or why he’s so big. It was just blindly looking at this number, like.” p. 6-7 U
Across the interviews, parents preferred for weight-related conversations to take place when the child was not present.	“When he was drawing [during a visit to the CHC] … He was there busy drawing and then [he asked] ‘Dad, should I not do this? Did I do something wrong?’, Like something like that. And then when we got home; ‘But Dad, what is it with my stomach?’… So that, yes, really completely wrong!” p. 7 U
Several parents left the CHC visits feeling failure and regret, burdened by the sense they had made mistakes and feeling powerless to change the situation.	“… regarding the more general topics such as vaccines and how the breast-feeding works … then I have felt more involved … but this might be because there I haven’t had any problems if I compare to this weight discussion, where I feel it is a little rock that gnaws somewhere.” p. 7 U
Most parents described being shocked when CHC nurses used words like overweight or obesity to describe their child’s weight status.	“if you talk weight then I do not think, never, that the children should hear it … it is so emotionally charged for the adults and then it becomes emotionally charged for the children and it automatically becomes something negative.” p. 7 C
**9. Falbe J, Friedman LE, Sokal-Gutierrez K, Thompson HR, Tantoco NK, Madsen KA. “She gave me the confidence to open up”: bridging communication by promotoras in a childhood obesity intervention for Latino families. Health Educ Behav. 2017;44(5):728–37.^56^**	
Bridging communication	“I felt like family when she spoke. She spoke to our way of living. For me it was good. She instilled trust in us. She used simple and clear words so that we could be able to understand.” p. 732 U
Personal qualities	“The truth is I felt good. It means that she’s looking out for you. It feels good that she’s engaged with what’s happening to you, what’s going on in your life.” p. 732 U
Effective task performance	Well, at an individual appointment, they weigh him [and say], “Your son is doing okay. Thank you very much. The next appointment is on this date.” You see?… It’s more dry. There isn’t communication. [No] “How are you? How do you feel?” And in the group, it’s different because [the promotoras] say, “How are you? How did it go today?” There was more enthusiasm, more communication. p. 733 C
**10. Farnesi BC, Newton AS, Holt NL, Sharma AM, Ball GDC. Exploring collaboration between clinicians and parents to optimize pediatric weight management. Patient Educ Couns. 2012;87(1):10–17.^35^**	
The language chosen by clinicians to discuss weight had the potential to be viewed as offensive by many parents.	‘‘[H]e [pediatrician] just asked Sally ‘how’s it feel being fat’. […] well, I didn’t really care what he said after that’’ p. 13 U
The selection of language was tied to the family–professional relationship because parents described a willingness and commitment to engage in weight management when they perceived clinicians as being respectful and concerned with helping their family.	‘‘[I]t was an OK process because the way he [pediatrician] dealt with it [discussing weight] and talked to us was very professional and very kind about it’ p. 14 U
The lack of collaboration was challenged by parents who described experiences with clinicians who determined weight management goals and expectations for them in the absence of discussion.	‘‘I think it’s always a little bit frustrating to go into something like that and have somebody start firing solutions at you before they even know what the problems are’’ p. 14 U
Ongoing negotiation that included encouraging parents to voice their thoughts with clinicians serving as facilitators was identified by participants as favorable and critical to collaboration between partners.	‘[I]t’s like they give us room to, ‘well, what do you think?’ So you bounce back a little bit and you give your ideas on what’s worked’’ p. 14 U
Accessibility and availability of clinical appointments were challenging for some parents who preferred less formal check-ins with clinicians, which could help to gauge their experiences to date and assess whether additional support was needed.	‘‘I’m surprised about how encouraging they are. It’s like, I don’t think that was such a big achievement, but oh no no, rah rah! [clinician cheering on the family]’’ p. 14 C
**11. Gainsbury A, Dowling S. “A little bit offended and slightly patronised”: parents’ experiences of National Child Measurement Programme feedback. Public Health Nutr. 2018;21(15):2884–92.^57^**	
Conversely, while there were some exceptions, receipt of overweight feedback was generally reported in overwhelmingly negative terms.	‘The word “overweight” has a negative connotation no matter if that’s the intention … if you are being told your child is overweight you are going to find that a negative experience.’ p. 2887 U
There was a sense among some that the programme had overstepped its role.	‘How dare somebody tell me that my child is over-weight … to be sent home with healthy eating leaflets, blah blah, you just think “actually?” I just felt it was a little bit too much.’ p. 2887 U
With some parents reporting a sense of being judged.	‘It made me feel a little bit like I just feed my kids chips all the time, not a healthy balanced diet and I did feel a bit like I had had my fingers slapped.’ p. 2887 U
**12. Gillison F, Beck F, Lewitt J. Exploring the basis for parents’ negative reactions to being informed that their child is overweight. Public Health Nutr. 2014;17(5):987–97.^58^**	
Lack of belief in judgement/Lifestyle not included in assessment	“My child exercises every day of the week with horse riding and running and as you should know muscle weighs heavier than fat.’ ‘You do not take into account any exercise a child does. My child is very tall and horse rides twice a week and jogs every night.’” p. 990 U
Lack of belief in judgement/Child is naturally large	‘If you look at the rest of his activities and family members then his natural weight and body size is large.’ p. 990 U
Lack of belief in judgement/Puberty	‘My daughter is 11 years old and going through puberty when girls develop ‘‘puppy fat’’. She is healthy (eating wise) and active.’ p. 990 U
Lack of belief in judgement/BMI is not a valid measure	“BMI is not always a fair assessment; it depends on different ethnic groups and also in muscular people. ‘BMI is [an] outmoded and blunt instrument.’” p. 990 U
Lack of belief in judgement/Isolated measurement	‘If weighed every year and there was a pattern of being overweight then action should be taken. But I strongly feel that one weight during Year 6 should not lead to a child being labelled overweight.’ p. 990 U
Lack of belief in judgement/Normal relative to peers	There are much fatter children out there and my son isn’t that bad!’ p. 990 C
Belief that the judgement is unwarranted/Risk of harm	‘By telling children they are overweight you are causing eating disorders with Year 6 children where hormones are running riot and they are already very conscious of their body shapes.’ p. 990 U
Belief that the judgement is unwarranted/Physical activity and lifestyle mitigate risks	‘[My] child [is] at the age where a tiny weight gain is acceptable – as long as [his] diet [is] under control this will disappear.’ p. 990 U
Belief that the judgement is unwarranted/Child will naturally grow out of being overweight	‘He is very short for his age and I feel he will even out as he grows.’ p. 990 U
Belief that the judgement is unwarranted/Lack of belief that weight is a risk to health	‘I am heartily sick of the body fascism being dished out to children within school…Dieting and this paper is nonsense as you are expecting compliance to get people to look a certain way or punish them, if they don’t.’ p. 990 C
Belief that the judgement is unwarranted/Parental responsibility	When the letter arrives it seems like a bolt out of the blue and that you are being heavily criticised as an irresponsible parent.’ p. 990 U
**13. Gorlick JC, Gorman CV, Weeks HM, Pearlman AT, Schvey NA, Bauer KW. “I feel like less of a mom”: experiences of weight stigma by association among mothers of children with overweight and obesity. Child Obes. 2021;17(1):68–75.^59^**	
These mothers expressed feelings of blame, embarrassment, and shame when interacting with their children’s physicians.	Some mothers shared that they ‘‘dread’’ making appointments for their children because they know they are going to get ‘‘the lecture’’ about their children’s weight. p. 71 C
Some expressing frustration that physicians incorrectly assumed they were feeding their children unhealthy foods.	NS
Another mother said her child’s physician was ‘‘very, you know, nice about it’’ when talking about her child’s weight, but that the conversations made her…	… ‘‘feel as a parent like crap. Like my child’s not perfect and it’s my fault.’’ p. 72 C
Feeling unheard and questioned by physicians, particularly with respect to children’s diets, was a common experience by mothers.	‘‘It just makes me feel like I’m less than a mom… Like I didn’t make the right choices for them and that’s why they’re like that.” p. 72 U
**14. Hardy K, Hooker L, Ridgway L, Edvardsson K. Australian parents’ experiences when discussing their child’s overweight and obesity with the maternal and child health nurse: a qualitative study. J Clin Nurs. 2019;28(19–20):3610–7.^60^**	
They did not get enough information on how to tackle the problem at home.	“brush over” or “skate around” p. 3613 C
It was commonly described that the MCH nurse was quick to record the child’s weight and BMI, and point the BMI out on the chart, assuming the mother was already aware of the problem.	NS
Most mothers felt the MCH nurse did not ask them questions to determine the full details of the daily diet and exercise routines of their toddlers.	“tick the box” p. 3613 C
In nearly every interview, mothers commented that the MCH nurse should take a more holistic approach and gather more information on the family before providing BMI results and making recommendations.	The opening is there to discuss weight, food, activity when you’re actually weighing and measuring the child. p. 3613 C
No clearly written weight management strategies or care plans were provided. This lack of detail and direction caused frustration among mothers, as they did not know how to manage the situation.	I’m like a duck with my legs going crazy under water, and I look like I’m doing fine obviously… p. 3613 C
According to the participants, MCH nurses either did not discuss the child’s identified issue or lacked the communication skills to discuss it adequately.	NS
Many mothers identified time constraints in child health care as a reason for the superficial discussions about children’s weight.	NS
They felt the MCH nurse gave them minimal information regarding nutrition, exercise, and how to combat current problems.	NS
All mothers within the study suggested that MCH nurses need to consider he sensitive nature of the topic and take a strengths‐based, partnership approach with each individual.	NS
They also suggest offering support and discussing strategies for working together to identify solutions, rather than passing judgement.	Don’t associate increased weight with blame, rather acknowledge it’s something to work on together. p. 3613 C
Mothers suggested there are certain words the MCH nurse should avoid.	It’s a stigma, and stigma shouldn’t be placed on children. p. 3613 C
The majority of mothers who saw a different MCH nurse for either their 2‐year or 3‐and‐a‐half‐year visit, the experience was very different, and many perceived the consultation as damaging.	I didn’t want the comments to make me upset so I closed myself off to the health nurse and didn’t take anything on board. p. 3614 C
Mothers with no awareness of the child being overweight reported that they were either in shock, which disabled their listening skills so they could no longer absorb any information the MCH nurse gave them, or they reported feeling inadequate as a mother and that they had let their child down.	I felt as though the health nurse was blaming me, that was exactly how it felt. p. 3614 C
One mother in particular felt that the MCH nurse was discriminatory towards her son.	We don’t use the ‘o’ word, but your son is in the high category of overweight (Participant 10 ‐ quoting her MCH nurse) p. 3614 C
The majority of mothers interviewed had limited understanding of the concept of BMI or what constituted a “normal” BMI or healthy weight range for a toddler and felt that the MCH nurse had not taken the time to explain it to them.	NS
Some mothers reported partners dismissed the advice given because of the manner in which the MCH nurse had talked about the child’s weight.	NS
The majority of mothers felt it was to offer evidence‐based information and support when discussing children’s weight, while not being judgemental.	(A) nurse is the ideal person to discuss a child’s weight. They are up‐to‐date, they are someone you have built a rapport with. p. 3614 C
The participants raised the importance of continuity of care and in particular, the interpersonal relationship aspect.	Would want to see the same health nurse for all children, as it’s more about being comfortable with continuity. p. 3615 U
These mothers explained they felt as though The relieving nurse was there to “tick‐the‐boxes” and was very direct with questioning, rather than trying to establish any rapport.	What’s the point of the health nurse now? I can weigh and measure him and all the pamphlets are now available online. p. 3615 U
**15. Jones KM, Dixon ME, Dixon JB. GPs, families and children’s perceptions of childhood obesity. Obes Res Clin Pract. 2014;8(2):e140–8.^61^**	
Raising the topic (of obesity)	One parent stated that she ‘‘tries to make sure she [her daughter] is not in a consultation when [the topic] is brought up because I’ve had a weight problem all my life and I don’t want her to start to think like that already’’ p. e143 C
Frustrations experienced by families	‘‘it would be great if the GPs had a programme or something, something to follow or something to write down and measure the weight and the activities, to go by’’. p. e144 C
Sustaining improvements	‘‘he really cares about our child, so it’s a good thing for everyone to adopt’’ p. e146 U
**16. Jorda ML. The meaning of school body mass index (BMI) screening and referral to the parents/guardians of first, third, and sixth grade students. ProQuest Dissertations Publishing; 2015.^62^**	
Ensuring accuracy.	“I thought it was incorrect because if you look at my son he is tall, slender and very active.” p. 127 U
Communicating with sensitivity.	“obese is a dirty word. Sixth graders have their own issues. Their bodies are changing.” p. 130 U
**17. Laurent JS. A qualitative exploration into parental recognition of overweight and obesity in pre-adolescents: a process of discovery. J Pediatr Health Care. 2014;28(2):121–7.^63^**	
Some parents described a hostile approach that left them feeling blamed, shamed, and isolated.	“She said in front of my daughter—her exact words—’You are very obese. You are very obese.’’ And then she pulled out the chart. They had the height and the weight and all that, and told Nina she was very obese, very fat, and needed to lose a tremendous amount of weight. Nina got very upset and started to cry. [The HCP] was nasty with it. She was really nasty. p. 124 U
Other parents described an indiscreet approach by clinicians.	her HCP told her and her daughter that her daughter was ‘‘heavier than according to what she should be.’’ p. 124 C
**18. Lupi JL, Haddad MB, Gazmararian JA, Rask KJ. Parental perceptions of family and pediatrician roles in childhood weight management. J Pediatr. 2014;165(1):99-103.e2.^64^**	
Parents had mixed definitions of overweight and obesity and were confused about BMI	“[it] has to do with BMI and percent body fat, but I’m not sure exactly what.” Table III, p. 103.e2 U
Parents were well informed about the health implications of excess weight in children	“Increased blood pressure, diabetes, cholesterol — anything that could happen to adults could happen to kids.” Table III, p. 103.e2 U
Parents were most interested in dietary management, meal planning, and nutritional guidance	“…give us some sort of guideline to follow, that’s more than just a discussion, like a menu for a child his age.” Table III, p. 103.e2 U
Parents engaged in weight management with their pediatrician were happy with the suggestions and working to incorporate them into their behavior	“Well, they always give good nutritious advice, like to take them to the park for more long walks, to get her to dance more.” Table III, p. 103.e2 U
**19. Morenz-Harbinger Deborah L Ayer. Collaboration with parents to improve outcomes in young child obesity. ProQuest Dissertations Publishing; 2013.^65^**	
Parents felt they received too much information at the initial visit and could not retain it all because the child was acting out or the parents were overwhelmed with the information.	NS
More understanding with their situation and create individual plans to better suit their needs.	NS
Additional explanations about weighing the child in different parts of the program	NS
More open about their practice, listen to their parent/child families, and respond to why the medical staff conducted the procedures at every visit.	NS
Better understanding on the part of medical staff in listening to their concerns and treating them with respect.	NS
Medical staff could provide the needed information for successful treatment to the families in a non-judgmental way because some parents felt judged by previous visits.	NS
**20. Moyer LJ, Carbone ET, Anliker JA, Goff SL. The Massachusetts BMI letter: a qualitative study of responses from parents of obese children. Patient Educ Couns. 2014;94(2):210–7.^66^**	
Provides no new information	“Yeah, I mean I would look at it, but it’s not telling me anything that I probably don’t already know.” p. 214 U
Letter is helpful, acceptable	“It’s actually good that they do it because you get to see – back when you’re younger they never did this so – you just get to see where they compare and where they should be.” p. 214 U
Questionable validity of BMI	‘‘BMI may not tell the whole story about your child’s weight… An athletic child with a lot of muscle may have a high BMI but not be overweight.’’ p. 212 U
Interpreting the child’s weight status	“I have to read it a couple times before I actually knew how to understand the graph… Be more forward to it: Your son falls right here.” p. 214 U
Impact on child’s self-esteem	“When we get those BMIs from the school… I don’t like the fact that my son gets to see that before I can look at it… they don’t send it home in an envelope.” p. 214 C
Appropriate words used	“They just said my daughter was gaining X amount of weight… They didn’t say anything negative. They just said that they took her weight from previous years and then added it up to the next physical year.” p. 214 U
Helpful feedback and support	“I don’t think I ever had any negative, any kind of feedback is good feedback… just telling what kinds of foods to eat, the portion size, how often to eat, what time not to eat at.” p. 214 U
Addressed medical causes, consequences	“I’ve had a good experience… they’ve been so polite with her… giving hope to her that she can lose weight and feel the way that she wants… for my granddaughter, that wasn’t only what we were eating, was the new medication that she was taking increased her appetite and she gained weight, a lot of weight.” p. 214 U
Could be helpful, motivating	“This is very positive, and I think everybody should get it and get screened… I’m just glad the school noticed and they’re out there to help the kids with their weight.” p. 214 U
Inappropriate words used	“The word that I don’t like – it was used for me, but also for my kid – obese. I don’t like that word, I really don’t like that word.” p. 214 U
Inadequate response to weight concerns	“I had a concern with my son’s previous pediatrician… and he said he’ll grow out of it, he’ll grow out of it – until recently, a couple of years ago, we changed the pediatrician and the first thing this new pediatrician said to us was the weight issue.” p. 214 U
**21. Nnyanzi LA, Summerbell CD, Ells L, Shucksmith J. Parental response to a letter reporting child overweight measured as part of a routine national programme in England: results from interviews with parents. BMC Public Health. 2016;16:846.^67^**	
These parents/guardians reported that it had never occurred to them that anyone would regard their children as overweight/obese and Felt that it added to the ‘insult’ to see words like ‘overweight’ and ‘obese’ in bold letters.	…I was so shocked when I opened the letter and I read the contents because I always thought that my son was the right weight. Honestly, I have had no concerns at all about his weight or about what he eats or anything, so I didn’t even tell him we got it. I hid it away, because I don’t want to encourage him to be so conscious of his weight and I think he has the right attitude towards food and exercise. p. 7 U
Some parents/guardians (n = 3) chose to throw away the letter, determined not to let their children see it, as they feared that this could impact on their children’s self-esteem and mental wellbeing.	…all along I thought my son was absolutely fine and then it had on the letter that he was very overweight and the certain illnesses that he could get when he is older which I was quite horrified about, and then I put it in the bin so he couldn’t see it cos I didn’t wanna worry him. Obviously he is old enough to read. You know what I mean? p. 7 U
But some parents/guardians (n = 2) could not just let the feedback go without some response, so they took it upon themselves to write back to the authorities describing Disgust about the letter they had received.	To whom it may concern, I would like to express my disgust after receiving a letter from the school nursing team stating that my daughter is overweight. At 11 years of age she is becoming increasingly aware of her image and is self-conscious about the way she looks. Thankfully she did not see the letter we received stating in bold lettering that she was supposedly overweight. Had she done so, I think it would have been a real blow to her confidence, possibly with detrimental effects to her health, as she may decide to change her very healthy attitude to eating and exercise? I think letters such as this can be as damaging as they can be helpful. I have no concerns about my daughter’s weight and believe that she is sufficiently active to maintain a healthy weight. Yours sincerely, p. 8 U
This underscores the importance of following up the weight feedback to try and provide the opportunity for parents/guardians to vent their anger.	…I am glad that I have had the opportunity to speak to you about it, because at the time I did feel very strongly about it, but maybe there were some other parents that felt as strongly but just didn’t do anything about it afterwards and just sort of went, ‘Oh well’ p. 9 U
**22. Peña CM, Payne A. Parental experiences of adopting healthy lifestyles for children with disabilities living with overweight and obesity. Disabil Health J. 2022;15(1):101215.^68^**	
Inconsistent advice	“The GP has mentioned that she is a bit overweight, and the consultant at the gastro clinic mentioned that she was overweight but they didn’t really say anything [laughter]” p. 4 U
Fear of judgement and reassurance	“before that I was just worrying, but seeing her (dietitian) reassured me that actually I was doing the right thing following the right path you know, and just to carry on and just you know monitor it” P. 5 C
Practical and individualized advice	“The Change4Life leaflets didn’t really help us because all the things that it says not to do, we weren’t doing anyways”… “so that kind of was irrelevant to us” p. 4 U
**23. Povey R, Cowap L, Scholtens K, Forshaw M. ‘She’s not obese, she’s a normal 5-year-old and she keeps up with the other kids’: families’ reasons for not attending a family-based obesity management programme. Perspect Public Health. 2020;140(3):148–52.^69^**	
Communication	“I felt people were telling me that I wasn’t bringing up Alana in the correct manner.” p. 149 C
Negative emotional reactions	I was disgusted so I got in touch with the school nurse and expressed my anger. p. 149 U
‘I didn’t think my son was overweight’	And I think ‘she’s not obese, she’s a normal 5-year-old and she keeps up with the other kids’. p. 150 U
Programme not needed	She eats a lot of fruit and a lot of veg, she’d rather eat that than sweets to be honest, so I didn’t see how that would help. p. 150 U
Approach too generic	Have you seen her swing off trees and all that? If she was that obese she wouldn’t be able to that. p. 150 C
‘It’s a big worry to a 4-year-old’	There’s too many kids with problems and eating disorders and things like that and I don’t want that. p. 151 C
**24. Schalkwijk A, Bot S, De Vries L, Westerman M, Nijpels G, Elders P. Perspectives of obese children and their parents on lifestyle behavior change: a qualitative study. Int J Behav Nutr Phys Act. 2015;12(1):102.^70^**	
The general practitioner should play an active role not only in signalling the weight problem in time, but also in offering ongoing support.	“It would have been nice if the GP would have asked how we were doing during the program. That would have been an extra stimulant for him.” p. 6 U
**25. Schwartz M. Parental perceptions of body mass index notification: a qualitative study. J Sch Health. 2015;85(10):714–21.^71^**	
Different feelings about receiving the letter.	‘‘It’s kind of like a double-edged sword. You know, it was probably appropriate to send that out to us, but I didn’t want to receive it.’’ p. 716 U
PCP took the time to speak with them about ways to keep healthy by eating well and being.	NS
They felt the PCPs knew their child’s growth patterns, activity levels, and general eating habits, and these types of reactions were reassuring to them.	“Not to worry about it”. p. 718 C
The parents who described their interaction as negative verbalized frustration and felt the PCPs did not do anything to help them.	NS
**26. Syrad H, Falconer C, Cooke L, Saxena S, Kessel AS, Viner R, *et al.* “Health and happiness is more important than weight”: a qualitative investigation of the views of parents receiving written feedback on their child’s weight as part of the National Child Measurement Programme. J Hum Nutr Diet J Hum Nutr Diet. 2015;28(1):47–55.^72^**	
Broad definitions of healthy.	‘It only measures weight to height ratio and doesn’t take into account their lifestyle, their activity and things like that’ p. 50 U
**27. Toftemo I, Glavin K, Lagerløv P. Parents’ views and experiences when their preschool child is identified as overweight: a qualitative study in primary care. Fam Pract. 2013;30(6):719–23.^73^**	
Parents being vulnerable.	Of course, it’s difficult. Nobody wants to hear there’s something wrong with their child. There is almost nothing that can make parents more terribly unhappy, hurt and angry like that. And you would like to defend… Still it’s so important how it’s presented to you. If I was to say to someone that “Now your kid is too fat – there is too much candy, too much fatty food and too much sitting in front of the TV”, then I would for sure not accomplish anything, right? p. 721 U
Relationship with the child.	Then I talked to her (GP) in the corner of the room, and told her that I thought talking about weight was difficult. We agreed on setting up a new appointment so that I could talk with her without him (the child) being present. p. 721 U
Dialogue with health professionals.	If overweight had been an issue and you could see that things were going in the wrong direction, I would hope for more focus and closer follow-ups. Perhaps we could make a plan about what to do. And then we would have a shared responsibility to carry it out. p. 722 U
**28. Turer CB, Mehta M, Durante R, Wazni F, Flores G. Parental perspectives regarding primary‐care weight‐management strategies for school‐age children. Matern Child Nutr. 2016;12(2):326–38.^74^**	
Use discretion in weight discussions	‘When a doctor says to the child, “You weigh this much and we need to work on a diet,” my daughter saying, “I’m overweight.” Then she lookin’ in the mirror – she’s only seven years old. Don’t talk about it in front of her; she’s a child. She should be going to school, playing, not worrying about her weight.’ p. 333 U
Discuss weight-related health risks	‘The doctors have good resources and good referrals. [My son] actually learned why sugar is a big deal, and cholesterol issues. And that’s something I don’t think about. I need to let him know, “This sugar thing {prediabetes} that you’re having is very serious,” without scaring him.’ p. 333 U
Do not use scare tactics	‘One of the doctors said that the way I been feeding her is like the same thing as giving her drugs. I said, “No, it’s totally different.” It made me upset.’ p. 333 U
Weight-status improvement plan	‘I would talk to her about how much she’s overweight and needs to lose, and how to keep it off.’ p. 333 U
Continually monitor child’s progress (not just weight)	‘Develop a strong relationship with them [the child and family]. Then, check up on them – how they are doing, and if they can do it [the weight-management strategy]. Don’t just say, “Okay, let’s weigh your child again.” We know she’s overweight.’ p. 333 U
Provide encouragement	‘Check in. It would be nice just so she could say, “Well if I do this, the doctor will be proud of me.”’‘You need that coaching, “You’re doing a good job.” You need to hear that as parents.’ p. 333 U
**29. Turer CB, Upperman C, Merchant Z, Montaño S, Flores G. Primary-care weight-management strategies: parental priorities and preferences. Acad Pediatr. 2016;16(3):260–6.^75^**	
Advice/weight	“Stress importance of weight loss, relate to her the damage being overweight can cause to her body and mental state.” p. 263 C
Risk factors/consequences	“Talk to [the] child about being overweight, including risks of being overweight.” p. 263 U
Communicate with parent	“Talk to me directly, be completely honest about how serious his case may be.” p. 263 U
Communicate with child	“Talk to [chil The use of the child’s weight chart in the conversation helped to dedramatize but at the same time capture the seriousness of the conversation dren] one-on-one so they understand.” p. 263 U
**30. Turner KM, Salisbury C, Shield JPH. Parents’ views and experiences of childhood obesity management in primary care: a qualitative study. Fam Pract Fam Pr. 2012;29(4):476–81.^76^**	
They were concerned the consultation would have a negative impact on their child’s mental well-being.	I didn’t want to take [daughter] to the doctor’s was because I was overweight as a child… I didn’t want her to get that, you know, like embarrassed and the way that I used to feel. p. 478 C
In contrast, monitoring of a child’s height and weight was described as giving the child a ‘complex’ since it was done in the absence of any advice, and it was apparent that lifestyle advice could be dismissed as unnecessary.	They [the GP] just says ‘oh, give her exercise, make her walk more.’ But she walks to school every day and its right down the bottom, and she walks home, goes to the park on her way home. p. 479 C
GP had caused offence in the way they had handled the consultation.	He [the GP] said in front of [daughter], ‘God she’s obese, how on earth can you let her get that size?’ You know, ‘You’ve just simply got to cut down, you’re giving her the wrong foods,’ and ‘Do you realise how much health issue that is?’ You know, ‘She shouldn’t be that size,’… I took the kids out, went back in and said it was absolutely disgraceful, no way would I take the children back there again. p. 479 U
In contrast, schools nurses were described as having met with the child or child and parent on several occasions, and in addition to monitoring the child’s weight and referring them to secondary care Working on issues such as low self-esteem and behavioural problems, which parents reported as helpful.	NS
**31. Visram S, Hall TD, Geddes L. Getting the balance right: qualitative evaluation of a holistic weight management intervention to address childhood obesity. J Public Health. 2013;35(2):246–54.^77^**	
Shock that their child had been identified as overweight or obese	But how we found out about it was because we received a letter though the post to say that [name of son] was classed as clinically obese. Obese. And to go on this ‘Balance It!’ scheme. […] To me, [name] was never obese. And that’s, you know… I’m not looking at him through rose-tinted glasses here. [Name] is quite an active child, but he’s quite a broad child and he’s quite tall as well. So looking at him, anybody would have said that he wasn’t fat. p. 249 U
Parents felt that involving families in decision-making and maintaining regular communication could help to sustain motivation.	Well, the suggestions that I would do would be to involve the whole family. For it to be taken as a holistic approach to the family – not just for the one child. Maybe if, you know, we [the parents] were offered a free swim as well, we would have went swimming a lot more as a group. p. 251 U
**32. Wagner E, Jamil O, Hodges B. Talking about childhood obesity. Clin Pediatr (Phila). 2022;61(3):266–9.^78^**	
Discussing weight in nonlabeling language was preferred.	(doctors) “did a fantastic job of not making (child) feel bad”, doctors said things in “positive ways” p. 267 C
Direct and empathic communication styles are most effective.	You’re doing well, keep it up. p. 268 C
Cultural humility and understanding.	NS
**33. Wild CE, Rawiri NT, Willing EJ, Hofman PL, Anderson YC. Determining barriers and facilitators to engagement for families in a family-based, multicomponent healthy lifestyles intervention for children and adolescents: a qualitative study. BMJ Open. 2020;10(9):e037152.^79^**	
The age of the child involved in the service affected the degree to which families chose to engage, due to a perception that children were too young to have weight problems.	‘Like, a weight problem, like, at the time he was only 6 years or 7 years’.‘… we were kind of shocked because they said that [SON] was, like, obese or something … I don’t think he’s overweight at all … Because he’s really tall … so I don’t understand, like, what sort of weight should he have been because he was, he’s just like a, he was like a normal kid. So I don’t understand what is overweight and underweight. Because I’ve seen some, not being mean, but overweight kids, and he wasn’t overweight’ p. 4 U
Their child might not fit into a set of assessment criteria, this did not necessarily equate to their child being unhealthy.	When he got put in the […] ‘oh, he’s overweight’ box. And when you’re, like, ‘he’s not that overweight’, because it was just he wasn’t in their little boxes. I think that more annoyed me, is that they’ve got these sort of, like, ‘this is the normal weight for a 5 year old’. Well, there’s all sorts of different 5 year olds. He’s now 10 years and he is my height […] he’s a big guy. p. 5 U
Weight stigma and discrimination.	‘… having visited for something else entirely different and then being told kind of ‘your child’s obese and we are going to refer you’ and just doing it front of him […] it was just even in the way that it was delivered and I was kind of not expecting it. I mean, I can see that he’s, he’s a bit chunky, but I just, I don’t know […] [the referral] was a bit off- putting’. p. 4 C
If participants had had negative experiences in the health system in relation to their weight or ethnicity, then they were less willing to engage.	Basically they told her she was obese [at the B4 School Check] … Yeah, that she was obese for her age and they said this in front of her, and she was like ‘what is obese’? And they said, ‘you’re bigger than any other child your age’ but she’s not the only one […] So they say it in front of a child, it sort of knocks their self-esteem and their confidence right back. p. 5 C
Compassionate and respectful care.	‘It was not just the families, but also the, what do you call them, the workers … Very supportive, non-judgmental. I think that made a big difference and ‘yes we are going to go’ because they are not judging you … the staff was very supportive’. p. 4 U

U, unequivocal; C, credible; NS, not supported

BMI, body mass index; CHC, child health care; CHS, child health service; GP, general practitioner; HCP, health care professional; MCH, maternal and child health; PCP, primary care provider; WIC, Special Supplemental Nutrition Program for Women, Infants, and Children.

## References

[1] Zolnierek KBH DiMatteo MR. Physician communication and patient adherence to treatment: a meta-analysis. Med Care 2009;47(8):826–34. 19584762 10.1097/MLR.0b013e31819a5acc PMC2728700

[2] Jangland E Gunningberg L Carlsson M. Patients’ and relatives’ complaints about encounters and communication in health care: evidence for quality improvement. Patient Educ Couns 2009;75(2):199–204. 19038522 10.1016/j.pec.2008.10.007

[3] Borrelli B Tooley EM Scott-Sheldon LAJ. Motivational interviewing for parent-child health interventions: a systematic review and meta-analysis. Pediatr Dent 2015;37(3):254–65. 26063554

[4] Weghuber D Khandpur N Boyland E Mazur A Frelut ML Forslund A Championing the use of people‐first language in childhood overweight and obesity to address weight bias and stigma: a joint statement from the the European‐Childhood‐Obesity‐Group (ECOG), the European‐Coalition‐for‐People‐Living‐with‐Obesity (ECPO), the International‐Paediatric‐Association (IPA), Obesity‐Canada, the European‐Association‐for‐the‐Study‐of‐Obesity Childhood‐Obesity‐Task‐Force (EASO‐COTF), Obesity Action Coalition (OAC), The Obesity Society (TOS) and the World‐Obesity‐Federation (WOF). Pediatr Obes 2023;18(6):e13024. 37002830 10.1111/ijpo.13024

[5] Puhl RM. What words should we use to talk about weight? A systematic review of quantitative and qualitative studies examining preferences for weight‐related terminology. Obes Rev 2020;21(6):e13008. 32048465 10.1111/obr.13008

[6] World Health Organization. Obesity and overweight [internet]. WHO; 2021 cited [2023 Oct 23]. Available from: https://www.who.int/news-room/fact-sheets/detail/obesity-and-overweight.

[7] Ma L Chu M Li Y Wu Y Yan AF Johnson B Bidirectional relationships between weight stigma and pediatric obesity: a systematic review and meta‐analysis. Obes Rev 2021;22(6):e13178. 33533189 10.1111/obr.13178

[8] Wu Y Berry DC. Impact of weight stigma on physiological and psychological health outcomes for overweight and obese adults: a systematic review. J Adv Nurs 2018;74(5):1030–42. 29171076 10.1111/jan.13511

[9] Dam R Robinson HA Vince-Cain S Heaton G Greenstein A Sperrin M Engaging parents using web-based feedback on child growth to reduce childhood obesity: a mixed methods study. BMC Public Health 2019;19(1):300. 30866878 10.1186/s12889-019-6618-3 PMC6415344

[10] Puhl RM Peterson JL Luedicke J. Parental perceptions of weight terminology that providers use with youth. Pediatrics 2011;128(4):e786–93. 21949145 10.1542/peds.2010-3841

[11] Adab P Pallan M Whincup PH. Is BMI the best measure of obesity? BMJ 2018;360:k1274. 29599212 10.1136/bmj.k1274

[12] Hampl SE Hassink SG Skinner AC Armstrong SC Barlow SE Bolling CF Clinical practice guideline for the evaluation and treatment of children and adolescents with obesity. Pediatrics 2023;151(2):e20220 60640. 10.1542/peds.2022-060640 36622115

[13] Nuttall FQ. Body mass index: obesity, BMI, and health: a critical review. Nutr Today 2015;50(3):117–28. 27340299 10.1097/NT.0000000000000092 PMC4890841

[14] Provvidenza CF Hartman LR McPherson AC. Fostering positive weight‐related conversations between health care professionals, children, and families: development of a knowledge translation Casebook and evaluation protocol. Child Care Health Dev 2019;45(1):138–45. 30376689 10.1111/cch.12627

[15] Mikhailovich K Morrison P. Discussing childhood overweight and obesity with parents: a health communication dilemma. J Child Health Care 2007;11(4):311–22. 18039733 10.1177/1367493507082757

[16] Newson L Povey R Casson A Grogan S. The experiences and understandings of obesity: families’ decisions to attend a childhood obesity intervention. Psychol Health 2013;28(11):1287–305. 23758103 10.1080/08870446.2013.803106

[17] Falconer CL Park MH Croker H Skow Á Black J Saxena S The benefits and harms of providing parents with weight feedback as part of the national child measurement programme: a prospective cohort study. BMC Public Health 2014;14(1):549. 24888972 10.1186/1471-2458-14-549 PMC4057922

[18] Etelson D Brand DA Patrick PA Shirali A. Childhood obesity: do parents recognize this health risk? Obes Res 2003;11(11):1362–68. 14627757 10.1038/oby.2003.184

[19] Vanhala ML Keinänen-Kiukaanniemi S M Kaikkonen KM Laitinen JH Korpelainen RI. Factors associated with parental recognition of a child’s overweight status - a cross sectional study. BMC Public Health 2011;11(1):665. 21864365 10.1186/1471-2458-11-665 PMC3173349

[20] Ames H Mosdøl A Blaasvær N Nøkleby H Berg RC Langøien LJ. Communication of children’s weight status: what is effective and what are the children’s and parents’ experiences and preferences? A mixed methods systematic review. BMC Public Health 2020;20(1):1–22. 32345274 10.1186/s12889-020-08682-w PMC7189728

[21] Estabrooks PA Shoup JA Gattshall M Dandamudi P Shetterly S Xu S. Automated telephone counseling for parents of overweight children. Am J Prev Med 2009;36(1):35–42.e2. 19095163 10.1016/j.amepre.2008.09.024

[22] Sharifi M Dryden EM Horan CM Price S Marshall R Hacker K Leveraging text messaging and mobile technology to support pediatric obesity-related behavior change: a qualitative study using parent focus groups and interviews. J Med Internet Res 2013;15(12):e272. 24317406 10.2196/jmir.2780 PMC3869083

[23] Andreassen P Grøn L Roessler KK. Hiding the plot: parents’ moral dilemmas and strategies when helping their overweight children lose weight. Qual Health Res 2013;23(10):1333–43. 24019307 10.1177/1049732313505151

[24] Eli K Howell K Fisher PA Nowicka P. “Those comments last forever”: parents and grandparents of preschoolers recount how they became aware of their own body weights as children. PLoS One 2014;9(11):e111974. 25393236 10.1371/journal.pone.0111974 PMC4230937

[25] Ek A Nordin K Nyström CD Sandvik P Eli K Nowicka P. Responding positively to “children who like to eat”: parents’ experiences of skills-based treatment for childhood obesity. Appetite 2020;145:104488. 31626835 10.1016/j.appet.2019.104488

[26] Thomson J Percival T Lilo LS Smith M. What are the success factors for children with obesity having obesity discussions? A cross-sectional multiple methods study in an emergency setting. Emerg Nurse NZ 2020:8–15.

[27] Bradbury D Chisholm A Watson PM Bundy C Bradbury N Birtwistle S. Barriers and facilitators to health care professionals discussing child weight with parents: a meta‐synthesis of qualitative studies. Br J Health Psychol 2018;23(3):701–22. 29700900 10.1111/bjhp.12312 PMC6099303

[28] Sjunnestrand M Nordin K Eli K Nowicka P Ek A. Planting a seed - child health care nurses’ perceptions of speaking to parents about overweight and obesity: a qualitative study within the STOP project. BMC Public Health 2019;19(1):1494. 31706318 10.1186/s12889-019-7852-4 PMC6842180

[29] Aamann IC Erlik M. ‘Am I that bad?’: middle‐class moralism and weight stigma towards parents of children with higher weight. Child Soc 2023;37(6):1737–53.

[30] McPherson AC Hamilton J Kingsnorth S Knibbe TJ Peters M Swift JA Communicating with children and families about obesity and weight-related topics: a scoping review of best practices: talking about obesity with children. Obes Rev 2017;18(2):164–82. 27888564 10.1111/obr.12485

[31] McPherson AC Knibbe TJ Oake M Swift JA Browne N Ball GDC “Fat is really a four-letter word”: exploring weight-related communication best practices in children with and without disabilities and their caregivers. Child Care Health Dev 2018;44(4):636–43. 29761539 10.1111/cch.12575

[32] Barlow SE. the Expert Committee. Expert committee recommendations regarding the prevention, assessment, and treatment of child and adolescent overweight and obesity: summary report. Pediatrics 2007;120(Suppl 4):S164–92. 18055651 10.1542/peds.2007-2329C

[33] Van Maarschalkerweerd PEA Camfferman R Seidell JC Halberstadt J. Children’s, parents’ and healthcare professionals’ preferences for weight-based terminology in health care. Health Commun 2021;36(13):1805–09. 32722954 10.1080/10410236.2020.1796282

[34] Carcone AI Jacques-Tiura AJ Brogan Hartlieb KE Albrecht T Martin T. Effective patient–provider communication in pediatric obesity. Pediatr Clin North Am 2016;63(3):525–38. 27261548 10.1016/j.pcl.2016.02.002 PMC4893931

[35] Farnesi BC Newton AS Holt NL Sharma AM Ball GDC. Exploring collaboration between clinicians and parents to optimize pediatric weight management. Patient Educ Couns 2012;87(1):10–17. 21925825 10.1016/j.pec.2011.08.011

[36] Monaghan LF Rich E Bombak AE. Media ‘Fat panic’ and public pedagogy: mapping contested terrain. Sociol Compass 2019;13(1):1-17.

[37] Quirke L. Fat-proof your child”: Parenting advice and “child obesity. Fat Stud 2016;5(2):137–55.

[38] Kokkonen R. The fat child-a sign of ‘bad’ motherhood? An analysis of explanations for children’s fatness on a Finnish website. J Community Appl Soc Psychol 2009;19(5):336–47.

[39] Koivumäki T Jallinoja P. The good, the bad, and the blameless in parenting: a thematic analysis of discussions of childhood obesity on an internet forum. BMC Public Health 2023;23(1):452. 36890492 10.1186/s12889-023-15314-6 PMC9993749

[40] Harris JK Moreland-Russell S Tabak RG Ruhr LR Maier RC. Communication about childhood obesity on Twitter. Am J Public Health 2014;104(7):e62–9. 10.2105/AJPH.2013.301860 PMC4056214 24832138

[41] Brix N Ernst A Lauridsen LLB Parner E Støvring H Olsen J Timing of puberty in boys and girls: a population‐based study. Paediatr Perinat Epidemiol 2019;33(1):70–78. 30307620 10.1111/ppe.12507 PMC6378593

[42] Wood CL Lane LC Cheetham T. Puberty: normal physiology (brief overview). Best Pract Res Clin Endocrinol Metab 2019;33(3):101265. 31000487 10.1016/j.beem.2019.03.001

[43] Kontochristopoulou AM Karatzi K Karaglani E Cardon G Kivelä J Iotova V Parental practices and children’s lifestyle correlates of childhood overweight/obesity in Europe: The Feel4Diabetes study. J Hum Nutr Diet 2024;37(1):31–46. 37828766 10.1111/jhn.13229

[44] Vega-Díaz M González-García H de Labra C. Influence of parental involvement and parenting styles in children’s active lifestyle: a systematic review. Peer J San Franc CA 2023;11:e16668–e16668. 10.7717/peerj.16668 PMC10749091 38144179

[45] Lockwood C Porritt K Munn Z Rittenmeyer L Salmond S Bjerrum M Systematic Reviews of Qualitative Evidence. In: Aromataris E Lockwood C Porritt K Pilla B Jordan Z, editors. JBI Manual for Evidence Synthesis [internet]. JBI; 2024 [cited 2024 Jun 8]. Available from: https://synthesismanual.jbi.global.

[46] Koivumäki T Kääriäinen M Tuomikoski AM Kaunonen M. Parent and carer experiences of health care professionals’ communication about childhood obesity: a qualitative systematic review protocol. JBI Evid Synth 2023;21(2):401–06. 36059227 10.11124/JBIES-22-00017 PMC9901846

[47] Åsberg M Derwig M Castor C. Parents’ recalled experiences of the child centred health dialogue in children with overweight: a qualitative study. BMC Health Serv Res 2023;23:289. 36973799 10.1186/s12913-023-09308-8 PMC10045090

[48] Akselbo I Ingebrigtsen O. Mothers of overweight children - experiences and challenges. Nordisk sygeplejeforskning 2015;5(4):453–63. [Norwegian]

[49] Munn Z Aromataris E Tufanaru C Stern C Porritt K Farrow J The development of software to support multiple systematic review types: the Joanna Briggs Institute System for the Unified Management, Assessment and Review of Information (JBI SUMARI). Int J Evid Based Healthc 2019;17(1):36–43. 30239357 10.1097/XEB.0000000000000152

[50] Small SP. Reflections on critical appraisal of research for qualitative evidence synthesis. JBI Evid Synth 2023;21(6):1064–65. 37282720 10.11124/JBIES-23-00198

[51] Anderson YC Wild CEK Hofman PL Cave TL Taiapa KJ Domett T Participants’ and caregivers’ experiences of a multidisciplinary programme for healthy lifestyle change in Aotearoa/New Zealand: a qualitative, focus group study. BMJ Open 2021;11(5):e043516. 10.1136/bmjopen-2020-043516 PMC8118004 33980517

[52] Ayash CR. Clinic-based interventions to address childhood obesity: part of the solution to a public health problem? ProQuest Dissertations Publishing; 2011.

[53] Banks J Cramer H Sharp DJ Shield JP Turner KM. Identifying families’ reasons for engaging or not engaging with childhood obesity services: a qualitative study. J Child Health Care 2014;18(2):101–10. 23728931 10.1177/1367493512473854

[54] Davidson K Vidgen H. Why do parents enrol in a childhood obesity management program? A qualitative study with parents of overweight and obese children. BMC Public Health 2017;17(1):159. 28153053 10.1186/s12889-017-4085-2 PMC5290615

[55] Eli K Neovius C Nordin K Brissman M Ek A. Parents’ experiences following conversations about their young child’s weight in the primary health care setting: a study within the STOP project. BMC Public Health 2022;22(1):1540. 35962359 10.1186/s12889-022-13803-8 PMC9375316

[56] Falbe J Friedman LE Sokal-Gutierrez K Thompson HR Tantoco NK Madsen KA. “She gave me the confidence to open up”: bridging communication by promotoras in a childhood obesity intervention for Latino families. Health Educ Behav 2017;44(5):728–37. 28851237 10.1177/1090198117727323

[57] Gainsbury A Dowling S. “A little bit offended and slightly patronised”: parents’ experiences of National Child Measurement Programme feedback. Public Health Nutr 2018;21(15):2884–92. 29914583 10.1017/S1368980018001556 PMC10260859

[58] Gillison F Beck F Lewitt J. Exploring the basis for parents’ negative reactions to being informed that their child is overweight. Public Health Nutr 2014;17(5):987–97. 24060095 10.1017/S1368980013002425 PMC10282330

[59] Gorlick JC Gorman CV Weeks HM Pearlman AT Schvey NA Bauer KW. “I feel like less of a mom”: experiences of weight stigma by association among mothers of children with overweight and obesity. Child Obes 2021;17(1):68–75. 33373542 10.1089/chi.2020.0199 PMC7815062

[60] Hardy K Hooker L Ridgway L Edvardsson K. Australian parents’ experiences when discussing their child’s overweight and obesity with the maternal and child health nurse: a qualitative study. J Clin Nurs 2019;28(19–20):3610–17. 31162886 10.1111/jocn.14956

[61] Jones KM Dixon ME Dixon JB. GPs, families and children’s perceptions of childhood obesity. Obes Res Clin Pract 2014;8(2):e140–8. 24743009 10.1016/j.orcp.2013.02.001

[62] Jorda ML. The meaning of school body mass index (BMI) screening and referral to the parents/guardians of first, third, and sixth grade students. ProQuest Dissertations Publishing; 2015.

[63] Laurent JS. A qualitative exploration into parental recognition of overweight and obesity in pre-adolescents: a process of discovery. J Pediatr Health Care 2014;28(2):121–27. 23419505 10.1016/j.pedhc.2012.12.010

[64] Lupi JL Haddad MB Gazmararian JA Rask KJ. Parental perceptions of family and pediatrician roles in childhood weight management. J Pediatr 2014;165(1):99–103.e2. 24721470 10.1016/j.jpeds.2014.02.064

[65] Morenz-Harbinger DLA. Collaboration with parents to improve outcomes in young child obesity. ProQuest Dissertations Publishing; 2013.

[66] Moyer LJ Carbone ET Anliker JA Goff SL. The Massachusetts BMI letter: a qualitative study of responses from parents of obese children. Patient Educ Couns 2014;94(2):210–17. 24290240 10.1016/j.pec.2013.10.016 PMC4553945

[67] Nnyanzi LA Summerbell CD Ells L Shucksmith J. Parental response to a letter reporting child overweight measured as part of a routine national programme in England: results from interviews with parents. BMC Public Health 2016;16:846. 27544538 10.1186/s12889-016-3481-3 PMC4992560

[68] Peña CM Payne A. Parental experiences of adopting healthy lifestyles for children with disabilities living with overweight and obesity. Disabil Health J 2022;15(1):101215. 34556445 10.1016/j.dhjo.2021.101215

[69] Povey R Cowap L Scholtens K Forshaw M. ‘She’s not obese, she’s a normal 5-year-old and she keeps up with the other kids’: families’ reasons for not attending a family-based obesity management programme. Perspect Public Health 2020;140(3):148–52. 31409189 10.1177/1757913919868509

[70] Schalkwijk A Bot S De Vries L Westerman M Nijpels G Elders P. Perspectives of obese children and their parents on lifestyle behavior change: a qualitative study. Int J Behav Nutr Phys Act 2015;12(1):102. 26283232 10.1186/s12966-015-0263-8 PMC4539727

[71] Schwartz M. Parental perceptions of body mass index notification: a qualitative study. J Sch Health 2015;85(10):714–21. 26331754 10.1111/josh.12300

[72] Syrad H Falconer C Cooke L Saxena S Kessel AS Viner R “Health and happiness is more important than weight”: a qualitative investigation of the views of parents receiving written feedback on their child’s weight as part of the National Child Measurement Programme. J Hum Nutr Diet 2015;28(1):47–55. 26295077 10.1111/jhn.12217 PMC4340048

[73] Toftemo I Glavin K Lagerløv P. Parents’ views and experiences when their preschool child is identified as overweight: a qualitative study in primary care. Fam Pract 2013;30(6):719–23. 24107270 10.1093/fampra/cmt056

[74] Turer CB Mehta M Durante R Wazni F Flores G. Parental perspectives regarding primary‐care weight‐management strategies for school‐age children. Matern Child Nutr 2016;12(2):326–38. 24720565 10.1111/mcn.12131 PMC4193944

[75] Turer CB Upperman C Merchant Z Montaño S Flores G. Primary-care weight-management strategies: parental priorities and preferences. Acad Pediatr 2016;16(3):260–66. 26514648 10.1016/j.acap.2015.09.001 PMC4808480

[76] Turner KM Salisbury C Shield JPH. Parents’ views and experiences of childhood obesity management in primary care: a qualitative study. Fam Pract 2012;29(4):476–81. 22117082 10.1093/fampra/cmr111

[77] Visram S Hall TD Geddes L. Getting the balance right: qualitative evaluation of a holistic weight management intervention to address childhood obesity. J Public Health 2013;35(2):246–54. 10.1093/pubmed/fds075 22967909

[78] Wagner E Jamil O Hodges B. Talking about childhood obesity. Clin Pediatr (Phila) 2022;61(3):266–69. 35001640 10.1177/00099228211070390

[79] Wild CE Rawiri NT Willing EJ Hofman PL Anderson YC. Determining barriers and facilitators to engagement for families in a family-based, multicomponent healthy lifestyles intervention for children and adolescents: a qualitative study. BMJ Open 2020;10(9):e037152. 10.1136/bmjopen-2020-037152 PMC7478027 32895279

[80] Munn Z Porritt K Lockwood C Aromataris E Pearson A. Establishing confidence in the output of qualitative research synthesis: the ConQual approach. BMC Med Res Methodol 2014;14(1):108. 25927294 10.1186/1471-2288-14-108 PMC4190351

[81] Page MJ McKenzie JE Bossuyt PM Boutron I Hoffmann TC Mulrow CD The PRISMA 2020 statement: an updated guideline for reporting systematic reviews. BMJ 2021;372:n71. 33782057 10.1136/bmj.n71 PMC8005924

[82] Lang S Gibson S Ng KW Truby H. Understanding children and young people’s experiences pursuing weight loss maintenance using the socio‐ecological model: a qualitative systematic literature review. Obes Rev 2021; 22(5):e13172. 33331090 10.1111/obr.13172

[83] Davis JL Goar C Manago B Reidinger B. Distribution and disavowal: managing the parental stigma of children’s weight and weight loss. Soc Sci Med 2018;219:61–69. 30391871 10.1016/j.socscimed.2018.10.015

[84] Pudney EV Puhl RM Halgunseth LC Schwartz MB. Parental reasons for engaging in or avoiding weight talk with children. Child Obes 2023;19(8):575–80. 36475982 10.1089/chi.2022.0173

[85] Lord SP Sheng E Imel ZE Baer J Atkins DC. More than reflections: empathy in motivational interviewing includes language style synchrony between therapist and client. Behav Ther 2015;46(3):296–303. 25892166 10.1016/j.beth.2014.11.002 PMC5018199

[86] Sela Y Grinberg K Nemet D. Obstacles preventing public health nurses from discussing children’s overweight and obesity with parents. Compr Child Adolesc Nurs 2022;45(4):425–36. 36440868 10.1080/24694193.2022.2117433

[87] Piggin J Lee J. ‘Don’t mention obesity’: contradictions and tensions in the UK Change4Life health promotion campaign. J Health Psychol 2011;16(8):1151–64. 21459920 10.1177/1359105311401771

[88] United Kingdom Government. National child measurement programme [internet]. Gov UK; 2023 cited [2023 Oct 23]. Available from: https://www.gov.uk/government/collections/national-child-measurement-programme.

[89] Massachusetts Government. School BMI screenings [internet]. Massachusetts Department of Public Health; 2024 cited [2024 Jan 7]. Available from: https://www.mass.gov/info-details/school-bmi-screenings.

[90] Gillison FB Grey EB McConnell HE Sebire SJ. Using narrative messages to improve parents’ experience of learning that a child has overweight. Br J Child Health 2020;1(5):220–30.

[91] Wild CEK Egli V Rawiri NT Willing EJ Hofman PL Anderson YC. “It’s more personal if you can have that contact with a person”: qualitative study of health information preferences of parents and caregivers of children with obesity in New Zealand. Health Soc Care Community 2022;30(5):e3106–15. 35170827 10.1111/hsc.13756 PMC9545962

[92] Hansen AR Duncan DT Woo Baidal JA Hill A Turner SC Zhang J. An increasing trend in health-care professionals notifying children of unhealthy weight status: NHANES 1999–2014. Int J Obes 2016;40(10):1480–85. 10.1038/ijo.2016.85 27143033

[93] Rose J Glazebrook C Wharrad H Siriwardena AN Swift JA Nathan D Proactive assessment of obesity risk during infancy (ProAsk): a qualitative study of parents’ and professionals’ perspectives on an mHealth intervention. BMC Public Health 2019;19(1):294. 30866879 10.1186/s12889-019-6616-5 PMC6417230

[94] Fuller AB Byrne RA Golley RK Trost SG. Supporting healthy lifestyle behaviours in families attending community playgroups: parents’ perceptions of facilitators and barriers. BMC Public Health 2019;19(1):1740. 31881955 10.1186/s12889-019-8041-1 PMC6935103

[95] Lucas PJ Curtis-Tyler K Arai L Stapley S Fagg J Roberts H. What works in practice: user and provider perspectives on the acceptability, affordability, implementation, and impact of a family-based intervention for child overweight and obesity delivered at scale. BMC Public Health 2014;14(1):614. 24938729 10.1186/1471-2458-14-614 PMC4076754

[96] Michie S Van Stralen MM West R. The behaviour change wheel: a new method for characterising and designing behaviour change interventions. Implement Sci 2011;6(1):42. 21513547 10.1186/1748-5908-6-42 PMC3096582

[97] Schwarzer R Luszczynska A. How to overcome health-compromising behaviors: the health action process approach. Eur Psychol 2008;13(2):141–51.

[98] Flegal KM Ogden CL. Childhood obesity: are we all speaking the same language? Adv Nutr 2011;2(2):159S–166S. 22332047 10.3945/an.111.000307 PMC3065752

[99] Kovacs BE Gillison FB Barnett JC. Is children’s weight a public health or a private family issue? A qualitative analysis of online discussion about National Child Measurement Programme feedback in England. BMC Public Health 2018;18(1):1295. 30477468 10.1186/s12889-018-6214-y PMC6257949

[100] Gutin I. Not ‘putting a name to it’: managing uncertainty in the diagnosis of childhood obesity. Soc Sci Med 2022;294: 114714. 35032744 10.1016/j.socscimed.2022.114714 PMC8821372

[101] Barlow SE Richert M Baker EA. Putting context in the statistics: paediatricians’ experiences discussing obesity during office visits. Child Care Health Dev 2007;33(4):416–23. 17584397 10.1111/j.1365-2214.2006.00716.x

[102] Street RL Makoul G Arora NK Epstein RM. How does communication heal? Pathways linking clinician–patient communication to health outcomes. Patient Educ Couns 2009;74(3):295–301. 19150199 10.1016/j.pec.2008.11.015

[103] Bachmann C Pettit J Rosenbaum M. Developing communication curricula in healthcare education: an evidence-based guide. Patient Educ Couns 2022;105(7):2320–27. 34887158 10.1016/j.pec.2021.11.016

[104] JBI Levels of Evidence and Grades of Recommendation Working Party. JBI levels of evidence [internet]. JBI; 2014 cited [2023 Oct 23]. Available from: https://jbi.global/sites/default/files/2019-05/JBI-Levels-of-evidence_2014_0.pdf.

[105] Vollmer RL. An exploration of how fathers attempt to prevent childhood obesity in their families. J Nutr Educ Behav 2018;50(3):283–288.e1. 29524982 10.1016/j.jneb.2017.12.009

[106] Crouch E Abshire DA Wirth MD Hung P Benavidez GA. Rural–urban differences in overweight and obesity, physical activity, and food security among children and adolescents. Prev Chronic Dis 2023;20:230136. 10.5888/pcd20.230136 PMC10599326 37857462

[107] Rogers R Eagle TF Sheetz A Woodward A Leibowitz R Song M The relationship between childhood obesity, low socioeconomic status, and race/ethnicity: lessons from Massachusetts. Child Obes 2015;11(6):691–95. 26562758 10.1089/chi.2015.0029 PMC4939441

[108] Chatham RE Mixer SJ. Cultural influences on childhood obesity in ethnic minorities: a qualitative systematic review. J Transcult Nurs 2020;31(1):87–99. 31423926 10.1177/1043659619869428

